# Supplementary material for: Spt4 facilitates the movement of RNA polymerase II through the +2 nucleosomal barrier
Source: Cell Rep. 2021 Sep 29;36(13):109755. doi: 10.1016/j.celrep.2021.109755 (PMC8492961; doi:10.1016/j.celrep.2021.109755)
Supplement: Document S1. Figures S1–S7 and Tables S1–S4 [file mmc1.pdf]

Cell Reports, Volume *36*

Supplemental information

Spt4 facilitates the movement of RNA polymerase II through the +2 nucleosomal barrier

Ülkü Uzun, Thomas Brown, Harry Fischl, Andrew Angel, and Jane Mellor

Figure S1, related to Figure 1

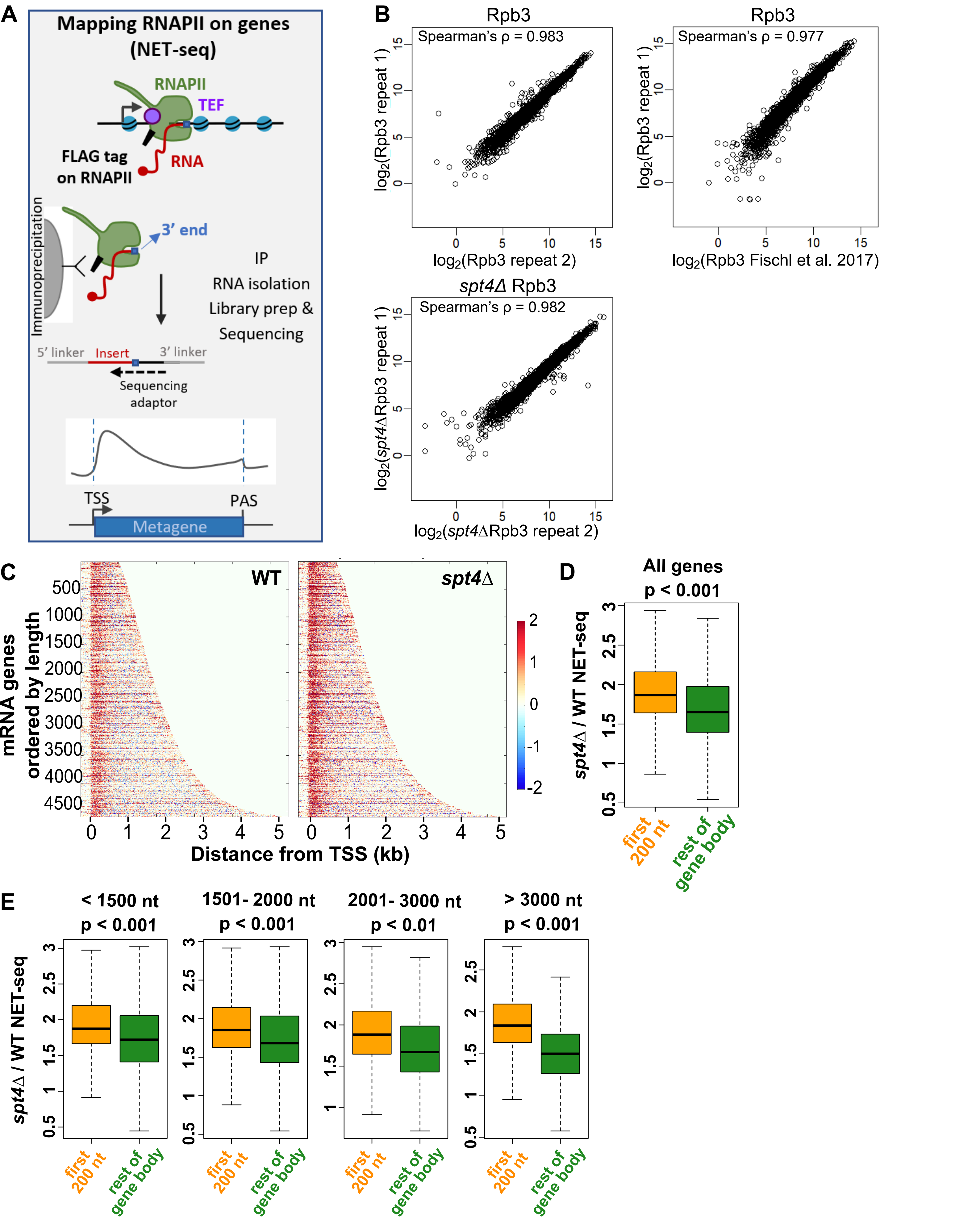

Figure S1, related to Figure 1

In the absence of Spt4, RNAPII accumulates at the 5'end of genes

A Native elongating transcript sequencing (NET-seq) pulls down elongation competent RNAPII and the 3'-end sequencing allows mapping of RNAPII at single nucleotide resolution.

B Correlations between NET-seq repeats from this study and with published NET-seq data from (Fischl et al., 2017). Reads are counted from the TSS to the PAS for each gene. Log_2 transformed gene counts are correlated and Spearman's ρ calculated for each pair.

C Heatmaps of the WT (left) and *spt4*Δ (right) NET-seq signal. Each row indicates a PCG (N=4610), ranked by gene length. The colour code from red to blue reflects the changes in the RNAPII signal for each nucleotide position from TSS-250 nt to TSS+4750 nt (x-axis) as shown by the colour bar.

D Boxplots of the *spt4*Δ / WT NET-seq ratios within the first 200 nt reads from the TSS (from TSS to TSS+200 nt; orange) and the rest of the gene body (from TSS+200 to PAS-250 nt; green) for protein-coding genes after filtering low read genes out (see Methods). N=4610, p <0.001, two-tailed, paired Student's t-test.

E The same analysis in D repeated based on the gene length with the following subgroups < 1500 nt (N=1174), 1501 – 2000 nt (N=1192), 2001-3000 nt (N=1453), and > 3000 nt (N=791).

Figure S2, related to Figure 3

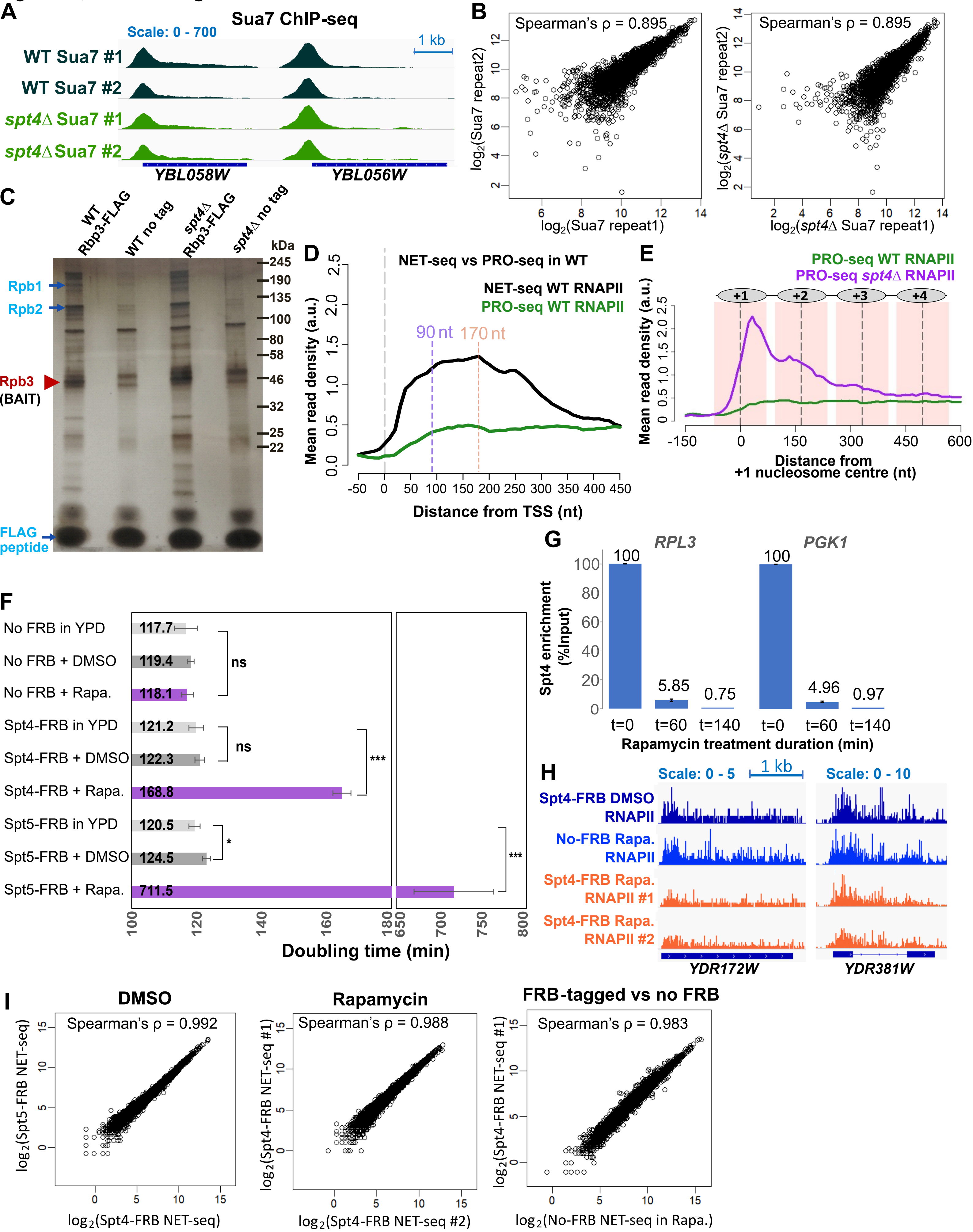

Figure S2, related to Figure 3

The primary defect in *spt4*Δ cells is early transcription elongation

A WT and *spt4*Δ Sua7 ChIP-seq signals of example genes transcribed from the positive strand: *YBL058W* and *YBL056W* in two biological replicates. The dark blue boxes indicate the transcribed region of the genes (from TSS to PAS).

B Correlation plot of the two repeats of each experiment. Reads are counted around the TSS (TSS-100 to TSS+100) for each gene. $\log_2$ transformed gene counts are correlated and Spearman's ρ calculated for each pair.

C Silver staining of purified transcription complexes for proteomics analysis. After purification of the complexes, a small portion of the purified samples was run on 4-20% gradient SDS-PAGE and the protein enrichment was checked using a silver stain.

D Metagene plots of WT NET-seq (black) and WT PRO-seq (green) reads (Booth et al., 2016) aligned at the TSS, the same data as in **Figure 3E**. Dashed lines indicate the highest *spt4*Δ PRO-seq (90 nt, purple) and *spt4*Δ NET-seq reads (170 nt, pink) for comparison with **Figure 3F**.

E Metagene of RNAPII distribution assessed using PRO-seq in *spt4*Δ cells reveals a peak on the upstream face of the +2 nucleosome in addition to a larger peak at +90 nt from the TSS compared to WT (a paused/stalled/backtracked RNAPII that has been released into elongation during the labelling window (see also **Figure 3F**). This peak is also a component of the NET-seq profile, except the RNAPII signal continues to accumulate indicative of a form of RNAPII that is not undergoing active elongation during the labelling window for PRO-seq.

F Doubling times of the anchor away strains. Cells were grown in YDP, DMSO and rapamycin (1 mg/ml in DMSO) for 22 h. OD_{600} was recorded every 20 min using the Bioscreen and doubling times were calculated for exponential growth phase (OD_{600} 0.2 to 0.8) as described in the methods. Error bars indicate standard deviation of 3 biological replicates performed at 4 technical repeats. * p-value <0.05, ***p-value <0.001 (Student's t-test, unpaired, two-tailed).

G ChIP-qPCR for Spt4 upon depletion of Spt4 protein by Anchor Away across different time points. Percentage of Spt4 levels relative to time point 0 levels at the two representative genes *RPL3* and *PGK1* tested by ChIP against GFP (targeting Spt4-FRB-GFP) followed by qPCR. Error bars indicates standard deviation of the two biological replicates.

H DMSO or rapamycin-treated Spt4-FRB or rapamycin-treated No FRB NET-seq signals of example genes transcribed from the positive strand: *YDR172W* and *YDR381W*. Two biological replicates are shown for rapamycin treated cells. The dark blue boxes indicate the transcribed region of the genes (from TSS to PAS), while the blue line indicates the intronic region in *YDR381W.*

I Correlations between anchor away NET-seq repeats and controls. Reads are counted from the TSS to the PAS for each gene. Log_2 transformed gene counts are correlated and Spearman's ρ calculated for each pair.

Figure S3, related to Figure 4

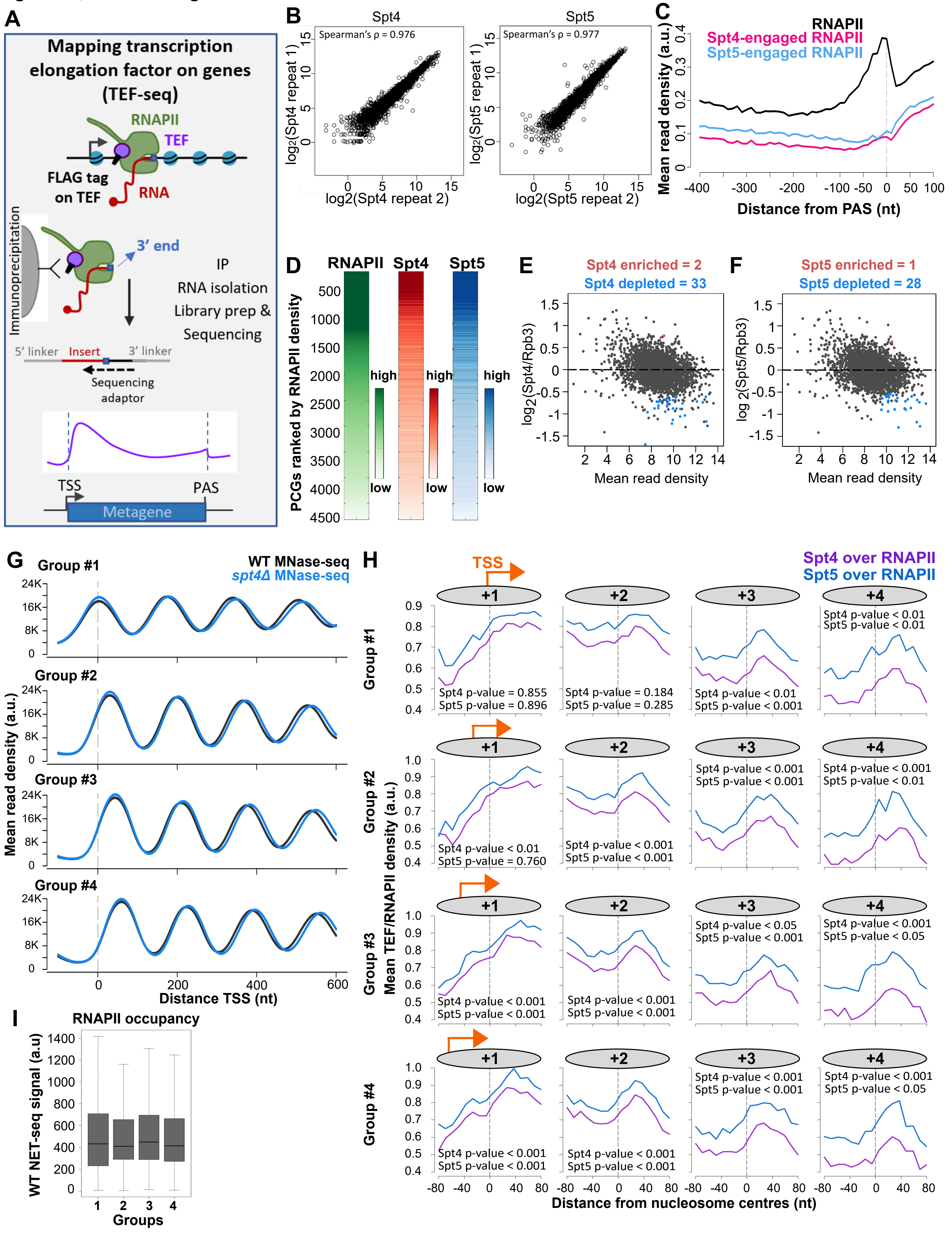

Figure S3, related to Figure 4

Spt4/5 travel with RNAPII and oscillate on and off RNAPII based on the nucleosome positions

A Similar to NET-seq (**Figure S1A**), transcription elongation factor (TEF) associated nascent elongating transcript sequencing (TEF-seq) pulls down elongation competent RNAPII from FLAG-tagged TEF. The 3'-end sequencing maps TEF-associated RNAPII at single nucleotide resolution.

B Correlations between TEF-seq repeats. Reads are counted from the TSS to the PAS for each gene. Log_2 transformed gene counts are correlated and Spearman's ρ calculated for each pair.

C Metagene plots of NET-seq (RNAPII; black), and TEF-seq (Spt4; pink, Spt5; light blue) reads around PAS. (Close up version of **Figure 4B**).

D Heatmaps of RNAPII NET-seq and Spt4/5 TEF-seq reads over the gene bodies (taken as TSS to PAS-250 nt) on log_2 scale. Protein-coding genes are ranked by RNAPII levels.

E, F Differential enrichment of Spt4 (**E**) and Spt5 (**F**) on RNAPII. DEseq2 applied to the read counts from the gene body (TSS to TSS-250 nt) for two replicates of each data. Significantly enriched and depleted genes indicated in red and blue, respectively (p-adjusted <0.05).

G Genes were ranked based on the location of their +1 nucleosome, relative to the transcription start site and split into 4 groups (N=581 per group).

H TEF-seq/NET-seq ratio relative to the centre of +1 to +4 nucleosomes in each of the four groups.

I Boxplots of WT NET-seq reads showing RNAPII occupancy over genes in groups 1 to 4 (see **G**).

Figure S4, related to Figure 5

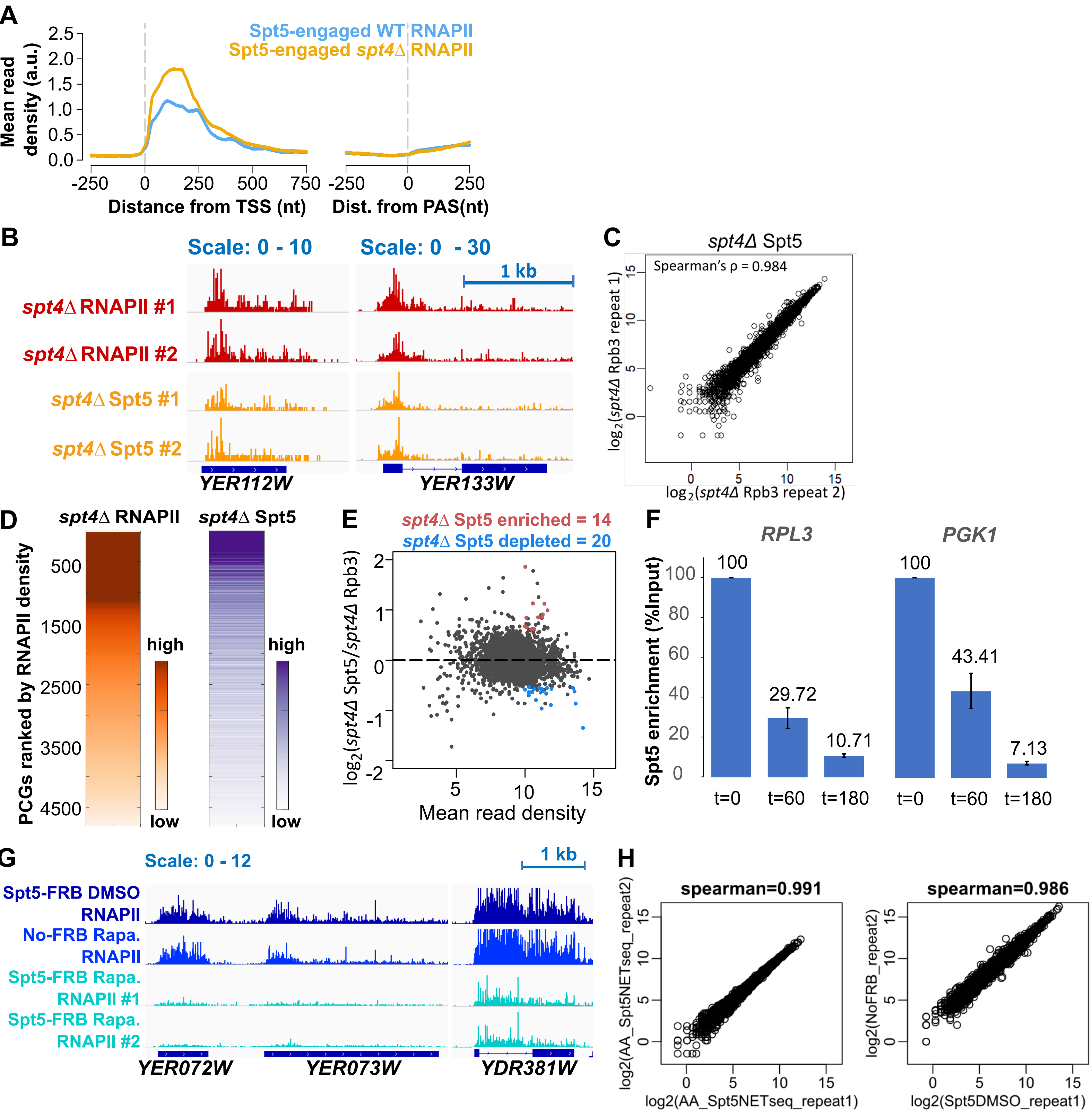

Figure S4, related to Figure 5

Spt5 and Spt4 have distinct impacts on transcription

A Metagene plots of WT Spt5 TEF-seq (light blue; Spt5-engaged WT RNAPII the same as in **Figure 4B**), and *spt4*Δ Spt5 TEF-seq (yellow; Spt5-engaged *spt4*Δ RNAPII) reads aligned at the TSS or PAS.

B *spt4*Δ NET-seq (RNAPII) and *spt4*Δ Spt5 TEF-seq reads of example genes transcribed from the positive strand: *YER112W* and *YER113W* in two biological replicates. The dark blue boxes indicate the transcribed region of the genes (from TSS to PAS), while the blue line indicates the intronic region in *YER113W.*

C Correlations between *spt4*Δ Spt5 TEF-seq repeats. Reads are counted from the TSS to the PAS for each gene. Log_2 transformed gene counts are correlated and Spearman's ρ calculated for each pair.

D Heatmaps of *spt4*Δ RNAPII NET-seq and *spt4*Δ Spt5 TEF-seq reads over the gene bodies (taken as TSS to PAS-250 nt) on log_2 scale. Protein-coding genes are ranked by RNAPII levels.

E Differential enrichment of *spt4*Δ Spt5 on *spt4*Δ RNAPII. DEseq2 applied to the read counts from the gene body (TSS to TSS-250 nt) for two replicates of each data. Significantly enriched and depleted genes indicated in red and blue, respectively (p-adjusted <0.05).

F ChIP-qPCR for Spt5 upon depletion of Spt5 protein by Anchor Away across different time points. Percentage of Spt5 levels relative to time point 0 levels at the two representative genes *RPL3* and *PGK1* tested by ChIP against GFP (targeting Spt5-FRB-GFP) followed by qPCR. Error bars indicates standard deviation of the two biological replicates.

G DMSO or rapamycin-treated Spt5-FRB or rapamycin treated No FRB NET-seq signals of example genes transcribed from the positive strand: *YER072W, YER073W* and *YDR381W*. Two biological replicates are shown for rapamycin treated cells. The dark blue boxes indicate the transcribed region of the genes (from TSS to PAS), while the blue line indicates the intronic region in *YDR381W.*

H Correlations between anchor away NET-seq repeats and controls. Reads are counted from the TSS to the PAS for each gene. Log_2 transformed gene counts are correlated and Spearman's ρ calculated for each pair.

Figure S5, related to Figure 4 and 6

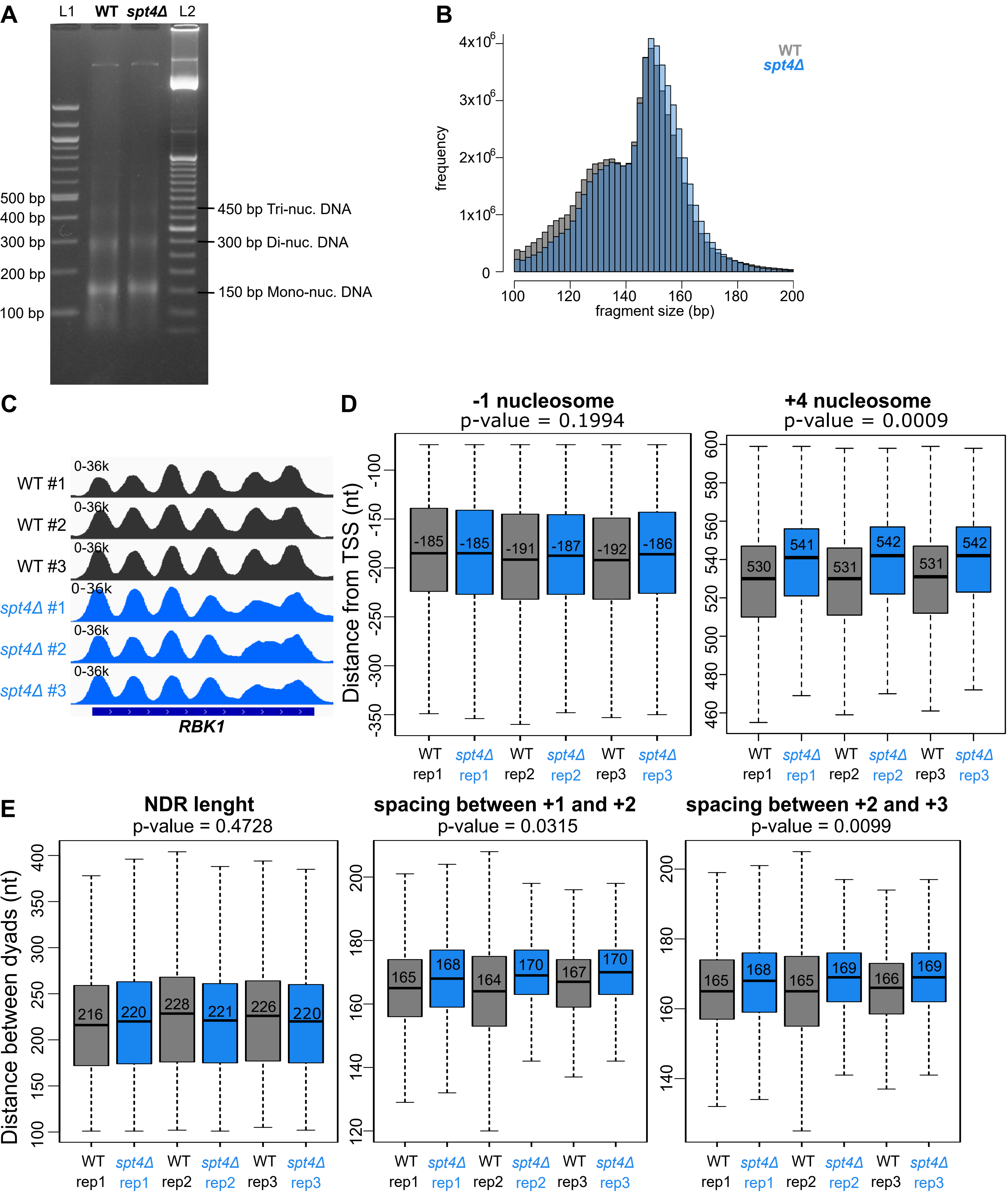

Figure S5, related to Figures 4 and 6
Spt4 influences nucleosome positioning

A WT and *spt4∆* chromatin samples were digested with 80U MNase and separated by 1.5% agarose-TBE gel electrophoresis. 80U MNase digestion led to the optimal digestion pattern as shown in (Chereji et al., 2019). L1: 100 bp ladder, L2: 50 bp ladder. Bands corresponding to mono-nucleosomal DNA (~150 bp) were excised and subjected to paired-end sequencing.

B Histograms of MNase-seq fragment size in WT (grey) and *spt4∆* (blue) data sets. Consistent with the digestion pattern in A, the majority of the sequenced fragments represent mono-nucleosomal fragments (~150 bp) in both data sets, giving the optimal digestion pattern.

C MNase-seq reads of example genes transcribed from the positive strand (*RBK1*) in WT and *spt4∆* in 3 biological replicates. The dark blue boxes indicate the transcribed region of the genes (from TSS to PAS).

D Box-plots of the distance of the -1 and +4 nucleosomes from the TSS in three biological replicates of WT (black) and *spt4∆* cells (blue). Numbers in the boxes indicate the median position of the given nucleosome. p-values were calculated by comparing the median position of the -1 or +4 nucleosomes in WT and *spt4∆* conditions obtained from each replicate (Student's t-test, paired, two sided).

E Box-plots of the distance between the -1 and +1 (NDR length), +1 and +2, +2 and +3 nucleosomes in three biological replicates of WT (black) and *spt4∆* cells (blue). Numbers in the boxes indicate the median distance between the indicated nucleosomes. p-values were calculated by comparing the median distances between the nucleosomes in WT and *spt4∆* conditions obtained from each replicate (Student's t-test, paired, two sided).

Figure S6, related to Figure 7

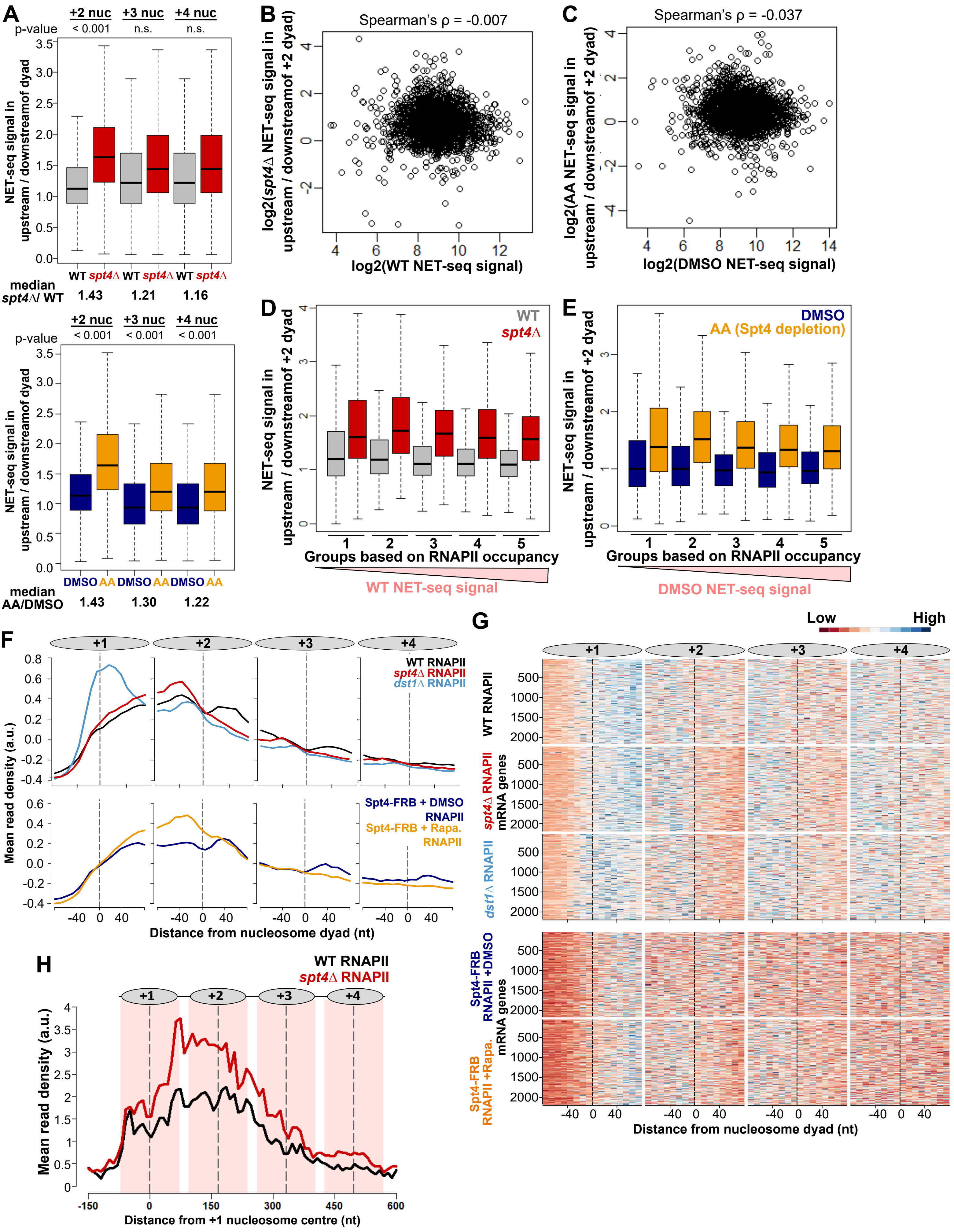

Figure S6, related to Figure 7

The accumulation of RNAPII in the absence of Spt4 is associated with the position of the +2 nucleosome

A The WT (grey), *spt4*Δ (red), DMSO (navy), and Spt4 Anchor Away (AA; orange) RNAPII signal ratio from upstream of the dyad (-60 to -10 nt from the dyad) to downstream of the dyad (+10 to +60 nt from the dyad) were shown as boxplots for the +2, +3, and +4 nucleosomes. The difference of the accumulation in WT and *spt4*Δ or DMSO and Spt4 Anchor Away cells were tested by two-tailed, paired Student's t-test. To compare different nucleosomes, *spt4*Δ/WT or AA/DMSO RNAPII accumulation ratios around the +2, +3, and +4 nucleosomes are given at the bottom of the plots.

B,C Correlation plots showing the relationship between reads on the upstream or downstream face of the +2 nucleosome for *spt4*Δ (**B**) or Spt4-AA (**C**) and levels of transcription over each gene. $\log_2$ transformed gene counts are correlated for each gene and Spearman's ρ calculated. **D,E** 2312 genes were divided into 5 groups using the WT RNAPII signal (NET-seq) over the gene bodies after genes with very low NET-seq signal and with poor nucleosome phasing were filtered out as described in the Methods. RNAPII signal ratio of the proximal to distal reads around the +2 dyad was assessed in the *spt4Δ* strain compared to WT (**D**) or after Spt4 depletion (AA) compared to the DMSO control (**E**).

F Metagene plots of WT (black), *spt4*Δ (red), and *dst1*Δ (blue) (top panel) and DMSO-treated (navy), and rapamycin-treated Spt4-FRB (orange) (bottom panel) NET-seq profiles around the +1, +2, +3 and, +4 nucleosome dyads. Dashed lines (black) through the peaks indicate the centres of the nucleosomes (-/+ 80 nt from the dyad; x-axis). The NET-seq reads were normalised to the mean and standard deviation of each gene to indicate the shape of the distribution of RNAPII regardless of the expression level differences between the genes (N=2212), also shown in **G**. Position of nucleosomes graphically shown above the metagene plot.

G Heatmaps of WT, *spt4*Δ, *dst1*Δ, DMSO-treated, and rapamycin-treated Spt4-FRB NET-seq profiles around the +1, +2, +3 and, +4 nucleosomes. Each row indicates a PCG (n=2212). RNAPII signal is shown in 10 nt bins around the indicated nucleosome dyads (-/+ 80 nt from the dyad; x-axis). The NET-seq reads were normalised to the mean and standard deviation of each gene to indicate the shape of the distribution of RNAPII regardless of the expression level differences between the genes.

H Metagene plots of WT (black) and *spt4*Δ (red) NET-seq profiles from 43 genes with reduced Sua7 (TFIIB) occupancy at the promoter in *spt4Δ* (See **Figure 3B**), around the +1, +2, +3 and, +4 nucleosome dyads. Dashed lines (black) through the peaks indicate the centres of the nucleosomes (-/+ 80 nt from the dyad; x-axis). The NET-seq reads were normalised to the mean and standard deviation of each gene to indicate the shape of the distribution of RNAPII regardless of the expression level differences between the genes.

Figure S7, related to Figure 7

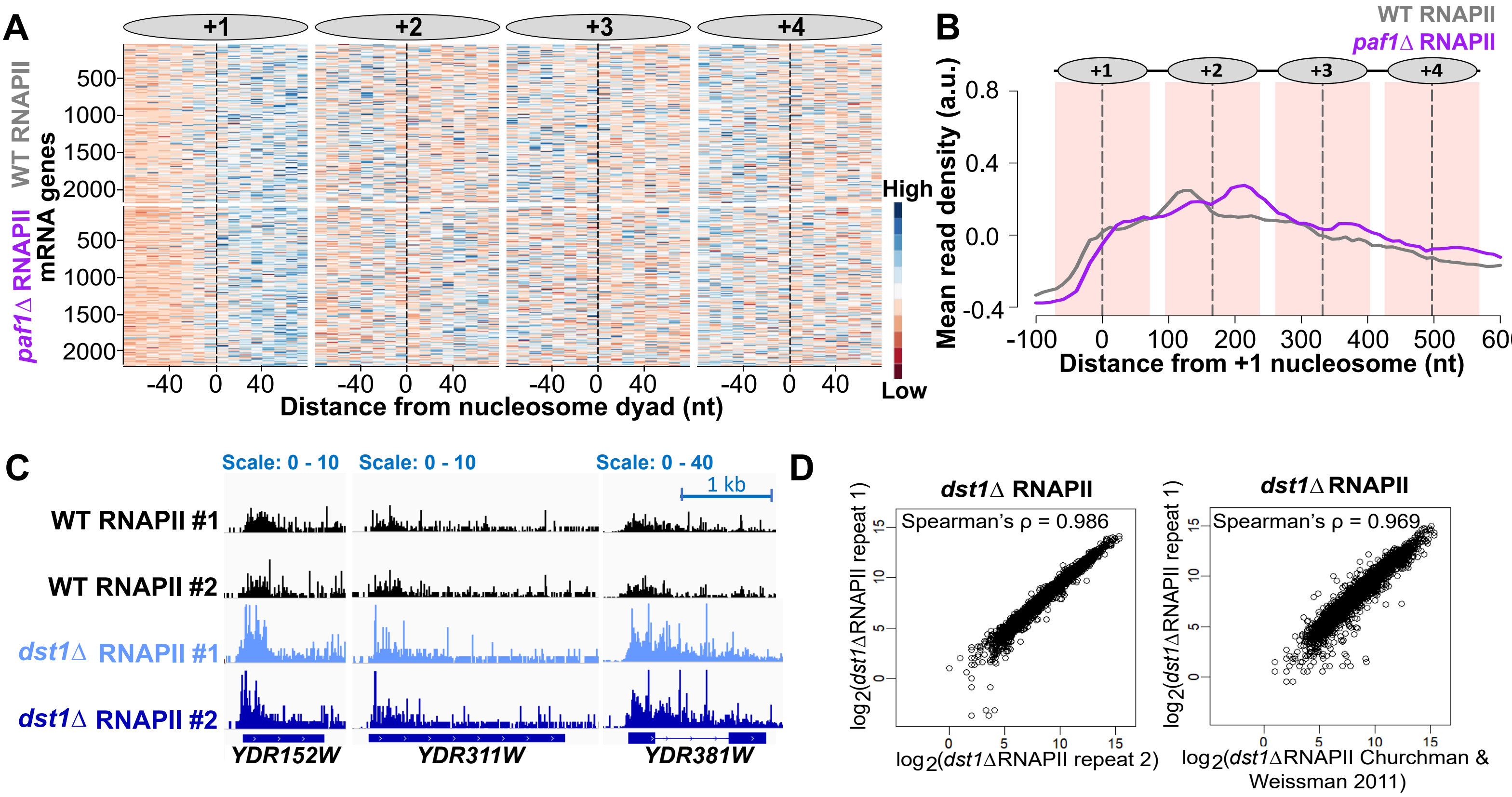

Figure S7 related to Figure 7

Comparison of the effect of loss of Paf1 and Dst1 on RNAPII accumulation

A Heatmaps of WT (top) and *paf1*Δ (bottom) NET-seq profiles around the +1, +2, +3 and, +4 nucleosomes. Each row indicates a PCG (N=2212). RNAPII signal is shown in 10 nt bins around the indicated nucleosome dyads (-/+ 80 nt from the dyad; x-axis). The NET-seq reads were normalised to the mean and standard deviation of each gene, so that the shape of the distribution of RNAPII could be seen more clearly regardless of the expression level differences between the genes. NET-seq datasets were taken from ArrayExpress: E-MTAB-4568 (Fischl et al., 2017).

B Metagene plots of WT (grey) and *paf1*Δ (purple) NET-seq profiles relative to the +1 nucleosome dyad of the same data as in **A**. The mean and standard deviation normalised reads were used for plotting metagene profiles, as the global comparison of the NET-seq levels were not available for these datasets. Dashed lines (black) through the peaks indicate the centres of the nucleosomes and the nucleosomal DNA (+/- 70 nt around the centre) is highlighted in light pink.

C NET-seq (RNAPII) reads of example genes transcribed from the positive strand: *YDR152W, YDR331W,* and *YDR381W* in two biological replicates in WT and *dst1*Δ cells. The dark blue boxes indicate the transcribed region of the genes (from TSS to PAS), while the blue line indicates the intronic region in *YDR381W.*

D Correlations between NET-seq repeats in *dst1*Δ cells from this study and with published NET-seq data from (Churchman and Weissman, 2011). Reads are counted from the TSS to the PAS for each gene. Log_2 transformed gene counts are correlated and Spearman's ρ calculated for each pair.

Table S1. Related to Figure 2
The maximum and minimum parameter values for the latin hypercube sampling

Parameter	Min Value	Max Value
Initiation Rate	0.01	60
Elongation Rate	100	5000
Stall 1 Rate	0.01	60
Stall Restart 1 Rate	0.01	60
Backtrack 1 Rate	0.01	60
Backtrack Restart 1 Rate	0.01	60
Stall 2 Rate	0.01	60
Stall Restart 2 Rate	0.01	60
Backtrack 2 Rate	0.01	60
Backtrack Restart 2 Rate	0.01	60
Location of Window Boundary	0	1000
Location of Early Termination	0	1000

Table S2. Related to Figure 3
Mass spec log fold enrichment analysis results

name	*spt4*Δ vs WT p.val	*spt4*Δ vs WT p.adj	*spt4*Δ vs WT ratio
Spt4	0.001	0.000	-3.690
Set2	0.021	0.000	-2.580
Spn1	0.047	0.003	-1.850
Spt16	0.032	0.001	-1.740
Pob3	0.063	0.007	-1.490
His3	0.449	0.414	-1.470
Gpn2	0.263	0.185	-1.260
Spt6	0.157	0.062	-1.250
Tma19	0.183	0.087	-1.170
Sub2	0.320	0.252	-1.150
Prp39	0.351	0.290	-1.110
Yra1	0.266	0.188	-1.100
Prp42	0.339	0.275	-1.070
Rpb3	0.242	0.158	-1.060
Bre1	0.496	0.466	-1.020
Rpc10	0.169	0.073	-0.989
Wtm1	0.241	0.157	-0.986
Asr1	0.474	0.442	-0.985
Hsh155	0.370	0.315	-0.953
Tex1	0.304	0.234	-0.835
Spt5	0.323	0.256	-0.767
Rse1	0.543	0.513	-0.754
Snu71	0.480	0.449	-0.718
Set1	0.718	0.645	-0.706
Prp40	0.514	0.485	-0.659
Tho2	0.571	0.539	-0.599
Smb1	0.571	0.539	-0.587
Rpb11	0.442	0.405	-0.566
Rba50	0.632	0.588	-0.563
Smd2	0.440	0.403	-0.547
Dst1	0.692	0.630	-0.522
Luc7	0.759	0.668	-0.492
Rpo21	0.548	0.518	-0.488
Hpr1	0.571	0.538	-0.466
Prp19	0.571	0.539	-0.461
Rpb2	0.575	0.542	-0.459
Snu114	0.722	0.648	-0.436
Hta2	0.576	0.543	-0.407
Rpb5	0.622	0.580	-0.346
Yhc1	0.707	0.638	-0.328
Smd3	0.688	0.627	-0.316
Rat1	0.714	0.643	-0.309
Rtt103	0.685	0.625	-0.291
Mft1	0.757	0.667	-0.278
Cbc2	0.731	0.653	-0.271
Rpb4	0.733	0.654	-0.245
Rpb8	0.735	0.655	-0.241
Rpo26	0.816	0.697	-0.162
Rpc19	0.864	0.717	-0.157
Thp2	0.891	0.728	-0.136
Tfg1	0.868	0.719	-0.127
Rpb9	0.880	0.724	-0.113
Sdc1	0.921	0.738	-0.100
Snu56	0.941	0.746	-0.087
Ceg1	0.968	0.754	-0.031
Mud1	0.986	0.760	-0.025

Sto1	0.991	0.761	-0.011
Ess1	0.994	0.762	-0.008
Rai1	0.985	0.759	0.030
Rpb7	0.963	0.752	0.032
Npa3	0.964	0.753	0.040
Tfg2	0.929	0.741	0.086
Smt3	0.794	0.686	0.178
Cet1	0.772	0.675	0.215
Rtr1	0.887	0.726	0.250
Abd1	0.774	0.676	0.320
Gpn3	0.680	0.622	0.443
Rad23	0.620	0.579	0.460
Taf14	0.538	0.508	0.494
Glc7	0.492	0.462	0.588
Ccl1	0.672	0.616	0.598
Bud27	0.416	0.374	0.729
Brr2	0.532	0.503	0.814
Yta7	0.281	0.207	1.270
Ald5	0.073	0.010	1.660
Ssl1	0.083	0.013	1.720
Sua7	0.094	0.017	1.930
Iwr1	0.102	0.020	1.960
Tfb2	0.130	0.039	2.120
Rad3	0.098	0.019	2.210
Tfb1	0.079	0.012	2.510
Tfb3	0.015	0.000	2.600
Ssl2	0.048	0.003	2.980
Yke2	0.026	0.000	4.120

Table S3. Related to STAR methods
Yeast strains used in this study

Strain	Source	Genotype
BY4741	Euroscarf	*MATa; his3Δ1; leu2Δ0; met15Δ0; ura3Δ0*
BY4741 *spt4::KanMX6 (SPT4 KO)*	Euroscarf	*spt4::KanMX6*
BY4741 Rpb3-FLAG (WT)	Fischl et al. 2017	*RPB3-3xFLAG-His3MX6*
BY4741 *SPT4 KO* Rpb3-FLAG (*spt4Δ*)	This study	*RPB3-3xFLAG-His3MX6; spt4::KanMX6*
BY4741 Spt4-FLAG	This study	*SPT4-3xFLAG-His3MX6*
BY4741 Spt5-FLAG	This study	*SPT5-3xFLAG-His3MX6*
BY4741 Sua7-FLAG	This study	*SUA7-3xFLAG-His3MX6*
BY4741 *SPT4 KO* Sua7-FLAG	This study	*SUA7-3xFLAG-His3MX6; spt4::KanMX6*
S.pombe Rpb9-FLAG	L. Vasileva	*u+; leu1-32; ura4Δ18; ade16-M216; his3Δ1; RPB9-3xFLAG-KanMX4*
AA Spt4-FRB-GFP Rpb3-FLAG	This study	*tor1-1; Δfpr1; RPL13-2xFKBP12-NATMX6; met15; LYS2; his3-1; leu2; ura3; MATa; SPT4-FRB-eGFP-HygMX*
AA Spt5-FRB-GFP Rpb3-FLAG	This study	*tor1-1; Δfpr1; RPL13-2xFKBP12-NATMX6; met15; LYS2; his3-1; leu2; ura3; MATa; SPT5-FRB-eGFP-HygMX*
AA Rpb3-FLAG (No FRB)	This study	*tor1-1; Δfpr1; RPL13-2xFKBP12-NATMX6; met15; LYS2; his3-1; leu2; ura3; MATa*
BY4741 *dst1::kanMX6*	Euroscarf	*dst1::kanMX6*
BY4741 *dst1::kanMX6* Rpb3-FLAG	This study	*RPB3-3xFLAG-His3MX6; dst1::kanMX6*

Table S4. Related to STAR methods
Oligonucleotides used in this study

Oligonucleotide	Sequence
Linker-1	5’AppCTGTAGGCACCATCAAT/3ddC 3’
RNA control oligo	5′ agu cac uua gcg aug uac acu gac ugu g3′
RT primer	5’/5Phos/ATCTCGTATGCCGTCTTCTGCTTG/iSp18/CACTCA/iSp18 /TCCGACGATCATTGATGGTGCCTACAG 3’
Barcoding reverse	5’CAAGCAGAAGACGGCATACGA 3’
Barcode-1	5’AATGATACGGCGACCACCGAGATCTACACGATCGGAAGAGC ACACGTCTGAACTCCAGTCACATGCCATCCGACGATCATTGAT GG 3’
Barcode-2	5’AATGATACGGCGACCACCGAGATCTACACGATCGGAAGAGC ACACGTCTGAACTCCAGTCACTGCATCTCCGACGATCATTGAT GG 3’
Barcode-4	5’AATGATACGGCGACCACCGAGATCTACACGATCGGAAGAGC ACACGTCTGAACTCCAGTCACTTAGGCTCCGACGATCATTGAT GG 3’
Barcode-5	5’AATGATACGGCGACCACCGAGATCTACACGATCGGAAGAGC ACACGTCTGAACTCCAGTCACTGACCATCCGACGATCATTGAT GG 3’
Barcode-6	5’AATGATACGGCGACCACCGAGATCTACACGATCGGAAGAGC ACACGTCTGAACTCCAGTCACGCCAATTCCGACGATCATTGAT GG 3’
Barcode-7	5’AATGATACGGCGACCACCGAGATCTACACGATCGGAAGAGC ACACGTCTGAACTCCAGTCACCAGATCTCCGACGATCATTGAT GG 3’
Barcode-8	5’AATGATACGGCGACCACCGAGATCTACACGATCGGAAGAGC ACACGTCTGAACTCCAGTCACACTTGATCCGACGATCATTGAT GG 3’
Barcode-9	5’AATGATACGGCGACCACCGAGATCTACACGATCGGAAGAGC ACACGTCTGAACTCCAGTCACGATCAGTCCGACGATCATTGAT GG 3’
Barcode-10	5’AATGATACGGCGACCACCGAGATCTACACGATCGGAAGAGC ACACGTCTGAACTCCAGTCACTAGCTTTCCGACGATCATTGAT GG 3’
Sequencing primer	5’TCCGACGATCATTGATGGTGCCTACAG 3’
RPL3_forward	5’ GGGTTACAAG GCTGGTATGA 3’
RPL3_reverse	5’ ACAACGACAACTGGTGGAGT 3’
PGK1_forward	5’ GCGTGTCTTC ATCAGAGTTG 3’
PGK1_reverse	5’AGTGAGAAGCCAAGACAACG 3’
RPB3_Cterm_FLAG_Fo rward	5’AATGGGTAATACTGGATCAGGAGGGTATGATAATGCTTGGA CTAGTGGATCCCCCGGGGAT 3’
RPB3_Cterm_FLAG_Re verse	5’TTTCGGTTCGTTCACTTGTTTTTTTCCTCTATTACGCCCGAA TTCGAGCTCGTTTAAAC 3’
SPT5-Cterm-FLAGtag-Forward	5’TAAGTCAAACTATGGTGGTAACAGTACATGGGGAGGTCATA CTAGTGGATCCCCCGGGGAT 3’
SPT5_Cterm_FRBtag_F orward	5’TAAGTCAAACTATGGTG GTAACAGTACATGGGGA GGTCATCGGATCCCCGG GTTAATTAA 3’
SPT5-Cterm-tag-Reverse	5’GTCTTTTTTATTGATTTCTTCTTGGGTGATATTGGTTCTCGAA TTCGAGCTCGTTTAAAC 3’
SUA7_Cterm_FLAG_Fo rward	5’TGTAGTGTCTTTGGATAACTTACCGGGCGTTGAAAAGAAAA CTAGTGGATCCCCCGGGGAT 3’
SUA7_Cterm_FLAG_Re verse	5’TCTACCCTCTAACACGAGTACCCGTGCTTCTTGTTCCTATGA ATTCGAGCTCGTTTAAAC 3’
SPT4-Cterm-FRBtag-Forward	5’GTTGCCTCACTACAAACCGAGGGATGGCAGTCAA GTTGAGCGGATCCCCGGGTTAATTAA 3’
SPT4-Cterm-tag-Reverse	5’ATTCATTACTATTATACATGTGATATCAGAACGGAAGGTTGA ATTCGAGCTCGTTTAAAC 3’
