# Supplementary material for: Spt4 facilitates the movement of RNA polymerase II through the +2 nucleosomal barrier
Source: Cell Rep. 2021 Sep 29;36(13):109755. doi: 10.1016/j.celrep.2021.109755 (PMC8492961; doi:10.1016/j.celrep.2021.109755)
Supplement: Document S2. Article plus supplemental information [file mmc2.pdf]

Cell Reports

Article

Spt4 facilitates the movement of RNA polymerase II through the +2 nucleosomal barrier

Graphical abstract

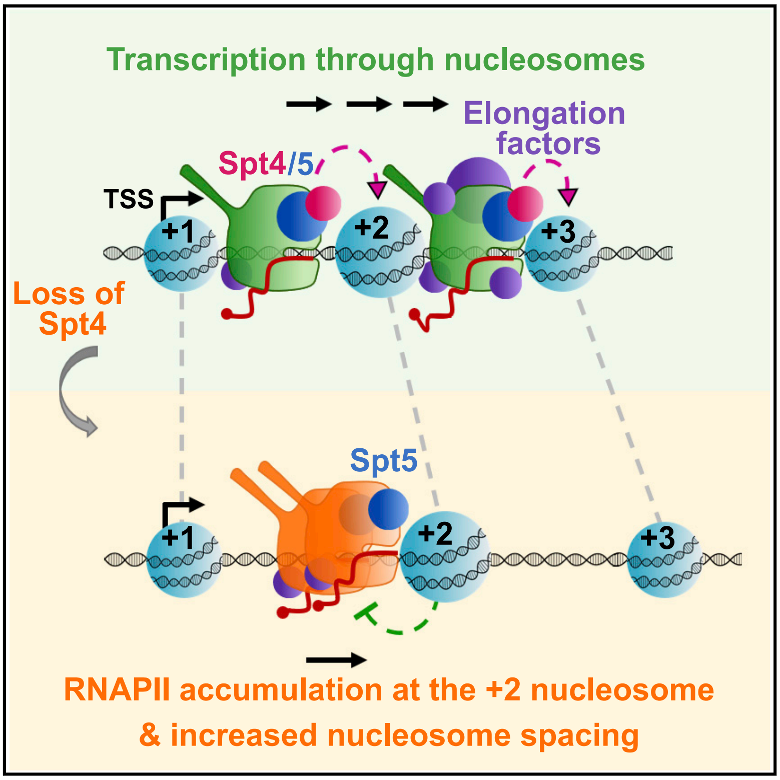

Highlights

- Spt4 regulates transcription dynamics in early elongation
- Spt4/5 oscillate on and off RNAPII based on the position of nucleosomes
- Spt4 affects co-transcriptional nucleosome positioning of the gene-body nucleosomes
- Transcription through the +2 nucleosomal barrier requires Spt4

Authors

Ülkü Uzun, Thomas Brown, Harry Fischl, Andrew Angel, Jane Mellor

Correspondence

jane.mellor@bioch.ox.ac.uk

In brief

During eukaryotic transcription, RNA polymerase II (RNAPII) must overcome physical barriers set by nucleosomes. Here, Uzun et al. report that Spt4, a conserved elongation factor, participates in co-transcriptional nucleosome positioning and promotes RNAPII movement through nucleosomes, especially at the +2 nucleosome.

Uzun et al., 2021, Cell Reports *36*, 109755
September 28, 2021 © 2021 The Authors.
https://doi.org/10.1016/j.celrep.2021.109755

CellPress

Cell Reports

CellPress
OPEN ACCESS

Article

Spt4 facilitates the movement of RNA polymerase II through the +2 nucleosomal barrier

Ülkü Uzun,[1] Thomas Brown,[1] Harry Fischl,[1] Andrew Angel,[1] and Jane Mellor[1,2,*]
[1]Department of Biochemistry, University of Oxford, South Parks Road, Oxford OX1 3QU, UK
[2]Lead contact
*Correspondence: jane.mellor@bioch.ox.ac.uk
https://doi.org/10.1016/j.celrep.2021.109755

SUMMARY

Spt4 is a transcription elongation factor with homologs in organisms with nucleosomes. Structural and *in vitro* studies implicate Spt4 in transcription through nucleosomes, and yet the *in vivo* function of Spt4 is unclear. Here, we assess the precise position of Spt4 during transcription and the consequences of the loss of Spt4 on RNA polymerase II (RNAPII) dynamics and nucleosome positioning in *Saccharomyces cerevisiae*. In the absence of Spt4, the spacing between gene-body nucleosomes increases and RNAPII accumulates upstream of the nucleosomal dyad, most dramatically at nucleosome +2. Spt4 associates with elongating RNAPII early in transcription, and its association dynamically changes depending on nucleosome positions. Together, our data show that Spt4 regulates early elongation dynamics, participates in co-transcriptional nucleosome positioning, and promotes RNAPII movement through the gene-body nucleosomes, especially the +2 nucleosome.

INTRODUCTION

In eukaryotes, nucleosomes limit access to DNA and thus act as intrinsic barriers to DNA-dependent processes including RNA polymerase II (RNAPII) transcription (Kornberg, 1974; Zhou et al., 2019). Transcription requires the sequential breaking of interactions between nucleosomal DNA and histones and reassembling the nucleosome after RNAPII has passed (Kujirai and Kurumizaka, 2020). A wide range of factors are implicated in assisting RNAPII through nucleosomes, but how they function in the cell is not yet clear (Clapier et al., 2017; Ehara et al., 2019; Farnung et al., 2018; Gurova et al., 2018; Venkatesh and Workman, 2015). One such factor is the DRB (5,6-dichloro-1-β-D-ribofuranosylbenzimidazole)-sensitivity inducing factor (DSIF) complex in metazoans, also known as the Spt4/5 complex in yeasts, which is required for efficient transcription on chromatin (Crickard et al., 2017; Ehara et al., 2019; Kujirai and Kurumizaka, 2020; Vos et al., 2020). Spt4 is one of the most highly conserved transcription elongation factors (TEFs) in archaea and eukaryotes, and its partner Spt5 is conserved in all three kingdoms (Hartzog and Fu, 2013; Ponting, 2002). Structural studies demonstrated that the Spt4/5 complex locates on top of the RNAPII active cleft and in between the upcoming nucleosome and RNAPII (Ehara et al., 2019; Farnung et al., 2018), *in vitro* transcription assays revealed that the Spt4/5 complex reduces RNAPII stalling during transcription through a nucleosome (Crickard et al., 2017; Ehara et al., 2019), and single-molecule experiments showed that the Spt4/5 complex differentially interacts with RNAPII-nucleosome intermediates (Crickard et al., 2017). Despite these studies implicating Spt4/5 in transcription regulation in the context of chromatin, the *in vivo* functions of Spt4/5 remain poorly understood. Furthermore, most studies focus on Spt4/5 as a complex that makes it hard to interpret the exact function of Spt4 and Spt5 as individual TEFs (Decker, 2021).

Here, we show a primary function for Spt4 in early transcription elongation in the cell. The association between Spt4 and RNAPII dynamically changes as RNAPII transitions through nucleosomes and gene-body nucleosome positions (from the +2 nucleosome onward) are altered in *spt4Δ* cells. In both *spt4Δ* and cells depleted of Spt4 in real time, RNAPII accumulates upstream of nucleosome dyads, especially at the +2 nucleosome. Overall, these findings support Spt4 promoting RNAPII movement through nucleosomes, especially in early transcription, and regulating co-transcriptional nucleosome positioning.

RESULTS

In the absence of Spt4, RNAPII accumulates at the 5′ end of genes

As Spt4 is an elongation factor, we asked whether Spt4 influences the genome-wide distribution of RNAPII by using native elongating transcript sequencing (NET-seq) that maps the position of RNAPII with RNA in the active site. Spike-in normalized NET-seq was performed in wild-type (WT) and *spt4* knockout (*spt4Δ*) cells expressing FLAG-tagged RNAPII in biological duplicates. To remove the background signal, samples without FLAG tag (no-tag control) were processed in parallel. NET-seq repeats were reproducible and consistent with the previously published

This is an open access article under the CC BY license (http://creativecommons.org/licenses/by/4.0/).

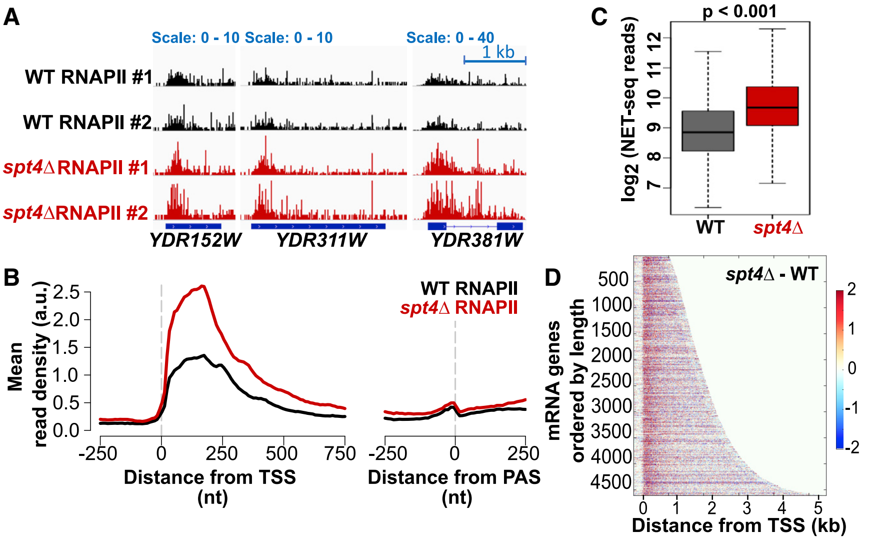

Figure 1. In the absence of Spt4, RNAPII accumulates at the 5′ end of genes

(A) WT and *spt4Δ* NET-seq signals of example genes transcribed from the positive strand, namely, *YDR152W*, *YDR311W*, and *YDR381W*, in two biological replicates. The dark-blue boxes indicate the transcribed region of the genes (from transcription start site [TSS] to PAS), and the blue line indicates the intronic region in *YDR381W*.

(B) Metagene plots of NET-seq reads in WT (black) and *spt4Δ* (red) aligned at the TSS or PAS.

(C) Boxplots of the NET-seq reads in WT (gray) and *spt4Δ* (red) cells on s $\log_2$ scale. The reads were counted from TSS to PAS-250 nt for protein-coding genes after filtering out low-read genes (see STAR Methods). n = 4,610; p < 0.001, two-tailed, paired Student's t test.

(D) Heatmaps of the difference between the *spt4Δ* and WT NET-seq signal (*spt4Δ*-WT). Each row indicates a protein-coding gene (n = 4,610), ranked by gene length. The color code reflects the changes in the RNAPII signal for each nucleotide position from TSS-250 nt to TSS+4,750 nt (x axis) as shown by the color bar.

NET-seq data (Churchman and Weissman, 2011; Fischl et al., 2017; Figures S1A and S1B).

In WT cells, the NET-seq signal is relatively high within the first 500 nucleotides (nt) from the transcription start site (TSS), and then it drops while transcribing over the gene body and peaks again upstream of the polyadenylation site (PAS) (Figures 1A and 1B). In *spt4Δ* cells, the density of RNAPII significantly increased over genes (Figures 1A–1C). Importantly, the most apparent change in the distribution of RNAPII was within the first 200 nt from the TSS regardless of gene length (Figures 1D and S1C–S1E), suggesting that Spt4 regulates the distribution of RNAPII early in transcription.

Mathematical modeling supports defects in early transcription elongation in *spt4Δ* cells

To address potential mechanisms leading to the higher RNAPII signal in *spt4Δ* than that in WT cells, we developed a mathematical model designed to simulate the shape of the NET-seq profiles for which a number of potential mechanisms occurring during transcription are ascribed relative numerical values (Figure 2; Table S1). This model enabled us to relate changes to the profiles of *spt4Δ* compared with WT cells to underlying transcription dynamics. The model computationally simulated RNAPII dynamics by considering initiation, elongation, occlusion of RNAPII by a downstream RNAPII, collision of RNAPIIs, stalling, backtracking, resolution of collision/backtracking/stalling events, and early termination. In contrast to previous approaches (Azofeifa and Dowell, 2017; Fischer et al., 2020; Tufegdžić Vidaković et al., 2020), we set two distinct windows of transcription in which stalling and backtracking parameters can be different (Figure 2A), as RNAPII is subject to distinct regulation in the early and late stages of transcription (Peck et al., 2019). The RNAPII position provided by experimental NET-seq data was fitted to the shape of the transcription profile simulated by the model (Figure 2B). Modeling suggested that there are six key metrics that can be inferred from the shape of the WT RNAPII distribution, as follows: (1) ratio of the rate of initiation compared to elongation, (2) ratio of RNAPII moving compared to stalled or backtracked (moving ratio) in window 1, (3) the size of window 1, (4) the mean location of early termination, (5) the moving ratio in window 2, and (6) the processivity of RNAPII (% of initiating polymerase reaching 1,000 nt). To test the extent of the change in each metric, the parameter values were obtained for each gene in WT and *spt4Δ* cells and the two conditions were quantitatively compared (Figure 2C). Three parameter values showed significant differences in *spt4Δ* compared with those in the WT. The increase in the initiation-to-elongation ratio suggested either a defect in elongation or an increased initiation frequency in the profiles from *spt4Δ* cells. An overall defect in elongation is supported by a reduced proportion of moving polymerase in window 1 and a reduction in the processivity of polymerase in *spt4Δ* cells compared with that in WT cells.

The primary defect in *spt4Δ* cells is early transcription elongation

Modeling suggests that the movement of RNAPII in the early stages of transcription is the main defect in *spt4Δ* cells but cannot distinguish a defect at initiation from early elongation or both. To examine and validate the predictions of the model, we (1) investigated the levels of the pre-initiation complex (PIC) at promoters as a proxy for transcription initiation frequency, (2) investigated the composition of the transcription complex, (3) compared elongation competent RNAPII with levels of all engaged RNAPII, and (4) mapped RNAPII upon rapid depletion of Spt4 to detect the immediate changes in the distribution of RNAPII. Our data support a primary function for Spt4 in early transcription elongation, rather than initiation.

Sua7 (TFIIB) is a subunit of the PIC required for RNAPII recruitment to promoters (Sainsbury et al., 2015), and the amount of chromatin-bound Sua7 reflects the changes in transcription initiation levels (Cucinotta et al., 2021; Doris et al., 2018). Therefore, if the transcription initiation rate was higher in *spt4Δ* cells, levels

CellPress
OPEN ACCESS

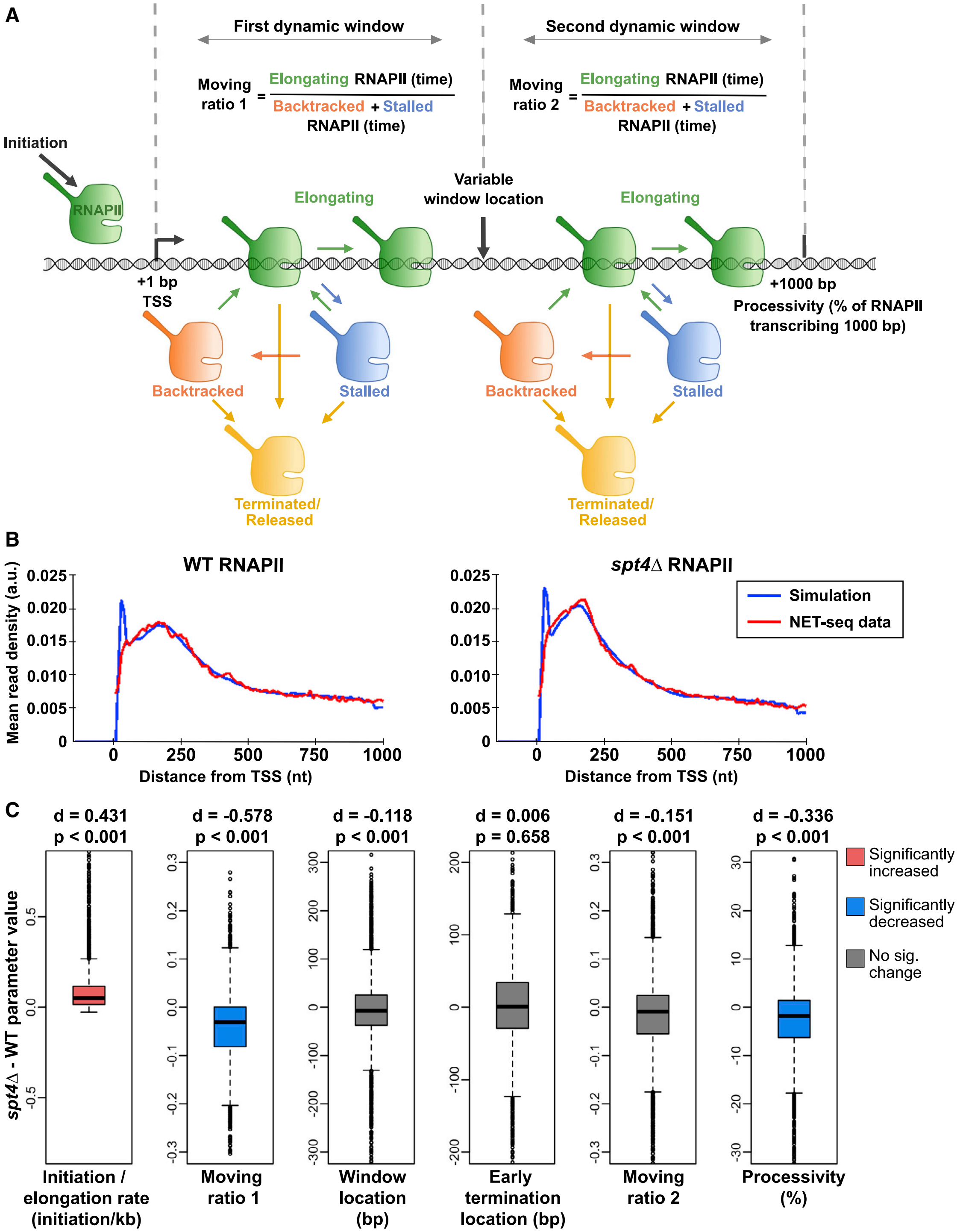

Figure 2. Mathematical modeling supports defects in early transcription elongation in *spt4Δ* cells

(A) Schematic of the mathematical model. Model describes RNAPII transcription reaching 1,000 bp with initiation rate (initiation min^{-1}), elongation rate (kb min^{-1}), stalling, backtracking, termination events (determined by Poisson distribution), and variable window location (bp). Moving ratio 1 and 2 describes the number of RNAPIIs elongating compared to backtracked or stalled RNAPIIs within the respective transcription window. Processivity indicates % of RNAPII reaching 1,000 bp.

(B) Fits of the model to the WT (left) and *spt4Δ* (right) NET-seq metagenes. Metagenes of the NET-seq data were constructed by taking the mean of the mean-normalized NET-seq reads of the first 1,000 nt of reads from the TSS. Metagenes of the simulated data were constructed by taking the mean of the set of mean-normalized best fitting single simulation for each gene. Data are binned with a bin size of 10 nt. Notably, the early simulation peak is in a region that is not expected

(legend continued on next page)

of Sua7 at promoters should also be higher than those in WT cells. To test this hypothesis, we performed spike-in normalized chromatin immunoprecipitation sequencing (ChIP-seq) for Sua7 in WT and *spt4Δ* cells in biological duplicates. Sua7 ChIP-seq data were reproducible between the replicates and mapped around the TSS (Figures 3A, S2A, and S2B; Doris et al., 2018). Visual inspection of individual genes and metagene plots revealed similar Sua7 occupancy in WT and *spt4Δ* cells. Differential enrichment analysis of the Sua7 signal indicated 94% of genes (3,148/3,269) showed no change in the absence of Spt4 (Figure 3B). Consequently, these data do not support a change at initiation frequency in *spt4Δ* cells, in line with the *in vitro* studies suggesting that the human counterpart of the Spt4/5 complex (DSIF) has no effect on the transcription initiation (Zhu et al., 2007).

This result was supported by mass spectrometry used to examine the composition of RNAPII in WT and *spt4Δ* cells (Figures 3C, 3D, and S2C). We detected factors involved with initiation, elongation, termination, and co-transcriptional pre-mRNA processing that did not change levels on RNAPII in the absence of Spt4 (Figures 3C and 3D; Table S2). However, levels of Set2 and five components of TFIIH (Rimel and Taatjes, 2018) were significantly depleted or enriched, respectively, on RNAPII in the absence of Spt4. We note that in mammalian cells, TFIIH is enriched on stalled transcription complexes under certain conditions (van der Weegen et al., 2020).

To distinguish between stalled/backtracked and elongation-competent RNAPII in *spt4Δ* cells, we used precision-run-on sequencing (PRO-seq) profiles (Booth et al., 2016) and compared them to our NET-seq profiles. PRO-seq allows the mapping of RNAPII that is competent to elongate during the metabolic labeling period, whereas NET-seq captures all forms of RNAPII, including elongated, backtracked, and stalled. Therefore, if RNAPII is captured by NET-seq, but not PRO-seq, it would indicate a non-elongating but still RNA-engaged RNAPII (for example, backtracked) at a given position. More stalled or backtracked RNAPII was observed in *spt4Δ* cells, particularly between ~90 nt and ~170 nt from the TSS (Figures 3E, 3F, S2D, and S2E). Thus, in *spt4Δ* cells, RNAPII transcribes with short-term pauses to around 90 nt from the TSS, whereas between 90 and 170 nt, more of the RNAPII is stalled or backtracked, leading to the decreased moving ratio in window 1, supporting an early elongation defect.

Rapid depletion of Spt4 leads to the accumulation of RNAPII around 170 nt into gene bodies

We monitored the effect of the real-time loss of the Spt4 protein from the nucleus by using the anchor-away system (AA) (Haruki et al., 2008) on the distribution of RNAPII. The AA allows the conditional removal of a target protein from the nucleus upon rapamycin addition. The efficient depletion of Spt4 was verified by immunofluorescence microscopy (Figure 3G) and quantified by the effect on growth rate (Figure S2F) and ChIP-qPCR (Figure S2G). NET-seq was performed in biological duplicate in Spt4 anchor away cells (Spt4-AA). DMSO-treated Spt4-FRB-GPF and rapamycin-treated No-FRB cells were included as controls. Spt4-AA NET-seq repeats and control experiments were reproducible (Figures S2H and S2I). Intriguingly, the real-time depletion of Spt4 had a small effect on the distribution of RNAPII across gene bodies but led to the most notable and significant changes in the RNAPII profile around 170 nt from the TSS (Figures 3H and 3I). This finding complements the *spt4Δ* NET-seq results and demonstrates that the change in the distribution of RNAPII in the absence of Spt4 first manifests itself around 170 nt downstream of the TSS.

The Spt4/5 complex travels with RNAPII

Next, we assessed where Spt4 and Spt5 associate with RNAPII to examine whether enrichment is related to transcription elongation at the 5′ end of genes, by using TEF-associated nascent elongating transcript sequencing (TEF-seq) to detect co-transcriptional and native interactions between Spt4/5 and RNAPII at a single-nucleotide resolution (Figure S3A; Fischl et al., 2017).

Spt4 or Spt5 were FLAG-tagged for immunoprecipitation of the factor-associated transcription complex. Spike-in-normalized Spt4 and Spt5 TEF-seq were performed in duplicate and gave reproducible results (Figures 4A and S3B). The Spt4 and Spt5 signals are similar over gene bodies, consistent with Spt4 and Spt5 forming a highly stable complex (Hartzog et al., 1998), and match the RNAPII (NET-seq) profile, supporting the engagement of these factors with RNAPII throughout transcription until 100 nt before the PAS when the signal drops to background (Figures 4B and S3C). These observations are consistent with structural and *in vitro* studies showing that Spt4/5 is recruited to the transcription elongation complex once 20-nt RNA has been synthesized, replacing transcription initiation factors (Bernecky et al., 2017; Grohmann et al., 2011; Rosen et al., 2020). The drop in the Spt5/4 signal at the 3′ end is likely to reflect a transition from elongation to termination, accompanying pausing of RNAPII (Kecman et al., 2018; Mischo and Proudfoot, 2013; Parua et al., 2018). Overall, the data indicate Spt4/5 join RNAPII right after initiation, travel with elongating RNAPII, and dissociate from RNAPII about 100 nt upstream of the PAS.

Additionally, to test whether Spt4/5 were differentially enriched for specific groups of genes, we plotted RNAPII, Spt4, and Spt5 signals as heatmaps based on the RNAPII occupancy level and performed a quantitative analysis of genome-wide Spt4 and Spt5 occupancies on RNAPII. The Spt4 and Spt5 levels were

to be reliably detected with the NET-seq protocol; therefore, the difference between the simulation and NET-seq data around this region is not necessarily contradictory.

(C) Metrics were obtained for each gene in WT and *spt4Δ,* and the two conditions were quantitatively compared. The significance of the changes was reported by calculating p values, and the magnitude of the changes was reported by calculating Cohen's d (Cohen, 1988). Cohen's d is computed by taking the mean difference between the WT and *spt4Δ* metric value divided by the standard deviation of the differences. The value of Cohen's d gives a measure of the effect size of the change such that the values between 0.2 and 0.5 indicate small changes, between 0.5 and 0.8 indicate medium changes, and >0.8 indicate large changes. Positive and negative values indicate a relative increase or decrease in the given metric, respectively. The red and blue boxplots indicate significant and marked increase and decrease, respectively, in the *spt4Δ* metric values compared to WT cells.

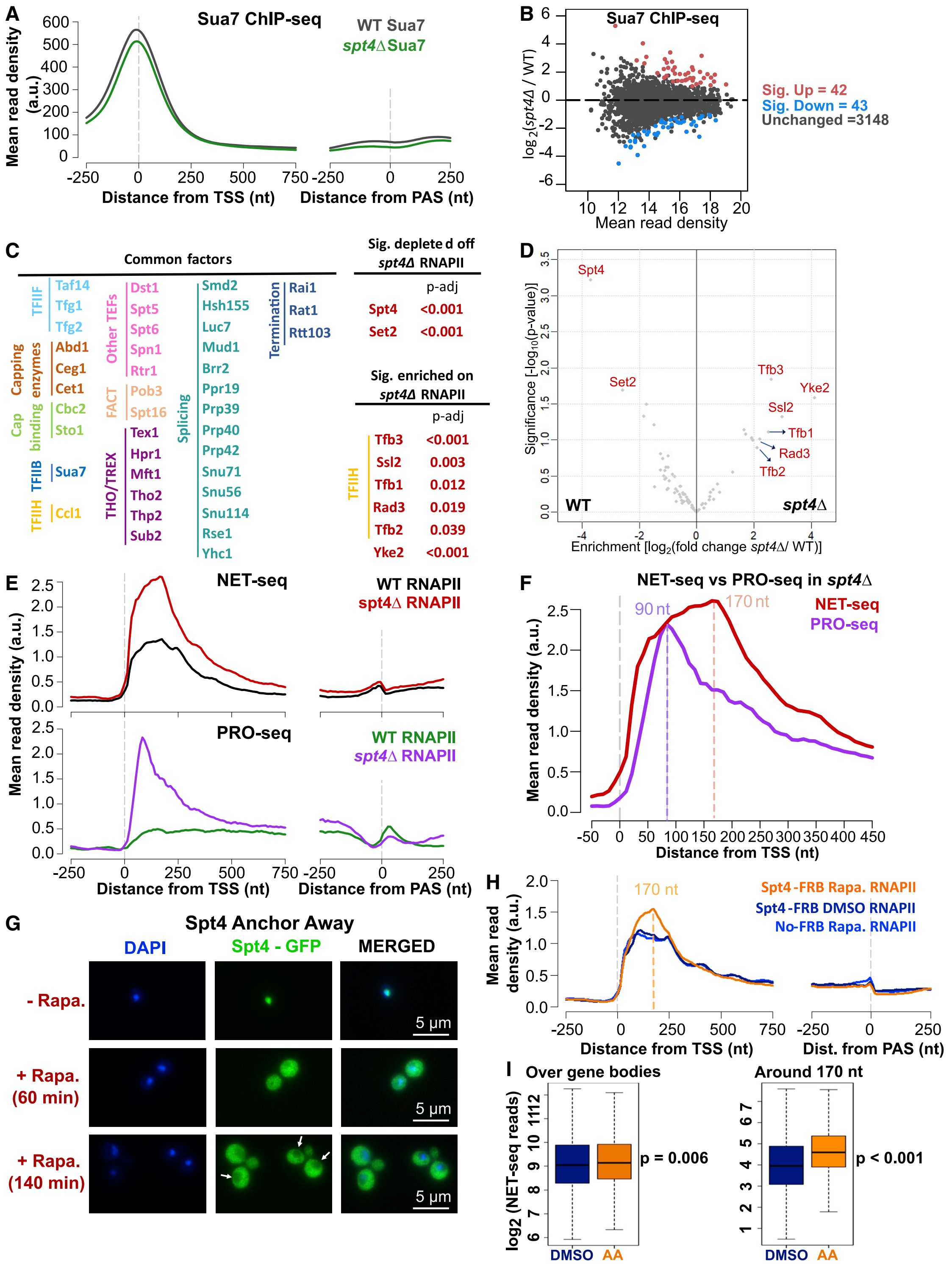

Figure 3. The primary defect in *spt4Δ* cells is early transcription elongation

(A) Metagene plots of Sua7 ChIP-seq reads in WT (black) and *spt4Δ* (green) aligned at the TSS or PAS for protein-coding genes (n = 3,233).

(B) Differential enrichment analysis of Sua7 in WT and *spt4Δ*. DEseq2 was applied to the read counts around the TSS (TSS-100 to TSS+100 nt) for the two replicates of each data. Significantly enriched and depleted genes are indicated in red and blue, respectively (p-adjusted < 0.05).

(C) WT and *spt4Δ* transcription complexes were purified using Rpb3 FLAG-tagged strains along with their no-tag controls, and their proteomics was analyzed following mass spectrometry. After the background removal, 68 and 78 factors were detected in WT and *spt4Δ* transcription complexes, respectively.

(legend continued on next page)

proportional to the RNAPII levels at most genes (>99%), and thus, Spt4/5 participates in transcription of nearly all mRNA genes (Figures S3D–S3F).

Spt4/5 oscillate on and off RNAPII based on nucleosome positions

The TEF-seq signals for Spt4/5 come from the native RNA attached to RNAPII associated with Spt4/5. To demonstrate the relative occupancies of Spt4 and Spt5 on RNAPII, we plotted the TEF-seq signal relative to the NET-seq signal. Interestingly, the association of Spt4/5 with RNAPII was not constant but instead periodically changed (Figure 4C). As their periodicity resembles the frequency of nucleosome phasing, we compared the NET-seq-normalized TEF-seq profiles with nucleosome positions, derived using micrococcal nuclease (MNase) digestion, followed by DNA sequencing (MNase-seq) in WT cells. MNase-seq was performed in biological triplicates, and nucleosome dyad positions were estimated. We observe a typical MNase-seq profile with nucleosome depleted regions (NDRs) at promoters, and nucleosomes are regularly arrayed in gene bodies relative to the TSSs (Baldi et al., 2020; Chereji et al., 2019; Figure 4D).

Next, we re-plotted the NET-seq-normalized TEF-seq profiles relative to the +1 nucleosome dyad to test if nucleosome positions correlate with the phasing patterns of Spt4/5. Remarkably, the oscillation pattern of the Spt4/5 on RNAPII was off-set with respect to nucleosome positions (Figure 4E). A more detailed analysis was done by plotting the metagene profiles around the nucleosome dyads (+1 to +4), separately (Figure 4F) or in 4 groups (n = 581 each) based on the location of the +1 nucleosome relative to the TSS (Figures S3G and S3H). At gene-body nucleosomes (+2 to +4), the Spt4 and Spt5 occupancies on RNAPII were significantly lower at the upstream face of the nucleosome dyads than those at the downstream face but not around the +1 nucleosome (Figure 4F). Similar enrichment/depletion based on Spt4 or Spt5 TEF-seq/NET-seq ratios are observed at the +2 nucleosome in groups 2 to 4, independent of levels of transcription, but not in group 1 where nucleosome phases are less well pronounced (Figures S3H and S3I).

Single-molecule experiments suggest long-lived Spt4/5-RNAPII interactions on naked DNA templates (Rosen et al., 2020). However, TEF-RNAPII associations are likely to be more dynamic in the context of chromatin as RNAPII-nucleosome conformations change while RNAPII is transcribing through nucleosomes. Indeed, Crickard et al. (2017) documented Spt4/5 stabilizing the RNAPII-nucleosome intermediate after RNAPII passes the dyad *in vitro*, and this is where we observe the higher levels of the Spt4/5 complex on RNAPII in cells. Together, these results support that Spt4/5 dynamically interacts with the transcription elongation complex during transcription and raises the question as to whether Spt4 and Spt5 have a direct and equivalent role in chromatin transcription.

Spt5 and Spt4 have distinct effects on transcription

As expected from factors in a complex, Spt4 and Spt5 show similar patterns of association with RNAPII across genes, including oscillations (Figure 4C). As Spt4 is important for the stability of Spt5 (Ding et al., 2010; Krasnopolsky et al., 2021), we also mapped the position of Spt5 during transcription in *spt4Δ* cells using TEF-seq (Figures 5A and S4A–S4C). The levels of Spt5 on RNAPII were reduced in the absence of Spt4 (Figure 5B), and oscillations of Spt5 on RNAPII were lost (Figures 5C and 5D), supporting a role for Spt4 in stabilizing/recruiting Spt5 to polymerase and in the oscillations of the complex on RNAPII as it transcribes through nucleosomes. Notably, in *spt4Δ* cells, Spt5 levels were proportional to the RNAPII levels at most genes (>99%), implying that Spt5 was not differentially recruited to genes in the absence of Spt4 (Figures S4D and S4E).

To distinguish whether Spt4 and Spt5 similarly affect transcription, we examined the distribution of RNAPII over the genes upon Spt5-AA. The efficient depletion of Spt5 was verified by immunofluorescence microscopy (Figure 5E) and quantified by the effect on growth rate (Figure S2F) and ChIP-qPCR (Figure S4F). NET-seq was performed in biological duplicate in Spt5-AA cells, including DMSO-treated Spt5-FRB-GPF and rapamycin-treated No-FRB cells as controls. Spt5-AA NET-seq repeats and control experiments were reproducible (Figures S4G and S4H). The loss of Spt5 resulted in a significant loss of NET-seq reads across the whole of the gene body (Figures 5F and 5G). These results demonstrated distinctly different NET-seq profiles upon Spt4 or Spt5 depletion, consistent with an additional, essential function for Spt5 (Shetty et al., 2017). As Spt5 affects transcription so dramatically, we focused only on Spt4 and asked whether Spt4 influences the organization of nucleosomes with the aim of explaining its effect on RNAPII distribution.

Transcription-related factors that were similarly enriched in both transcription complexes (log2(fold change spt4Δ/WT) < 2 and p-adj < 0.05) are listed under common factors (see Table S2 for the full list). Factors that were significantly depleted or enriched in the *spt4Δ* transcription complex (log2(fold change spt4Δ/WT) > 2 and p-adj < 0.05) are listed, and p-adjusted values are indicated.

(D) Volcano plot of the proteomics analysis of WT and *spt4Δ* transcription complexes. Factors that were significantly depleted off or enriched in the *spt4Δ* transcription complex (log2(fold change spt4Δ/WT) > 2 and p-adj < 0.05) are given in red.

(E) Metagene plots of NET-seq reads in WT (black) and *spt4Δ* (red) aligned at the TSS or PAS (top panel), and metagene plots of published PRO-seq reads in WT (green) and *spt4Δ* (purple) aligned at the TSS or PAS (bottom panel). PRO-seq data were taken from GEO:GSE76142 (Booth et al., 2016). n = 4,610.

(F) Metagene plots of *spt4Δ* NET-seq (red) and *spt4Δ* PRO-seq (purple) reads aligned at the TSS; the same data as in (E). Dashed lines indicate the highest PRO-seq (90 nt, purple) and NET-seq reads (170 nt, pink).

(G) Immunofluorescence (IF) images for Spt4-FRB samples at time points 0, 60, and 140 min after rapamycin addition. DAPI staining indicates nucleus, and GFP is expressed with Spt4 (Spt4-FRB-GFP).

(H) Metagene plots of NET-seq reads in DMSO control (navy), rapamycin-treated Spt4-FRB (orange), and No-FRB cells (blue) aligned at the TSS or PAS. Dashed line indicates the highest NET-seq read upon Spt4 anchor away (170 nt, orange).

(I) Boxplots of the NET-seq reads in DMSO control (DMSO; navy) and rapamycin-treated Spt4-FRB (AA; orange) cells on log_2 scale. Reads were counted for over gene bodies (TSS to PAS-250 nt) and at 170 ± 10 nt from the TSS for protein-coding genes after filtering for low read genes (see STAR Methods). n = 4560, p = 0.006 and p < 0.001, respectively; two-tailed, paired Student's t test.

CellPress
OPEN ACCESS

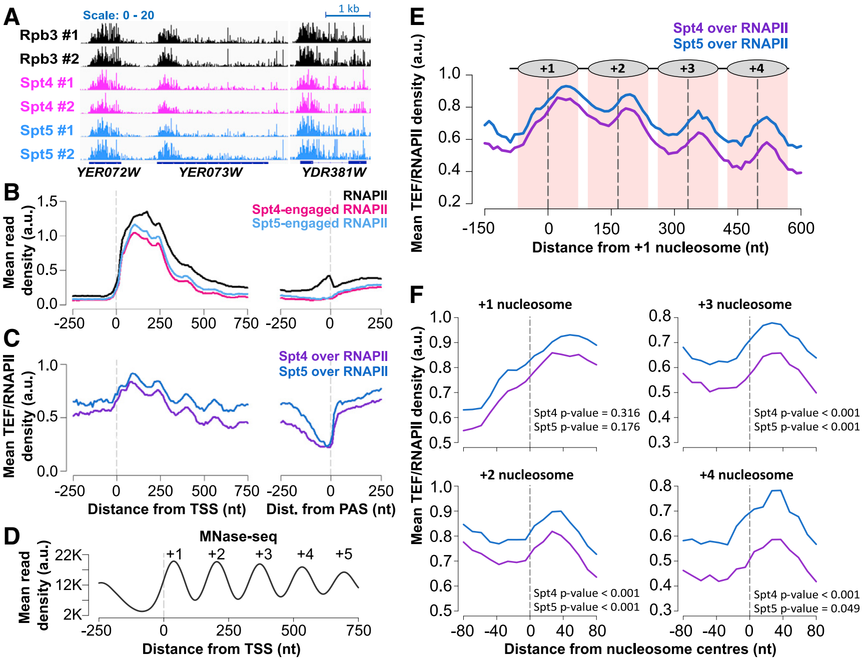

Figure 4. Spt4/5 travel with RNAPII and oscillate on and off RNAPII based on the nucleosome positions

(A) NET-seq (RNAPII) and TEF-seq (Spt4 and Spt5) reads of example genes transcribed from the positive strand, namely, *YER072W*, *YER073W*, and *YDR381W*, in two biological replicates. The dark-blue boxes indicate the transcribed region of the genes (from TSS to PAS), and the blue line indicates the intronic region in *YDR381W*.

(B) Metagene plots of NET-seq (RNAPII; black) and TEF-seq (Spt4; pink, Spt5; light blue) reads aligned at the TSS or PAS.

(C) Metagene plots of Spt4 over RNAPII (purple) and Spt5 over RNAPII (dark blue) data aligned at the TSS or PAS. Spt4 over RNAPII was plotted by dividing the Spt4-engaged RNAPII signal (TEF-seq) by the RNAPII signal (NET-seq). The same is applied to Spt5 TEF-seq data.

(D) Metagene plots of MNase-seq reads in WT cells aligned at the TSS. The dashed line in gray indicates the TSS.

(E) Metagene plots of Spt4 over RNAPII (purple) and Spt5 over RNAPII (dark blue) relative to the +1 nucleosome dyad. Dashed lines (black) through the peaks indicate the centers of the nucleosomes, and the nucleosomal DNA (±70 nt around the center) is highlighted in light pink. The position of nucleosomes is graphically shown above the metagene plot.

(F) Spt4 and Spt5 occupancies on RNAPII are shown around individual nucleosome dyads +1,+2,+3, and +4. The TEF/RNAPII values from upstream of the dyad (−60 to −10 nt from the dyad) and downstream of the dyad (+10 to +60 nt from the dyad) were compared for each gene. Significance of the change in the factor occupancies around the nucleosomes was tested by one-tailed (condition: upstream signal < downstream signal), paired Student's t test.

Spt4 influences nucleosome positioning

We have previously observed an oscillating pattern of Spt6 and Spt16 on RNAPII (Fischl et al., 2017), and interestingly, mutations in *spt6* and *spt16* have major effects on nucleosome positions (Doris et al., 2018; Feng et al., 2016). Therefore, next, we asked whether Spt4 has an effect on nucleosome arrangement using MNase-seq in *spt4Δ* cells (Figures S5A and S5B). Three replicates of MNase-seq in *spt4Δ* cells produced reproducible digestion patterns (Figure S5C). A genome-wide analysis was performed for protein-coding genes (PCGs) longer than 600 nt and with well-defined nucleosome peaks. Interestingly, in *spt4Δ* cells, although MNase digestion resulted in well-defined peaks across the genome, nucleosomes were shifted toward the 3′ end of the genes compared to WT cells (Figures 6A and 6B). The differences between the nucleosome positions were more apparent at downstream nucleosomes (Figures 6C and S5D). A more detailed analysis was performed by calculating the position of the −1, +1, +2, +3, and +4 nucleosomes relative to the TSS in the 3 replicates of the MNase-seq data. There was no difference in the median positions of the −1 and +1 nucleosomes or NDR length (defined as the distance between the dyads of the −1 and +1 nucleosome) in WT and *spt4Δ* cells (Figures 6C, S5D, and S5E). In contrast, the positions of the +2, +3, and +4 nucleosome in *spt4Δ* cells were progressively shifted 3′ compared to WT cells (Figures 6C and S5D), suggesting increased nucleosome spacing (defined as the distance between the dyads of adjacent nucleosomes) in *spt4Δ* cells. Indeed, nucleosome spacing between nucleosome pairs (+1 to +2, +2 to +3, and +3 to +4) in *spt4Δ* was larger than that in WT cells (Figure S5E). Overall, the data support a role for Spt4 in positioning the gene-body nucleosomes from the +2 nucleosome but no role in positioning the −1 and +1 nucleosomes. This finding suggests that Spt4 affects nucleosome positioning during transcription elongation. Importantly, as nucleosome positioning involves numerous factors and redundant mechanisms, we cannot rule out the possibility of Spt4 indirectly affecting the nucleosome positioning.

Close nucleosome spacing at highly transcribed genes is dependent on Spt4

If Spt4 has a co-transcriptional effect on nucleosome positioning, nucleosome spacing should be affected by the deletion of Spt4 to a greater extent in highly active genes than in less active genes. To test this hypothesis, we investigated the correlation between nucleosome spacing and RNAPII densities (Figures 6D and 6E). PCGs were split into 8 groups based on their RNAPII density assessed by NET-seq reads. For each group, the median RNAPII density and the median nucleosome spacing between the +1 and +2, as well as between the +2 and +3 nucleosomes in WT and *spt4Δ*, were plotted together. In WT cells, nucleosome spacing was shorter in highly expressed genes, and it progressively increased for the genes having lower expression levels. In *spt4Δ* cells, the distance between the nucleosomes was less variable and larger than that of WT in all groups. In other words, there was an overall increase in nucleosome spacing in *spt4Δ* cells compared to that in WT cells, and the increase was larger for highly expressed genes. This analysis shows that close nucleosome

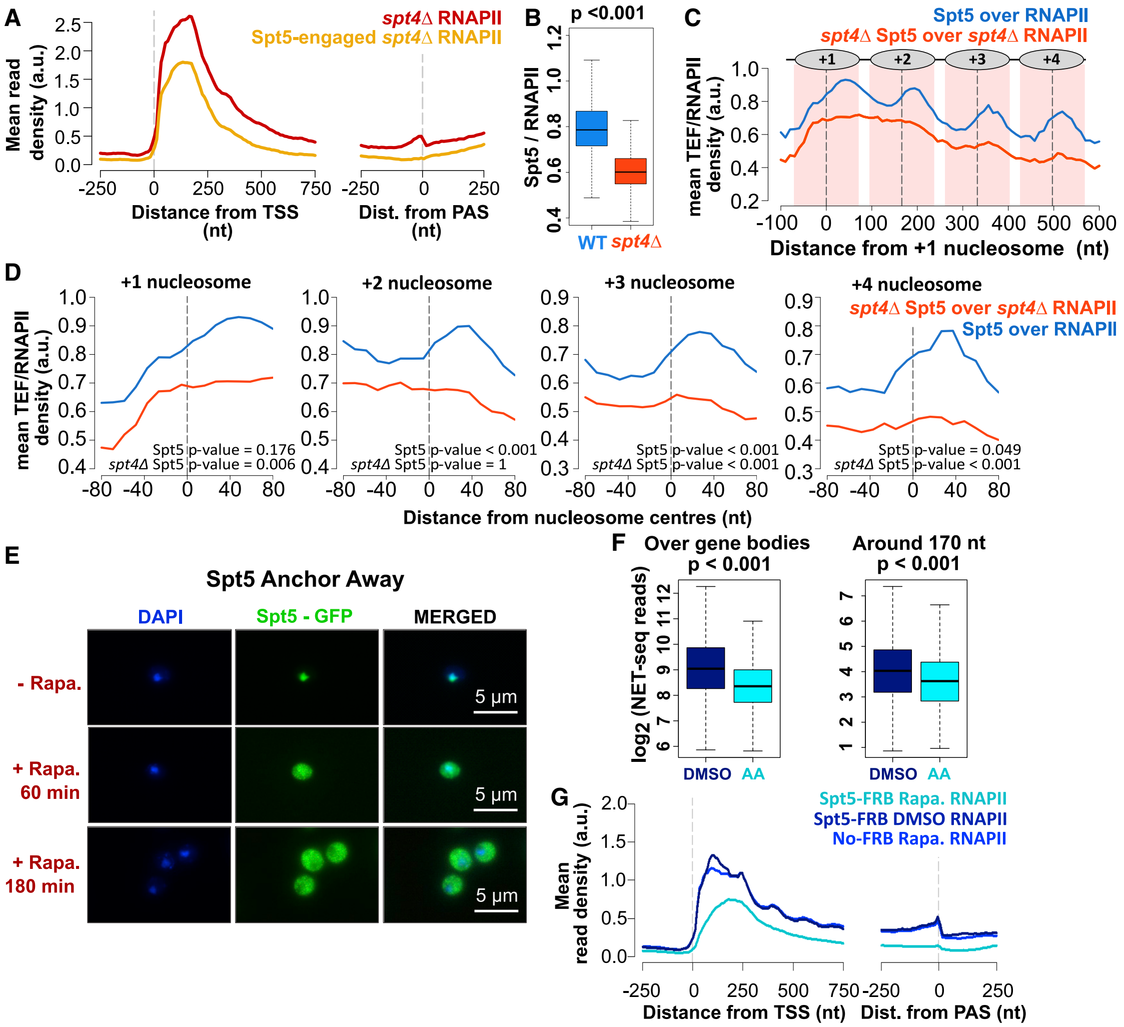

Figure 5. Spt5 and Spt4 have distinct effects on transcription

(A) Metagene plots of *spt4Δ* NET-seq (RNAPII; red) and *spt4Δ* Spt5 TEF-seq (yellow) reads aligned at the TSS or PAS.

(B) Boxplots of Spt5 over RNAPII (blue) and *spt4Δ* Spt5 over *spt4Δ* RNAPII (yellow) ratios for the protein-coding genes. The medians of the ratios (0.79 and 0.60, respectively) were calculated by taking the reads from gene bodies (TSS to PAS-250 nt) of Spt5 TEF-seq and dividing by the reads from gene bodies of NET-seq both in WT and *spt4Δ* (p value < 0.001; Student's t test, paired, two-tailed). We note the superior sensitivity of TEF-seq compared to mass spectrometry for detecting the levels of Spt5 on RNAPII.

(C) Metagene plots of Spt5 over RNAPII (blue) and *spt4Δ* Spt5 over *spt4Δ* RNAPII (orange) relative to the +1 nucleosome dyad. Plotted as described in Figure 4E.

(D) Spt5 occupancies on RNAPII in WT or *spt4Δ* cells are shown around individual nucleosome dyads +1, +2, +3, and +4. Plotted and tested as described in Figure 4F.

(E) IF images for Spt5-FRB samples at time points 0, 60, and 180 min after rapamycin addition. DAPI staining indicates nucleus, and GFP is expressed with Spt5 (Spt5-FRB-GFP).

(F) Boxplots of the NET-seq reads in DMSO control (DMSO; navy) and rapamycin-treated Spt5-FRB (AA; cyan) cells on a log_2 scale. Reads were counted for over gene bodies (TSS to PAS-250 nt) and around 170 bp as in Figure 3I. n = 4,417; p values < 0.001; two-tailed, paired Student's t test.

(G) Metagene plots of NET-seq reads in DMSO control (navy), rapamycin-treated Spt5-FRB (cyan), and No-FRB cells (blue) aligned at the TSS or PAS.

spacing observed in highly transcribed genes was lost in the absence of Spt4.

The accumulation of RNAPII in the absence of Spt4 is associated with the position of the +2 nucleosome

The dynamic interaction of Spt4 with RNAPII based on nucleosome positions and the effect of Spt4 on gene-body nucleosome positions point to a role for Spt4 in chromatin transcription. Furthermore, the accumulation of RNAPII around 170 nt from the TSS in the absence of Spt4 supports a transcriptional barrier around this point. As *in vitro* studies have shown that the Spt4/5 complex does not help RNAPII progress over non-nucleosomal transcription barriers (Xu et al., 2020) but does aid RNAPII movement over nucleosomal barriers

CellPress
OPEN ACCESS

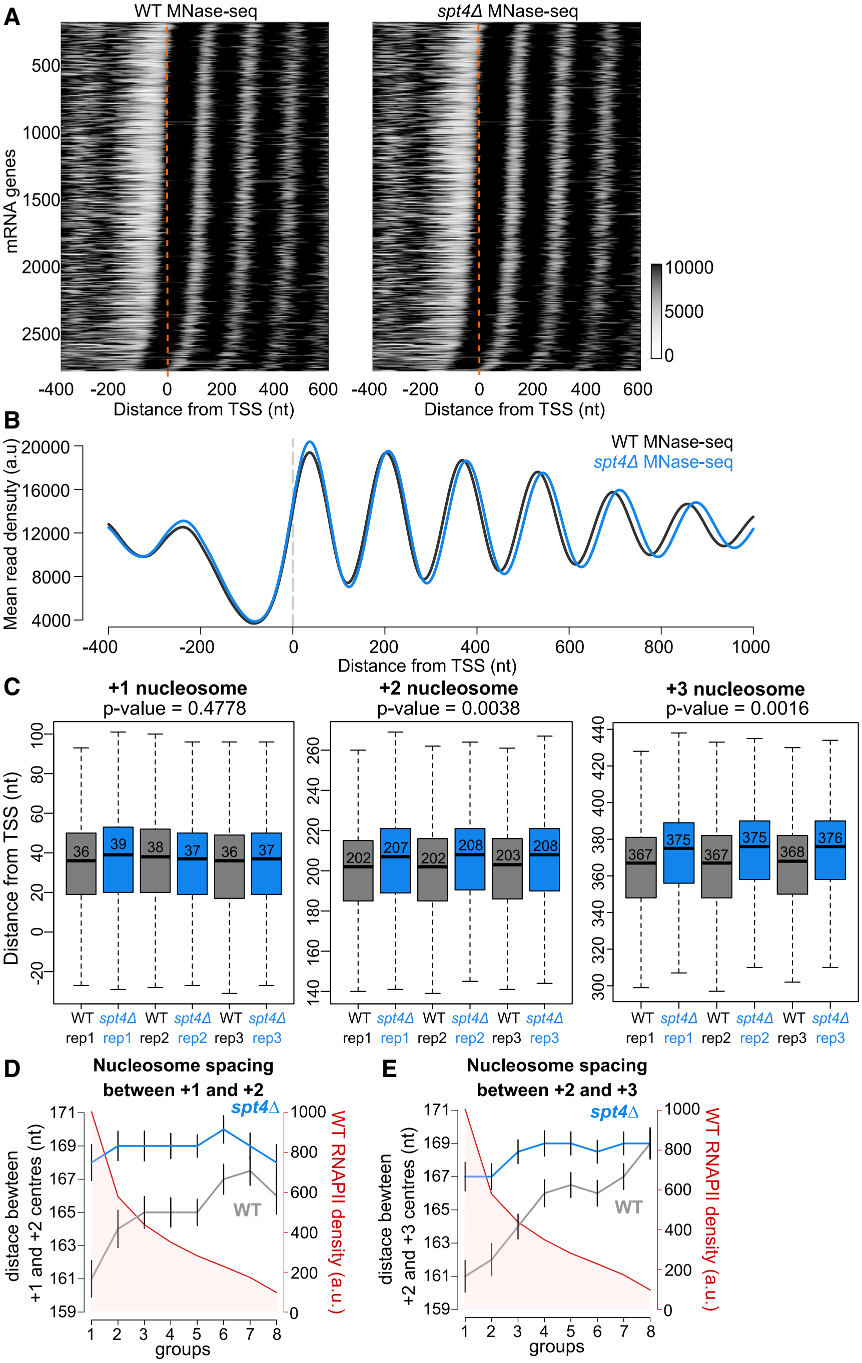

Figure 6. Spt4 influences nucleosome positioning

(A) Heatmaps of MNase-seq reads in WT (left) and *spt4Δ* (right). PCGs ordered based on the position of +1 nucleosome in WT. Dashed line in orange indicates the TSS.

(B) Metagene plots of MNase-seq reads in WT (black) and *spt4Δ* cells (blue) aligned at the TSS. The dashed line in gray indicates the TSS.

(C) Box plots of the distance of the +1, +2, and +3 nucleosomes from the TSS in three biological replicates of WT (black) and *spt4Δ* cells (blue). Numbers in the boxes indicate the median position of the given nucleosome. p values were calculated by comparing the median position of the +1, +2, or +3 nucleosomes under WT and *spt4Δ* conditions obtained from each replicate (Student's t test, paired, two sided).

(D) Protein-coding genes were split into 8 groups based on WT NET-seq reads in the first 500 nt from the TSS (red, y axis). The median NET-seq reads and the median nucleosome spacing between +1 and +2 nucleosomes in *spt4Δ* (blue) and WT (gray) were plotted for each group. The black bars around nucleosome spacing data points indicate one standard deviation.

(E) The median nucleosome spacing between +2 and +3 nucleosomes in *spt4Δ* (blue) and WT (gray) was plotted for each group as in (D).

(Ehara et al., 2019; Farnung et al., 2021), we sought *in vivo* evidence for these findings by investigating the change in the distribution on RNAPII relative to nucleosome positions in the absence of Spt4.

Mapping the RNAPII density from normalized NET-seq reads to the position of the nucleosome dyads (+1 to +4) revealed the position of RNAPII accumulation at the upstream face of the +2 nucleosome in *spt4Δ* cells and to a lesser extent at the +3 and +4 nucleosomes (Figures 7A, 7B, and S6A). Importantly, this was also observed in Spt4-AA cells at the +2 nucleosome (Figures 7C and S6A), verifying the effect of the loss of Spt4 on the RNAPII distribution relative to nucleosomes in two different backgrounds. For both *spt4Δ* and Spt4-AA cells, accumulation at the +2 nucleosome is independent of RNAPII occupancy over genes (Figures S6B–S6E) and evident only when the shape of the distribution is considered (Figures S6F and S6G). Accumulation of RNAPII at the +2 nucleosome is likely to be independent of initiation, as it is evident at 43 genes that show reduced promoter-associated Sua7 in *spt4Δ* cells (Figure S6H). These results suggest that the main function of Spt4 is preventing RNAPII accumulation at the upstream face of nucleosomes, most specifically at the +2 nucleosome.

As our modeling and experimental data pointed to increased stalling or backtracking during early elongation, we compared the NET-seq profile from *spt4Δ* cells and cells lacking elongation factors Dst1 (TFIIS) and Paf1. As Paf1 associates with RNAPII between 200 and 400 nt from the TSS (Fischl et al., 2017), it is not expected to have a major effort on early elongation. Indeed, low levels of RNAPII accumulate on the downstream face of the +2 nucleosome in *paf1Δ* cells (Figures S7A and S7B). Dst1 is a TEF that helps rescue backtracked RNAPII by triggering the cleavage activity of RNAPII (Zatreanu et al., 2019). Furthermore, the loss of

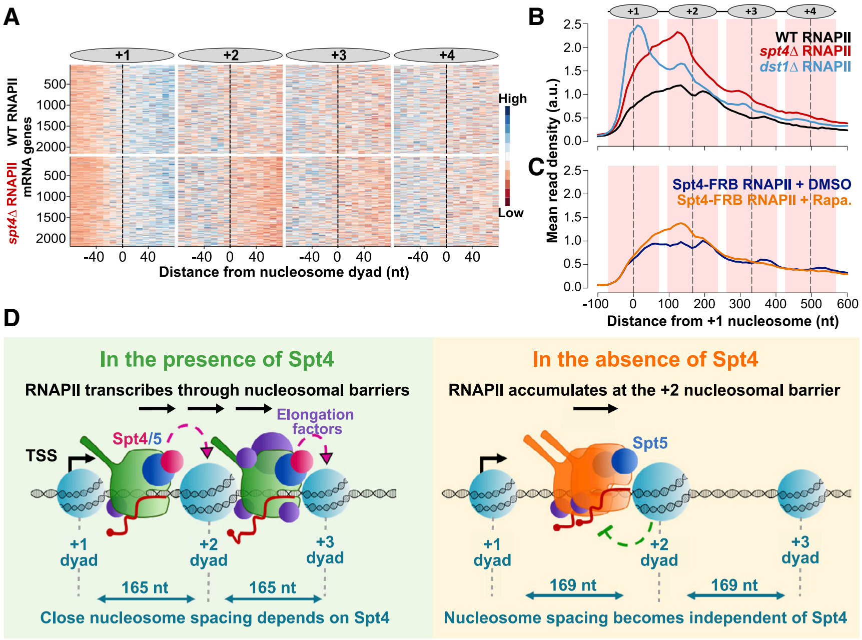

Figure 7. The accumulation of RNAPII in the absence of Spt4 is associated with the position of the +2 nucleosome

(A) Heatmaps of WT (top) and *spt4Δ* (bottom) NET-seq profiles around the +1, +2, +3, and +4 nucleosomes. Each row indicates a PCG (n = 2,212). The RNAPII signal is shown in 10-nt bins around the indicated nucleosome dyads (±80 nt from the dyad; x axis). The NET-seq reads were normalized to the mean and standard deviation of each gene to indicate the shape of the distribution of RNAPII regardless of the expression level differences between the genes.

(B) Metagene plots of WT (black), *spt4Δ* (red), and *dst1Δ* (blue) NET-seq profiles relative to the +1 nucleosome dyad. Dashed lines (black) through the peaks indicate the centers of the nucleosomes, and the nucleosomal DNA (±70 nt around the center) is highlighted in light pink. The position of nucleosomes is graphically shown above the metagene plot. The NET-seq reads were normalized to spike-ins.

(C) Metagene profiles of DMSO-treated (navy) and rapamycin-treated (orange) Spt4-FRB NET-seq profiles. Plotted as described in (B).

(D) Model for Spt4 function.

Dst1 is reported to lead to RNAPII accumulation around the nucleosome dyads (Churchman and Weissman, 2011). Interestingly, the RNAPII profiles in *spt4Δ* and *dst1Δ* are quite distinct, with Dst1 function focused on the dyad region of the +1 nucleosome (Figures 7B, S6G, S7C, and S7D). This finding confirms that the accumulation of reads around the +1 nucleosome can be detected using NET-seq and supports a specific function for Spt4 in elongation at the +2 and, to a lesser extent, at +3 and +4 nucleosomes. Taken together, we propose that the *in vivo* function of Spt4 involves helping RNAPII pass nucleosomal barriers downstream of the +1 nucleosome, especially at the +2 nucleosome (Figure 7D).

DISCUSSION

Although structural and *in vitro* studies implicated Spt4/5 in RNAPII movement through nucleosomes, their precise role in transcription in the cell is poorly defined. Here, we reveal that Spt4/5 associates with RNAPII early in transcription and travels with elongating RNAPII over the gene bodies. As RNAPII transcribes over nucleosomes, the association of Spt4/5 with RNAPII oscillates and is higher at the downstream face of the dyad. Although Spt4 and Spt5 show similar distributions on RNAPII, Spt4 and Spt5 have different effects on RNAPII density over genes. Spt4 leads to an accumulation of RNAPII at the 5′ end of genes, particularly at the upstream face of the +2 nucleosome, and to a lesser extent at the upstream face of the +3 and +4 nucleosomes. Interestingly, the accumulation of RNAPII on nucleosomes occurs at positions where levels of Spt4 are lowest. Finally, we show that in the absence of Spt4, the positions of the gene-body nucleosomes (+2 and beyond) are shifted downstream. Together, our data point to a primary role for Spt4 in regulating the movement of RNAPII through the +2 nucleosomal barrier.

Could Spt4 use the same mechanism to influence the nucleosome-related oscillations on RNAPII, the efficient movement of RNAPII through the +2 nucleosomal barrier, and nucleosome spacing? We considered the following two possibilities: an interaction with histones and/or with the nucleosomal DNA.

Like the Spt4/5 complex, the histone chaperones Spt6 and Spt16 also oscillate, out of phase, on and off RNAPII, reflecting their dynamic interactions with different histones during transcription (Fischl et al., 2017). This raises the possibility of distinct affinities by these different TEFs for specific conformations of histones with elongating RNAPII, leading to the oscillations. Like histone chaperones, Spt5 bears an acidic domain that is predicted to interact with H2A/H2B during transcription (Ehara et al., 2019; Farnung et al., 2021). Spt4 does not have charged domains, but the affinity of Spt4 for nucleosomes could change indirectly through Spt5. The second possibility is binding of the Spt4/5 complex to nucleosomal DNA as it peels off from the nucleosome while RNAPII is moving forward. Indeed, Spt5 interacts with free DNA *in vitro*, suggesting that such dynamics between the Spt4/5 and DNA are also possible (Crickard et al., 2016). Either through an interaction with histones or nucleosomal DNA (or both), our model supports a function for Spt4 facilitating RNAPII movement on the nucleosomal barriers and aligns well with an *in vitro* model suggesting that together with FACT or Chd1, Spt4/5 contributes to effective RNAPII transcription through a nucleosome (Farnung et al., 2021).

This function of Spt4 would also explain why RNAPII accumulates at the upstream face of the nucleosomes. As *spt4Δ* cells are viable, RNAPII appears to pass nucleosomal barriers by redundant mechanisms, but they might be less effective at the +2 nucleosome, which was also recently recognized as an important barrier in stress (Badjatia et al., 2021). Additionally, dynamic changes in the composition of the transcription elongation complex as RNAPII transcribes along the genes could explain

why the most notable effect of the loss of Spt4 is at the +2 nucleosome. Factors such as the Paf1 complex (Paf1C) are recruited to RNAPII around the +2 nucleosome, and its level on RNAPII progressively increases toward the 3′ end of genes (Fischl et al., 2017). In the absence of Paf1, RNAPII accumulates at the downstream face of the +2 nucleosome. Paf1C is a TEF complex generally associated with productive elongation as it takes part in co-transcriptional histone PTMs (Van Oss et al., 2017) and increases the processivity of RNAPII *in vitro* (Vos et al., 2020). Therefore, the movement of RNAPII through the upstream face of the +2 nucleosome might rely more on the function of Spt4. Around the +3 and +4 nucleosomes, Spt4 still contributes to transcription, possibly providing allosteric interactions. This could also explain the synthetic lethality in the double mutants of *spt4* and genes encoding the five Paf1C components (Squazzo et al., 2002). Alternatively, the reason why Spt4 is most crucial for passing the +2 nucleosome might be related to specific histone post-translational modifications (PTMs). The role of histone PTMs in overcoming nucleosome barriers remains unknown, and future studies will be needed to investigate this role.

The negative correlation between nucleosome spacing and the RNAPII density on genes observed here and by others (Baldi et al., 2018; Ocampo et al., 2016) could result from high levels of transcription causing either removal or re-positioning of nucleosomes to allow RNAPII passage (Singh and Mueller-Planitz, 2021). This would, in turn, lead to a delay in the restoration of normal spacing, which is an energy-requiring process involving remodelers such as Isw1 and Chd1 (Gkikopoulos et al., 2011; Kent et al., 2001; Morillon et al., 2003; Ocampo et al., 2019). Here, our model would also explain the increased nucleosome spacing observed in the absence of Spt4. If Spt4 helps RNAPII pass nucleosomal barriers, inefficient removal or re-positioning of nucleosomes would eliminate the need for restoration of nucleosome positioning that would also explain the observations suggesting opposing roles for Isw1 and Spt4 in transcription through chromatin (Morillon et al., 2003).

Finally, we considered a role for Spt4 in transcription itself. Our mathematical model predicts and others report that in *SPT4* mutants, RNAPII shows an elongation defect and is less processive (Booth et al., 2016; Hartzog and Fu, 2013; Mason and Struhl, 2005). This must be balanced by a reduction in transcript turnover rates (Brown et al., 2018), as overall levels of transcripts do not change in *spt4Δ* cells (Booth et al., 2016). The increased NET-seq signal would also be consistent with an elongation defect in *spt4Δ* cells. Is an elongation defect linked to the nucleosome spacing defect, which is similar to a pattern that is normally observed in lowly expressed genes or upon RNAPII depletion (Singh and Mueller-Planitz, 2021; Weiner et al., 2010), or to accumulation of RNAPII on nucleosomes? Work with other mutants suggests no simple relationship between the accumulation of RNAPII upstream of nucleosomes, reduced RNAPII processivity, and increased nucleosome spacing. For example, *hpr1Δ* mutants have less processive RNAPII (Mason and Struhl, 2005) and *dst1Δ* mutants lead to RNAPII accumulation around the +1, and to a lesser extent the +2, nucleosome dyad, but there is no change in nucleosome positioning in these mutants compared to WT cells (Chávez et al., 2001; Gutiérrez et al., 2017). This would support a function for Spt4 in maintaining efficient transcription elongation by facilitating the movement of RNAPII through nucleosomal barriers.

In conclusion, our results corroborate structural and *in vitro* studies that implicate Spt4 as an important factor for efficient RNAPII movement through nucleosomal barriers. Importantly, this study further reveals that the contribution of Spt4 to transcription is not uniform across the transcription unit but more substantial in early elongation, particularly at the +2 nucleosomal barrier. We expect that future studies will address if this function of Spt4 is conserved in mammals and if the mammalian counterpart of Spt4 has a function in the RNAPII pausing observed in early transcription.

LIMITATIONS OF STUDY

We revealed that Spt4 promotes RNAPII movement through nucleosomal barriers *in vivo*, and the affinity of Spt4/5 with RNAPII is lower at the upstream face of the nucleosomal DNA and higher at the downstream, implying that Spt4/5 dynamically interact with RNAPII as it transcribes through nucleosomes. As TEF-seq was performed in bulk cultures, we cannot conclude if oscillations of Spt4/5 are due to the factors fully coming on and off RNAPII, changes in the relative affinities of the factors with RNAPII, or a systematic bias in the TEF-seq assay. This question could be addressed in the future by using single-molecule approaches including RNAPII and Spt4/5 combined with nucleosomal DNA templates.

STAR★METHODS

Detailed methods are provided in the online version of this paper and include the following:

- KEY RESOURCES TABLE
- RESOURCE AVAILABILITY
  - Lead contact
  - Materials availability
  - Data and code availability
- EXPERIMENTAL MODEL AND SUBJECT DETAILS
  - Yeast strains and culturing
- METHOD DETAILS
  - NET-seq/TEF-seq
  - ChIP-seq
  - Proteomics Sample Collection and Analysis
  - Anchor Away
  - MNase-seq
  - Mathematical Modeling
- QUANTIFICATION AND STATISTICAL ANALYSIS
  - Statistical Analysis
- ADDITIONAL RESOURCES

SUPPLEMENTAL INFORMATION

Supplemental information can be found online at https://doi.org/10.1016/j.celrep.2021.109755.

ACKNOWLEDGMENTS

We thank Frank Holstege for providing the *S.cerevisiae* Anchor Away strains, Lidia Vasilieva for Rpb9 FLAG-tagged *S.pombe* strain, Sabrina Liberatori and Shabaz Mohammed for proteomics, and Micron Oxford for imaging support. This work was supported by a Cancer Research UK (CRUK) grant (C5255/A23225), a CRUK Oxford Centre Prize DPhil Studentship to Ü.U., an EPSRC studentship to T.B. (EP/F500394/10), a Royal Society University Research Fellowship to A.A. (UF120327), and BBSRC grants to J.M. (BB/S009035/1 for A.A. and BB/P00296X/1 for H.F).

AUTHOR CONTRIBUTIONS

Ü.U. and J.M. designed the experiments. Ü.U. performed experiments and analyzed datasets. T.B. and A.A. performed and analyzed mathematical modeling. H.F. set up the initial framework and analysis of TEF-seq and performed part of the MNase-seq analysis. Ü.U. and J.M. wrote the manuscript, with input from all authors.

DECLARATION OF INTERESTS

J.M. is a shareholder in and acts as an advisor to Oxford Biodynamics plc and Sibelius Natural Products Ltd. J.M. is a shareholder in Chronos Therapeutics Ltd. None of these companies have an interest in the work described here. None of the remaining authors has a conflict of interest to declare.

Received: March 19, 2021
Revised: June 18, 2021
Accepted: September 2, 2021
Published: September 28, 2021

REFERENCES

Almer, A., Rudolph, H., Hinnen, A., and Hörz, W. (1986). Removal of positioned nucleosomes from the yeast PHO5 promoter upon PHO5 induction releases additional upstream activating DNA elements. EMBO J. *5*, 2689–2696.

Azofeifa, J.G., and Dowell, R.D. (2017). A generative model for the behavior of RNA polymerase. Bioinformatics *33*, 227–234.

Badjatia, N., Rossi, M.J., Bataille, A.R., Mittal, C., Lai, W.K.M., and Pugh, B.F. (2021). Acute stress drives global repression through two independent RNA polymerase II stalling events in Saccharomyces. Cell Rep. *34*, 108640.

Baldi, S., Krebs, S., Blum, H., and Becker, P.B. (2018). Genome-wide measurement of local nucleosome array regularity and spacing by nanopore sequencing. Nat. Struct. Mol. Biol. *25*, 894–901.

Baldi, S., Korber, P., and Becker, P.B. (2020). Beads on a string-nucleosome array arrangements and folding of the chromatin fiber. Nat. Struct. Mol. Biol. *27*, 109–118.

Bernecky, C., Plitzko, J.M., and Cramer, P. (2017). Structure of a transcribing RNA polymerase II-DSIF complex reveals a multidentate DNA-RNA clamp. Nat. Struct. Mol. Biol. *24*, 809–815.

Blankenberg, D., Gordon, A., Von Kuster, G., Coraor, N., Taylor, J., and Nekrutenko, A.; Galaxy Team (2010). Manipulation of FASTQ data with Galaxy. Bioinformatics *26*, 1783–1785.

Booth, G.T., Wang, I.X., Cheung, V.G., and Lis, J.T. (2016). Divergence of a conserved elongation factor and transcription regulation in budding and fission yeast. Genome Res. *26*, 799–811.

Brown, T., Howe, F.S., Murray, S.C., Wouters, M., Lorenz, P., Seward, E., Rata, S., Angel, A., and Mellor, J. (2018). Antisense transcription-dependent chromatin signature modulates sense transcript dynamics. Mol. Syst. Biol. *14*, e8007.

Chávez, S., García-Rubio, M., Prado, F., and Aguilera, A. (2001). Hpr1 is preferentially required for transcription of either long or G+C-rich DNA sequences in Saccharomyces cerevisiae. Mol. Cell. Biol. *21*, 7054–7064.

Chen, K., Xi, Y., Pan, X., Li, Z., Kaestner, K., Tyler, J., Dent, S., He, X., and Li, W. (2013). DANPOS: dynamic analysis of nucleosome position and occupancy by sequencing. Genome Res. *23*, 341–351.

Chereji, R.V., Bryson, T.D., and Henikoff, S. (2019). Quantitative MNase-seq accurately maps nucleosome occupancy levels. Genome Biol. *20*, 198.

Churchman, L.S., and Weissman, J.S. (2011). Nascent transcript sequencing visualizes transcription at nucleotide resolution. Nature *469*, 368–373.

Clapier, C.R., Iwasa, J., Cairns, B.R., and Peterson, C.L. (2017). Mechanisms of action and regulation of ATP-dependent chromatin-remodelling complexes. Nat. Rev. Mol. Cell Biol. *18*, 407–422.

Cohen, J. (1988). Statistical Power Analysis for the Behavioral Sciences (Routledge Academic).

Crickard, J.B., Fu, J., and Reese, J.C. (2016). Biochemical analysis of yeast suppressor of Ty 4/5 (Spt4/5) reveals the importance of nucleic acid interactions in the prevention of RNA polymerase II arrest. J. Biol. Chem. *291*, 9853–9870.

Crickard, J.B., Lee, J., Lee, T., and Reese, J.C. (2017). The elongation factor Spt4 / 5 regulates RNA polymerase II transcription through the nucleosome. Nucleic Acids Res. *45*, 6362–6374.

Cucinotta, C.E., Dell, R.H., Braceros, K.C., and Tsukiyama, T. (2021). RSC primes the quiescent genome for hypertranscription upon cell-cycle re-entry. eLife *10*, 2021.01.28.428695.

Decker, T.-M. (2021). Mechanisms of Transcription Elongation Factor DSIF (Spt4-Spt5). J. Mol. Biol. *433*, 166657.

Ding, B., LeJeune, D., and Li, S. (2010). The C-terminal repeat domain of Spt5 plays an important role in suppression of Rad26-independent transcription coupled repair. J. Biol. Chem. *285*, 5317–5326.

Doris, S.M., Chuang, J., Viktorovskaya, O., Murawska, M., Spatt, D., Churchman, L.S., and Winston, F. (2018). Spt6 Is Required for the Fidelity of Promoter Selection. Mol. Cell *72*, 687–699.e6.

Ehara, H., Kujirai, T., Fujino, Y., Shirouzu, M., Kurumizaka, H., and Sekine, S.I. (2019). Structural insight into nucleosome transcription by RNA polymerase II with elongation factors. Science *363*, 744–747.

Farnung, L., Vos, S.M., and Cramer, P. (2018). Structure of transcribing RNA polymerase II-nucleosome complex. Nat. Commun. *9*, 5432.

Farnung, L., Ochmann, M., Engeholm, M., and Cramer, P. (2021). Structural basis of nucleosome transcription mediated by Chd1 and FACT. Nat. Struct. Mol. Biol. *28*, 382–387.

Feng, J., Gan, H., Eaton, M.L., Zhou, H., Li, S., Belsky, J.A., MacAlpine, D.M., Zhang, Z., and Li, Q. (2016). Noncoding Transcription Is a Driving Force for Nucleosome Instability in spt16 Mutant Cells. Mol. Cell. Biol. *36*, 1856–1867.

Fischer, J., Song, Y.S., Yosef, N., di Iulio, J., Churchman, L.S., and Choder, M. (2020). The yeast exoribonuclease Xrn1 and associated factors modulate RNA polymerase II processivity in 5′ and 3′ gene regions. J. Biol. Chem. *295*, 11435–11454.

Fischl, H., Howe, F.S., Furger, A., and Mellor, J. (2017). Paf1 Has Distinct Roles in Transcription Elongation and Differential Transcript Fate. Mol. Cell *65*, 685–698.e8.

Gentleman, R.C., Carey, V.J., Bates, D.M., Bolstad, B., Dettling, M., Dudoit, S., Ellis, B., Gautier, L., Ge, Y., Gentry, J., et al. (2004). Bioconductor: open software development for computational biology and bioinformatics. Genome Biol. *5*, R80.

Gkikopoulos, T., Schofield, P., Singh, V., Pinskaya, M., Mellor, J., Smolle, M., Workman, J.L., Barton, G.J., and Owen-Hughes, T. (2011). A role for Snf2-related nucleosome-spacing enzymes in genome-wide nucleosome organization. Science *333*, 1758–1760.

Grohmann, D., Nagy, J., Chakraborty, A., Klose, D., Fielden, D., Ebright, R.H., Michaelis, J., and Werner, F. (2011). The initiation factor TFE and the elongation factor Spt4/5 compete for the RNAP clamp during transcription initiation and elongation. Mol. Cell *43*, 263–274.

CellPress
OPEN ACCESS

Gurova, K., Chang, H.W., Valieva, M.E., Sandlesh, P., and Studitsky, V.M. (2018). Structure and function of the histone chaperone FACT—Resolving FACTual issues. Biochim. Biophys. Acta. Gene Regul. Mech. *1861*, 892–904.

Gutiérrez, G., Millán-Zambrano, G., Medina, D.A., Jordán-Pla, A., Pérez-Ortín, J.E., Peñate, X., and Chávez, S. (2017). Subtracting the sequence bias from partially digested MNase-seq data reveals a general contribution of TFIIS to nucleosome positioning. Epigenetics Chromatin *10*, 58.

Hartzog, G.A., and Fu, J. (2013). The Spt4-Spt5 complex: a multi-faceted regulator of transcription elongation. Biochim. Biophys. Acta *1829*, 105–115.

Hartzog, G.A., Wada, T., Handa, H., and Winston, F. (1998). Evidence that Spt4, Spt5, and Spt6 control transcription elongation by RNA polymerase II in Saccharomyces cerevisiae. Genes Dev. *12*, 357–369.

Haruki, H., Nishikawa, J., and Laemmli, U.K. (2008). Technique The Anchor-Away Technique : Rapid, Conditional Establishment of Yeast Mutant Phenotypes. Mol. Cell *31*, 925–932.

Kecman, T., Kuś, K., Heo, D.H., Duckett, K., Birot, A., Liberatori, S., Mohammed, S., Geis-Asteggiante, L., Robinson, C.V., and Vasiljeva, L. (2018). Elongation/Termination Factor Exchange Mediated by PP1 Phosphatase Orchestrates Transcription Termination. Cell Rep. *25*, 259–269.e5.

Kent, N.A., Karabetsou, N., Politis, P.K., and Mellor, J. (2001). In vivo chromatin remodeling by yeast ISWI homologs Isw1p and Isw2p. Genes Dev. *15*, 619–626.

Kornberg, R.D. (1974). Chromatin structure: a repeating unit of histones and DNA. Science *184*, 868–871.

Krasnopolsky, S., Novikov, A., Kuzmina, A., and Taube, R. (2021). CRISPRi-mediated depletion of Spt4 and Spt5 reveals a role for DSIF in the control of HIV latency. Biochim. Biophys. Acta. Gene Regul. Mech. *1864*, 194656.

Kujirai, T., and Kurumizaka, H. (2020). Transcription through the nucleosome. Curr. Opin. Struct. Biol. *61*, 42–49.

Langmead, B. (2010). Aligning short sequencing reads with Bowtie. Curr. Protoc. Bioinformatics *Chapter 11*, Unit 11.7.

Lawrence, M., Huber, W., Pagès, H., Aboyoun, P., Carlson, M., Gentleman, R., Morgan, M.T., and Carey, V.J. (2013). Software for computing and annotating genomic ranges. PLoS Comput. Biol. *9*, e1003118.

Li, H. (2011). Improving SNP discovery by base alignment quality. Bioinformatics *27*, 1157–1158.

Longtine, M.S., McKenzie, A., 3rd, Demarini, D.J., Shah, N.G., Wach, A., Brachat, A., Philippsen, P., and Pringle, J.R. (1998). Additional modules for versatile and economical PCR-based gene deletion and modification in Saccharomyces cerevisiae. Yeast *14*, 953–961.

Love, M.I., Huber, W., and Anders, S. (2014). Moderated estimation of fold change and dispersion for RNA-seq data with DESeq2. Genome Biol. *15*, 550.

Mason, P.B., and Struhl, K. (2005). Distinction and relationship between elongation rate and processivity of RNA polymerase II in vivo. Mol. Cell *17*, 831–840.

Massey, F.J. (1951). The Kolmogorov-Smirnov Test for Goodness of Fit. J. Am. Stat. Assoc. *46*, 68.

McKay, M.D., Beckman, R.J., and Conover, W.J. (1979). A Comparison of Three Methods for Selecting Values of Input Variables in the Analysis of Output from a Computer Code. Technometrics *21*, 239.

Mischo, H.E., and Proudfoot, N.J. (2013). Disengaging polymerase: terminating RNA polymerase II transcription in budding yeast. Biochim. Biophys. Acta *1829*, 174–185.

Morillon, A., Karabetsou, N., O'Sullivan, J., Kent, N., Proudfoot, N., and Mellor, J. (2003). Isw1 chromatin remodeling ATPase coordinates transcription elongation and termination by RNA polymerase II. Cell *115*, 425–435.

Ocampo, J., Chereji, R.V., Eriksson, P.R., and Clark, D.J. (2016). The ISW1 and CHD1 ATP-dependent chromatin remodelers compete to set nucleosome spacing in vivo. Nucleic Acids Res. *44*, 4625–4635.

Ocampo, J., Chereji, R.V., Eriksson, P.R., and Clark, D.J. (2019). Contrasting roles of the RSC and ISW1/CHD1 chromatin remodelers in RNA polymerase II elongation and termination. Genome Res. *29*, 407–417.

Parua, P.K., Booth, G.T., Sansó, M., Benjamin, B., Tanny, J.C., Lis, J.T., and Fisher, R.P. (2018). A Cdk9-PP1 switch regulates the elongation-termination transition of RNA polymerase II. Nature *558*, 460–464.

Peck, S.A., Hughes, K.D., Victorino, J.F., and Mosley, A.L. (2019). Writing a wrong: Coupled RNA polymerase II transcription and RNA quality control. Wiley Interdiscip. Rev. RNA *10*, e1529.

Pelechano, V., Wei, W., and Steinmetz, L.M. (2013). Extensive transcriptional heterogeneity revealed by isoform profiling. Nature *497*, 127–131.

Ponting, C.P. (2002). Novel domains and orthologues of eukaryotic transcription elongation factors. Nucleic Acids Res. *30*, 3643–3652.

Rimel, J.K., and Taatjes, D.J. (2018). The essential and multifunctional TFIIH complex. Protein Sci. *27*, 1018–1037.

Rosen, G.A., Baek, I., Friedman, L.J., Joo, Y.J., Buratowski, S., and Gelles, J. (2020). Dynamics of RNA polymerase II and elongation factor Spt4/5 recruitment during activator-dependent transcription. Proc. Natl. Acad. Sci. USA *117*, 32348–32357.

Sainsbury, S., Bernecky, C., and Cramer, P. (2015). Structural basis of transcription initiation by RNA polymerase II. Nat. Rev. Mol. Cell Biol. *16*, 129–143.

Schneider, C.A., Rasband, W.S., and Eliceiri, K.W. (2012). NIH Image to ImageJ: 25 years of image analysis. Nat. Methods *9*, 671–675.

Shetty, A., Kallgren, S.P., Demel, C., Maier, K.C., Spatt, D., Alver, B.H., Cramer, P., Park, P.J., and Winston, F. (2017). Spt5 Plays Vital Roles in the Control of Sense and Antisense Transcription Elongation. Mol. Cell *66*, 77–88.e5.

Singh, A.K., and Mueller-Planitz, F. (2021). Nucleosome Positioning and Spacing: From Mechanism to Function. J. Mol. Biol. *433*, 166847.

Squazzo, S.L., Costa, P.J., Lindstrom, D.L., Kumer, K.E., Simic, R., Jennings, J.L., Link, A.J., Arndt, K.M., and Hartzog, G.A. (2002). The Paf1 complex physically and functionally associates with transcription elongation factors in vivo. EMBO J. *21*, 1764–1774.

Tufegdžić Vidaković, A., Mitter, R., Kelly, G.P., Neumann, M., Harreman, M., Rodríguez-Martínez, M., Herlihy, A., Weems, J.C., Boeing, S., Encheva, V., et al. (2020). Regulation of the RNAPII Pool Is Integral to the DNA Damage Response. Cell *180*, 1245–1261.e21.

van der Weegen, Y., Golan-Berman, H., Mevissen, T.E.T., Apelt, K., González-Prieto, R., Goedhart, J., Heilbrun, E.E., Vertegaal, A.C.O., van den Heuvel, D., Walter, J.C., et al. (2020). The cooperative action of CSB, CSA, and UVSSA target TFIIH to DNA damage-stalled RNA polymerase II. Nat. Commun. *11*, 2104.

Van Oss, S.B., Cucinotta, C.E., and Arndt, K.M. (2017). Emerging Insights into the Roles of the Paf1 Complex in Gene Regulation. Trends Biochem. Sci. *42*, 788–798.

Venkatesh, S., and Workman, J.L. (2015). Histone exchange, chromatin structure and the regulation of transcription. Nat. Rev. Mol. Cell Biol. *16*, 178–189.

Vos, S.M., Farnung, L., Linden, A., Urlaub, H., and Cramer, P. (2020). Structure of complete Pol II-DSIF-PAF-SPT6 transcription complex reveals RTF1 allosteric activation. Nat. Struct. Mol. Biol. *27*, 668–677.

Weiner, A., Hughes, A., Yassour, M., Rando, O.J., and Friedman, N. (2010). High-resolution nucleosome mapping reveals transcription-dependent promoter packaging. Genome Res. *20*, 90–100.

Xu, J., Wang, W., Xu, L., Chen, J.Y., Chong, J., Oh, J., Leschziner, A.E., Fu, X.D., and Wang, D. (2020). Cockayne syndrome B protein acts as an ATP-dependent processivity factor that helps RNA polymerase II overcome nucleosome barriers. Proc. Natl. Acad. Sci. USA *117*, 25486–25493.

Zatreanu, D., Han, Z., Mitter, R., Tumini, E., Williams, H., Gregersen, L., Dirac-Svejstrup, A.B., Roma, S., Stewart, A., Aguilera, A., and Svejstrup, J.Q. (2019). Elongation Factor TFIIS Prevents Transcription Stress and R-Loop Accumulation to Maintain Genome Stability. Mol. Cell *76*, 57–69.e9.

Zhang, Y., Liu, T., Meyer, C.A., Eeckhoute, J., Johnson, D.S., Bernstein, B.E., Nusbaum, C., Myers, R.M., Brown, M., Li, W., and Liu, X.S. (2008). Model-based analysis of ChIP-Seq (MACS). Genome Biol. *9*, R137.

Zhang, X., Smits, A.H., van Tilburg, G.B.A., Ovaa, H., Huber, W., and Vermeulen, M. (2018). Proteome-wide identification of ubiquitin interactions using UbIA-MS. Nat. Protoc. *13*, 530–550.

Zhou, K., Gaullier, G., and Luger, K. (2019). Nucleosome structure and dynamics are coming of age. Nat. Struct. Mol. Biol. *26*, 3–13.

Zhu, W., Wada, T., Okabe, S., Taneda, T., Yamaguchi, Y., and Handa, H. (2007). DSIF contributes to transcriptional activation by DNA-binding activators by preventing pausing during transcription elongation. Nucleic Acids Res. *35*, 4064–4075.

STAR★METHODS

KEY RESOURCES TABLE

REAGENT or RESOURCE	SOURCE	IDENTIFIER
Antibodies		
Monoclonal ANTI-FLAG® M2 antibody	Sigma-Aldrich	F3165; RRID: AB_259529
Anti-GFP-antibody	Abcam	Cat# ab290; RRID:AB_303395
Anti-RNA polymerase II subunit B1 (phospho-CTD Ser-5) Antibody, clone 3E8	Millipore	Cat# 04-1572-I; RRID:AB_2801296
Bacterial and virus strains		
pFA6a-3-FLAG-His3MX6	Fischl et al., 2017	N/A
pFA6a-FRB-yEGFP-hygromycin	Holstege Lab	N/A
Chemicals, peptides, and recombinant proteins		
ANTI-FLAG M2 Affinity Gel antibody	Sigma-Aldrich	Cat#A2220; RRID:AB_10063035
RQ1 RNase-free DNase I	Promega	M6101
3X FLAG Peptide	Sigma-Aldrich	F4799
miRNeasy Mini Kit	QIAGEN	Cat#217004
T4 RNA Ligase 2 truncated	New England Bio Labs	M0242
Gel Loading Buffer II	Invitrogen	AM8546G
10% Mini-PROTEAN TBE-Urea Gel	BIO-RAD	Cat#4566033
SYBR Gold Nucleic Acid Gel Stain	Invitrogen	S11494
Corning Costar Spin-X centrifuge tube filters	Corning	CLS8162
GlycoBlue Coprecipitant	Invitrogen	AM9516
SuperScript III Reverse Transcriptase	Invitrogen	Cat#18080044
SUPERase.In RNase Inhibitor	Invitrogen	AM2694
CircLigase ssDNA Ligase	Cambio	CL4115K
Phusion® High-Fidelity PCR Master Mix with HF Buffer	New England Bio Labs	M0531S
8% TBE Gel	Novex	EC62155BOX
Zymolyase 20T	MP biomedical	Cat#08320921
1M HEPES Solution	Fisher Scientific	Cat#10204932
Nuclease S7	Roche	Cat#10107921001
cOmplete, EDTA-free Protease Inhibitor Cocktail	Roche	Cat#11836170001
PhosSTOP 10 tablets	Roche	Cat#4906845001
Dynabeads Protein A for Immunoprecipitation	Invitrogen	10002D
Dynabeads Protein G for Immunoprecipitation	Invitrogen	10003D
Rapamycin	LC laboratories	R-5000
Pierce Silver Stain Kit	Thermo Fisher Scientific	Cat#24612
Zymoclean ChIP concentrator kit	Zymo Research	D5201
Critical commercial assays		
NEBNext Ultra II DNA Library Prep Kit	New England Bio Labs	E7103
NextSeq 500/550 High Output Kit v2.5 (75 Cycles)	Illumina	Cat# 20024906
Deposited data		
NET-seq, ChIP-seq, TEF-seq, and MNase-seq	This study	GEO:GSE159291

(*Continued on next page*)

Continued		
REAGENT or RESOURCE	SOURCE	IDENTIFIER
Experimental models: Organisms/strains		
S. cerevisiae strains	This study	Table S1
S. pombe Rpb9-3xFLAG	Vasilieva Lab	Table S1
Oligonucleotides		
Library construction, ChIP-qPCR, RT-PCR, and gene tagging	IDT	Table S2
Software and Algorithms		
Galaxy Web-based platform	Usegalaxy.org	RRID:SCR_006281
FastQC	https://www.bioinformatics.babraham.ac.uk/projects/fastqc/	RRID:SCR_014583
Bowtie for Illumina	Langmead, 2010	RRID:SCR_005476
Model-based Analysis for ChIP-Seq -MACS2	Zhang et al., 2008	RRID:SCR_013291
R Project for Statistical Computing	R studio	https://www.r-project.org/; RRID:SCR_001905
Bioconductor	Gentleman et al., 2004	RRID:SCR_006442
GenomicRanges	Bioconductor	RRID:SCR_000025
GenomicFeatures	Lawrence et al., 2013	RRID:SCR_016960
DEseq2	Love et al., 2014	RRID:SCR_015687
DEP	Zhang et al., 2018	https://bioconductor.org/packages/release/bioc/html/DEP.html
DANPOS2	Chen et al., 2013	RRID:SCR_015527
MATLAB	The MathWorks Inc.	RRID:SCR_001622
Other		
Bioscreen C MCR	Oy Growth Curves Ab Ltd	RRID:SCR_007172
Qubit Fluorometer	Thermo Fisher Scientific	RRID:SCR_018095
2100 Bioanalyzer Instrument	Agilent	RRID:SCR_018043
ImageJ	Schneider et al., 2012	RRID:SCR_003070

RESOURCE AVAILABILITY

Lead contact

Further information and requests for resources and reagents should be directed to and will be fulfilled by the lead contact, Jane Mellor (jane.mellor@bioch.ox.ac.uk)

Materials availability

Yeast strains (Table S3) generated in this study are available on request from the lead contact.

Data and code availability

The datasets generated during this study are available at GEO:GSE159291. The PRO-seq (GEO:GSE76142) and *paf1Δ* NET-seq (ArrayExpress: E-MTAB-4568) datasets were downloaded and reanalyzed as part of this study.

The original code for mathematical model is provided at https://github.com/aangel-code/spt4_transcription_simulation.

Any additional information required to reanalyze the data reported in this paper is available from the lead contact upon request.

EXPERIMENTAL MODEL AND SUBJECT DETAILS

Yeast strains and culturing

BY4741 derived *S.cerevisiae* cells were pre-cultured in YPD (1% yeast extract, 1% peptone, and 2% glucose) overnight at 30°C. The overnight culture was used to inoculate appropriate volume of YPD culture at OD_{600} 0.2, which was grown (30°C, 160 rpm) to OD_{600} 0.6-0.7 for all experiments unless stated otherwise. *S.pombe* cells were cultured in YES (0.5% yeast extract, 0.0225% of each aa: L-Adenine, L-Histidine, L-leucine, L-Lysine HCL, Uracil, and 3% glucose) in the same way as *S.cerevisiae* cells.

All strains used in this study, and the plasmids used to construct new strains for this study, are listed in Table S3. C terminus tagging of the proteins was performed by using the homologous recombination method (Longtine et al., 1998). PCR products were amplified with a 40 bp sequence homologous to the first 40 bp upstream of the stop codon of the gene to be tagged followed by a tag sequence, selection marker and 40bp of sequence homologous to a region downstream of the gene to be tagged (see Table S4 for primers).

METHOD DETAILS

NET-seq/TEF-seq

Cell growth and immunoprecipitation

2 L of cells were grown in YPD to OD_{600} 0.65 (30°C, 160 rpm shaking), collected by filtering and flash frozen in liquid nitrogen. 1.28 g of frozen *S.cerevisiae* pellet was combined with 0.32 g of frozen *S.pombe* pellet. The combined pellet was ground with mixer mill (6 cycles, 3 min, 15 hz) in a metal chamber with a metal ball and the chamber was submerged into liquid nitrogen between the milling runs. IPs were carried out in the cold room, all buffers used were ice-cold and all centrifugations were at 4°C. 1 g of grindate was resuspended in 5.66 mL of Lysis Buffer A (20 mM HEPES (pH 7.4), 110 mM KOAc, 0.5% Triton X-100, 0.1% Tween 20, 10 mM MnCl2, 1x proteinase inhibitors (Roche; complete, EDTA-free), 50 U/ml SUPERase.In RNase inhibitors (Invitrogen), 132 U/ml DNase I (Promega)) by continuous pipetting up and down for several minutes. The lysate was incubated in ice for 20 min and then centrifuged (16,000 g, 10 min). The supernatant was taken and 400 μl of M2 agarose beads pre-washed twice with 10 mL Lysis Buffer A (without SUPERase.In and DNase I) was added to the supernatant. IPs were performed on a rotating wheel for 2.5 h and then washed 4 times for 2 min with 10 mL Wash Buffer A (20 mM HEPES (pH 7.4), 110 mM KOAc, 0.5% Triton X-100, 0.1% Tween 20, 1 mM EDTA). Excess wash buffer was removed by centrifugation (1,000 g, 2 min). Samples were eluted twice with 300 μl 1 mg/ml of 3xFLAG peptide (Sigma) (prepared in Lysis Buffer A without SUPERase.In and DNase I) for 30 min by mild rotation. Eluates were collected by centrifugation (1,000 g, 2 min) and combined. RNAPII bound RNA was isolated with QIAGEN miRNA kit according to the manufacturer's instructions, RNA was eluted in 31 μl of elution buffer. 1 μl of the sample was used to measure RNA amount in Nanodrop. During the IP, 20 μl of samples were taken from the input, unbound (the first flow through after 2.5 h IP incubation) and eluate samples, and mixed with 20 μl of 2x SDS buffer (100 mM Tris-Cl pH 6.8, 20% glycerol, 4% SDS, 0.1% bromophenol blue, 200 mM DTT) for western blot controls.

Library preparation: Adaptor ligation and fragmentation

A minimum of 2.5 μg of immunoprecipitated RNA was diluted in 30 μl H_2O, split into 3 tubes and denatured (2 min, 80°C) and placed on ice (2min). RNA was ligated with 5′end adenylated and 3′end blocked adaptor (Table S4) by adding 10 μl of ligation mix (50 ng/μl cloning linker 1, 12% PEG 8000, 1 x T4 RNA ligase2 truncated ligation buffer, 10 U/μl T4 RNA ligase2 (truncated) (NEB)) to each tube (3 h, 37°C). Then the reaction was stopped by adding 0.7 μl of 0.5 M EDTA. Adaptor ligated RNA was fragmented by adding 20 μl of Alkaline Fragmentation Buffer (AFB; 100 mM NaCO3 (pH 9.2), 2 mM EDTA) (35-40 min, 95°C). Exact incubation time was determined for each batch of AFB. Then 0.56 mL RNA precipitation buffer (500 μL H_20, 60 μL 3M NaOAc (pH 5.5), 2 μL 15 mg/ml GlycoBlue (Ambion)) and 0.75 mL isopropanol was added, and samples were incubated at −20°C (> 30 min). RNA was collected by centrifuge (20,000 g, 30 min,4°C) and washed with cold 80% EtOH. RNA in three tubes was resuspended in the same 10 μl of 10 mM Tris-HCl pH 7.0.

Library preparation: RNA size selection

Adaptor ligated, and fragmented RNA was mixed with 10 μl gel loading bufferII (Invitrogen), denatured (2 min, 80°C) and placed on ice (3 min). Denatured RNA was run on 10% TBE-Urea gel (Biorad) (200 V, 35 min) in 1 x TBE buffer (diluted from RNase-free 10 X TBE (Ambion)). The gel was stained with SybrGold (Invitrogen) (5 min, RT) and RNA corresponding to 40-90nt was excised. For physical disruption, the gel slices were spun through 0.5 mL tubes with holes at the bottoms nested in 1.5 mL tubes. The disrupted gel slurry was incubated in 600 μl water (10 min, 70°C, 1400rpm shaking). RNA was cleared from the gel by transferring the mix into a Costar-spin column (Corning) and centrifuging (20,000 g, 3 min, RT). 50 μl 3 M Sodium Acetate (pH 5.5), 2 μl Glycoblue and 0.75 mL of isopropanol was added to RNA mix and incubated at −20°C (> 30 min). RNA was collected by centrifugation (20,000 g, 30 min, 4°C), washed with 0.75 mL cold 70% EtOH and resuspended in 10 μl of 10 mM Tris pH 7.0.

Library preparation: Reverse transcription (RT)

Size selected RNA was mixed with 4.6 μl of RT mix (3.28 μL 5 x First-Strand buffer, 0.82 μL dNTPs (10 mM each), 0.5 μL 100 μM RT primer (Table S4)) and denatured (2 min, 80°C). Then 1.32 μl Superase.In/DTT and 0.82 μl SuperScriptIII added and incubated (30 min, 48°C). 1.8 μl 1 M NaOH added (20 min, 98°C) to degrade RNA. 1.8 μl 1M HCl added after the incubation to neutralize the cDNA.

Library preparation: cDNA size selection

cDNA was mixed with 20 μl gel loading buffer II (Invitrogen), denatured (3 min, 95°C) and placed on ice (3 min). Denatured cDNA was run on 10% TBE-Urea gel (Biorad) (200 V, 50 min) in 1xTBE buffer. The gel was stained with SybrGold (Invitrogen) (5 min, RT) and cDNA corresponding to 80-130 nt was excised. For physical disruption, the gel slices were spun through 0.5 mL tubes with holes at the bottoms nested in 1.5 mL tubes. The disrupted gel slurry was incubated in 400 μl water (10 min, 70°C, 1400 rpm shaking). cDNA was cleared from the gel by transferring the mix into a Costar-spin column (Corning) and centrifuging (20,000 g, 3 min, RT). 25 μl 3 M NaCl, 2 μl Glycoblue and 0.75 mL of isopropanol was added to the cDNA mix. Samples were incubated at −20°C (> 30 min). cDNA

was collected by centrifuge (20,000 g, 30 min, 4°C), washed with 0.75 mL cold 80% EtOH and resuspended in 15 μl of 10 mM Tris-HCl (pH 8.0).

Library preparation: Circularization

4 μl circularization mix (2 μL 10 x CircLigase buffer, 1 μL 1 mM ATP, 1 μL 50 mM $MnCl_2$) and 1 μl of CircLigase (Epicenter) was added to the size selected cDNA and incubated (60 min, 60°C). Then the enzyme was heat inactivated (10 min, 80°C).

Library preparation: Amplification and barcoding

Circularized cDNA was amplified and barcoded (Table S4) by adding 15 μl of PCR master mix (8 μl HF master mix (NEB), 0.8 μl 10 μM reverse barcoding primer, 0.8 μl 10 μM forward barcoding primer, 5.4 μl water) per 1 μl template (1 cycle: 30 s 98°C;; 3-to-7 cycles: 10 s 98°C; 10 s 60°C; 5 s 72°C;; 1 cycle: Hold 4°C). Tubes were taken at the end of 3-4-5-7 cycles. PCR products were mixed with 3μl loading dye (NEB) and run on 8% TBE gel (Invitrogen) (90 V, 95 min) in 1xTBE buffer. The gel was stained with SybrGold (Invitrogen) (5 min, RT) and DNA corresponding to 120-170 nt was excised. For physical disruption, the gel slices were spun through 0.5 mL tubes with holes at the bottoms nested in 1.5 mL tubes. Then 0.67 mL DNA soaking buffer (0.3 M NaCl, 10 mM Tris-HCl pH 8.0, 1 mM EDTA) was added to the gel slurry and tubes were incubated overnight on a rotating wheel.

Sequencing and data analysis

Barcoded libraries were pooled and sequenced on Illumina NextSeq 500 (50cycle, single-end) with custom reading primer (Table S4). Single-end FASTQ files were processed using usegalaxy.org and RStudio. Reads were groomed using FASTQ groomer for Sanger & Illumina 1.8 + (Blankenberg et al., 2010). Adaptor sequence ATCTCGTATGCCGTCTTC were trimmed and reads < 15 nt were discarded using Clip function. Reads were aligned to a combined fasta file of *S.cerevisiae* and *S.pombe* genomes using Bowtie for Illumina (Langmead, 2010). SAM files were converted to BAM files using SAM-to-BAM (Li, 2011). Using RStudio/Bioconductor packages (Gentleman et al., 2004), multiply aligned reads were filtered, and reads were narrowed to the 3′ends. Selected reads were annotated to the *S.cerevisiae* genes derived by TIF-seq (Pelechano et al., 2013).

No tag normalization

NET-seq was performed on strains without a FLAG-tag to detect background signal during the IP. As the *SCR1* gene is transcribed by RNAPIII and gives a high non-specific signal in both the FLAG-tagged and no tag NET-seq and TEF-seq IPs, and this locus was used for no-tag normalization. The reads on chrV [442007:442458] were split into 10 nt bins and FLAG-tag over no tag sample ratio is calculated for each bin. The mean *SCR1* ratio then multiplied by the no tag data and subtracted from the FLAG-tag samples.

$$\text{FLAG}-\text{tag}-[\text{Mean}SCR1\text{ratio}(\text{FLAG}-\text{tag}/\text{no tag})]\text{x no tag}$$

Spike in normalization

NET-seq data were aligned to the combined genome of Cer3 and Pombe. After the removal of non-uniquely aligned reads and no-tag background signal, counts table was created for *S.pombe* genes by using RStudio/Bioconductor (Gentleman et al., 2004; Lawrence et al., 2013). Then estimateSizeFactors function in the DEseq2/RStudio package was applied to calculate the relative amounts of *S.pombe* reads (i.e., normalization ratio) in each sample (Love et al., 2014). NET-seq data were calibrated by dividing *S.cerevisiae* reads by the normalization ratios.

NET-seq/TEF-seq metagene plots

Protein-coding genes (PCGs) > 750 nt were taken and genes with negative values due to no tag normalization were discarded. To avoid genes with wrong TSS annotation, genes having 1.5x more reads upstream of the TSS (−150 to 0 nt) than in the downstream (+1 to 150 nt) were also discarded. PCGs were plotted relative to the TSS in a window of TSS-250 nt to TSS+750 nt or relative to the PAS in a window of PAS-250 nt to PAS+250 nt. The mean number of counts for each nt position was calculated excluding top and bottom 1% of reads to avoid random spikes introduced during sequencing. The mean number of counts then was split into 10 nt bins for the metagene plots.

ChIP-seq

50 mL of cells were grown in YPD to OD600 0.6 (30°C, 160 rpm), collected by centrifuge (1,000 g, 4min) and resuspended in 45 mL of 1x PBS. Samples were crosslinked by addition of 1.25 mL of 37% formaldehyde (1% final) at RT for 5 min with shaking at 85 rpm on a rocker. Then the reaction was quenched by the addition of 2.5 mL of 2.5 M glycine (0.125 M final) at RT for 5 min with shaking at 85 rpm. Cells were pelleted (1,000 g, 4 min, 4°C) and washed twice with 10 mL of 1x PBS (1,000 g, 2 min, 4°C). Pellets were resuspended in cold FA-150 buffer (10 mM HEPES pH 7.9, 150 mM NaCl, 0.1% SDS, 0.1% sodium deoxycholate, 1% Triton X-100) and mixed with pre-crosslinked with *S.pombe* in 5:1 ratio (final *S.pombe* percentage being 16.7%). The cell suspension was lysed with glass beads using the MagnaLyser (Roche; 6 × 45 s runs, 2500 g, 4°C). The lysate was sheared 30-40 min with a Bioruptor sonicator 30 s ON/30 s OFF at high setting. The sheared lysate was cleared by centrifuge (10,000 g, 15 min, 4°C) and the supernatant was used for IP. 500 μL sample was incubated with ~100 μg (25 μl) of the FLAG (M2) in 1.5 mL siliconized Eppendorf tubes for 15–20 h rotating at 4°C. When the IP was performed for ChIP-qPCR, 50 μL sample was diluted to 200 μL with FA-150 buffer and incubated with 5 μL (~20 μg) of the GFP antibody in 1.5 mL siliconized Eppendorf tubes for 15–20 h rotating at 4°C. Bound chromatin was immunoprecipitated for 90 min at 22°C with 50 μL protein A or G-Dynabeads pre-blocked with bovine serum albumin and sonicated salmon sperm DNA. Beads and immunoprecipitated chromatin were pelleted by centrifugation (640 g, 1 min) and washed with 400 μl of TSE-150 buffer (20 mM Tris–Cl pH 8.0, 150 mM NaCl, 2 mM EDTA, 0.1% SDS, 1% Triton X-100) for 3 min, 400 μl of TSE-500 buffer (20 mM Tris-Cl pH 8.0, 500 mM NaCl, 2 mM EDTA, 0.1% SDS, 1% Triton X-100) for 3 min, 400 μl of LiCl buffer (0.25 M LiCl, 10 mM

Tris-Cl pH 8.0, 1 mM EDTA, 1% deoxycholate, 1% NP-40) for 15 min and twice with 400 μl of TE. Following the washes, chromatin was eluted for 30 min at 65°C with 100 μl of elution buffer (0.1 M NaHCO3, 1% SDS). For reverse crosslinking, 7 μl of 5 M NaCl was added (3 h, 65°C). Next, samples were treated with 1 μl of 10 mg/ml RNase A for 1 h at 37°C and 2 μl of 20 mg/ml proteinase K overnight at 65°C. DNA was eluted with Zymoclean ChIP concentrator kit according to the manufacturer's instructions. DNA concentrations were measured by qubit and libraries prepared with the NEBNext Ultra II DNA Library Prep Kit for Illumina according to the manufacturer's instructions. Barcoded libraries were pooled and sequenced on Illumina NextSeq 500 (75 cycle, paired). Paired FASTQ files were processed using usegalaxy.org. Illumina adapters were trimmed using Trim Galore!. Reads were aligned to SacCer3 and Pombe genomes using Bowtie2. Aligned reads were filtered to remove PCR duplicates using RmDp and filtered for quality reads MAPQ > 20 using Filter SAM or BAM. To normalize reads to Pombe spike-ins, normalization ratio was calculated to obtain the same amount of filtered Pombe BAM reads in each sample, and SacCer3 BAM reads were calibrated using Downsample SAM/BAM accordingly. ChIP-seq peaks were obtained using MACS2 callpeak (Zhang et al., 2008), and the background signal was subtracted using MACS2 bdgcmp.

Proteomics Sample Collection and Analysis

The IP was performed as in the NET-seq IP with the following modifications; 1 L of cells were grown instead of 2 L and the working volumes were halved accordingly. Lysis and wash buffers were supplemented with 1 x PhosSTOP (Roche). After the first four washes as in NET-seq, the fifth wash was performed for 20 min in wash buffer A. Next, four more washes were performed with wash buffer A-150 (20 mM HEPES (pH 7.4), 110 mM KOAc, 0.5% Triton X-100, 0.1% Tween 20, 1 mM EDTA, 150 mM NaCl) for 1 min each for the first three and 30 min for the final wash.

Mass spectrometry and data analysis

200 μl of the eluate was submitted to Advanced Proteomics Facility at University of Oxford, Department of Biochemistry. Samples were Trypsin FASP digested with detergent. Raw MS data files were analyzed with MaxQuant with (< 1% FDR) and searched against *S.cerevisiae* database. For protein quantification, LFQ intensities were used. Proteins with less than 2 peptides in FLAG IP experiments were discarded. Data were imputed and p values were calculated with DEP package /RStudio (Zhang et al., 2018). Significant proteins met the criteria of $\log_2$FC(*spt4Δ*/WT) > 2 and p.adj < 0.01.

Silver Staining

Protein samples were separated on 4%–20% precast gradient SDS polyacrylamide gel (Biorad) by gel electrophoresis at 100 V for 2 h. Silver staining was performed with Pierce Silver Stain Kit (Thermo Fisher) according to the manufacturer's instructions. Briefly, the gel was washed with MQ water (10 min) and fixed in 30% ethanol 10% acetic acid (30 min). After washing with 10% ethanol (10 min) and MQ water (10 min), it was sensitized (1 min), stained (30 min) and developed (2-3 min) with the buffers provided in the kit. The developed gel was fixed with 5% acetic acid (10 min).

Anchor Away

Rapamycin treatment

2.3 L of cells were grown in YPD to OD_{600} 0.3 (30°C, 160 rpm) and DMSO or 1 mg/ml rapamycin dissolved in DMSO added. For ChIP, 45 mL of cells collected at 0, 60, 140 min (or at 0, 60, and 180 min for Spt5) after rapamycin treatment. For fluorescence microscopy, 13 mL of cells collected at 0, 60, 140 min (or at 0, 60, and 180 min for Spt5).

ChIP-qPCR

Rapamycin-treated samples were cross-linked with 1% FA (10 min, RT). Then the reaction was quenched for 5 min with the addition of 2.5 mL of 2.5 M glycine. Cells were pelleted (1,000 g, 2.5 min, 4°C) and washed twice with 10 mL cold PBS (137 mM NaCl, 2.7 mM KCl, 8 mM Na_2HPO_4, and 2 mM KH_2PO_4). Immunoprecipitation of ChIP was performed as described above. qPCR was performed using a Corbett Rotorgene and Sybr green mix (Bioline) for *RPL3* and *PGK1* loci (see Table S4 for primers). Signal was computed using %input method.

Immunofluorescence microscopy

Rapamycin-treated samples were kept in a falcon tube wrapped with aluminum foil to limit light exposure as much as possible. Then the harvested cells were cross-linked with 4% PFA (40 min, RT), pelleted (1,000 g, 2.5 min, 4°C) and washed twice with 5 mL of cold buffer B (1.2 M sorbitol, 100 mM $KHPO_4$ pH 7.5). The pellet was resuspended in residual buffer B after the second centrifuge, and 200 μl of the suspension placed on poly-L-lysine coated coverslips and incubated (30 min, 4°C). Then coverslips were washed by dipping into MQ water twice and mounted on slides with a drop of ProLong Diamond Antifade Mountant with DAPI (Vector Shield). Slides were left at RT overnight in the dark and corners of the coverslips sealed with transparent nail polish. Slides were imaged with DeltaVision CORE wide-field fluorescence deconvolution microscope using a 100x/1.4 objective lens, T%32 filter, with exposure times of 0.05 s for DAPI and 1 s for FITC channels, respectively. For NET-seq, cells were grown to OD_{600} 0.65 (140 min for Spt4 depletion, 180 min for Spt5 depletion and 120 min for DMSO control) and 2L of cells were harvested as described above for NET-seq. Images were processed using ImageJ (Schneider et al., 2012).

Doubling time measurement and analysis

Overnight cultures were diluted to OD_{600} 0.10-0.15 in 250 μl YPD and grown in 100 well plates for the Bioscreen (22 h, 30°C), with readings at OD_{600} taken every 20 min with shaking (200 rpm). For the anchor away testing, YPD is supplemented with DMSO or 1 mg/ml rapamycin dissolved in DMSO. A minimum of four technical replicates were performed for each condition and strain.

OD_{600} measurements were analyzed in R. Reads were blanked by subtracting medium-only reads. Doubling times were calculated by choosing the exponential growth phase (OD_{600} 0.2 to 0.7) and using the following equation: Doubling time = log (2)*time/ [log (max (OD_{600}) – log (min (OD_{600}))]

MNase-seq

MNase-seq protocol

Cell nuclei was prepared as described (Almer et al., 1986).1 L of cells were grown in YPD to OD_{600} 0.6 (30°C, 160 rpm), collected by filtering and resuspended in 45 mL of cold water. Then cells were pelleted by centrifuge (1,000 g, 5min, 4°C). After discarding the water, the weight of cells (wet weight) was noted and the following volumes were used per 1 g of wet cells. 2 mL of pre-incubation solution (2.8 mM EDTA, pH 8, 0.7 M 2-mercaptoethanol) was added to wet cells and incubated (30°C, 30 min). Then samples were pelleted (1,000 g, 5 min, 4°C) and the pellet was washed with 40 mL of 1 M sorbitol. After centrifuging (1,000 g, 5 min, 4°C) and discarding sorbitol, the pellet was resuspended in 5 mL of sorbitol/B-ME (1 M sorbitol, 5 mM 2-mercaptoethanol) solution and 200 μl of 2% of zymolase solution at 30°C for 30 min with shaking. The lysate was pelleted (3,000 g, 8 min, 4°C) and the pellet was washed with 40 mL of 1 M sorbitol. Nuclei were resuspended in 7 mL of Ficoll solution (18% Ficoll, 20 mM KH_2PO_4, 1 mM $MgCl_2$, 0.25 mM EGTA, 0.25 mM EDTA) then collected by centrifuge (20,000 g, 30 min, 4°C). The nuclei obtained from 0.5 g equivalent of cells were resuspended in 3 mL of freshly prepared SDB (1 M sorbitol, 50 mM NaCl, 10 mM Tris-Cl pH 7.5, 5 mM $MgCl_2$, 1 mM $CaCl_2$, 0.075% NP-40, 1 mM 2-mercaptoethanol), split into 6 tubes. 6 reactions were set up with 20-40-80-160-320U of 10U/μl MNase (prepared in 200mM Tris pH 7.5, 50 mM NaCl, 50% glycerol; Nuclease S7 Roche) and incubated (37°C, exactly 10 min). Reactions were quenched with 50 μl of pre-warmed stopping buffer (5% SDS, 50 mM EDTA, at 65°C). 50 μl of 20 mg/ml proteinase K (Roche) added to MNase treated samples and incubated (overnight, 65°C). Samples were treated with 1μl of 10 mg/ml RNase A (1 h, 37°C) and then DNA was eluted with Zymoclean ChIP concentrator kit according to the manufacturer's instructions. Isolated DNA was run on a 1.5% agarose-TBE gel and right digestion (80U) was chosen based on the enrichment of mono-nucleosome bands; faint di-nucleosome bands are still visible without over digestion. The mono-nucleosomal DNA band was gel extracted. DNA concentrations were measured by qubit and libraries prepared with the NEBNext Ultra II DNA Library Prep Kit for Illumina according to the manufacturer's instructions.

MNase-seq data analysis

Barcoded libraries were pooled and sequenced on Illumina NextSeq 500 (75 cycle, paired). Paired FASTQ files were processed using usegalaxy.org. Illumina adapters were trimmed using Trim Galore!. Reads were aligned to SacCer3 genome using Bowtie2. Aligned reads were filtered to remove PCR duplicates using RmDp and filtered for quality reads MAPQ > 20 using Filter SAM or BAM. BAM files were further analyzed using a peak calling software DANPOS2 (Chen et al., 2013). Read densities and nucleosome positions obtained from DANPOS2 were used for metagene analysis.

MNase-seq metagene analysis

Protein coding genes (PCGs) shorter than 600 nt were discarded. Genes with 4 peaks within the first 600 nt from the TSS (+1 to 600 nt) across the three replicates were included in the analysis to avoid genes with poorly phased nucleosomes. 2622 PCGs were left for the analysis.

Mathematical Modeling

The process of transcription was formulated as a stochastic process with core components of initiation; elongation; polymerase occlusion; stalling; resumption of elongation from the stalled state; backtracking from a stalled state; resumption of elongation from the backtracked state; collision-induced stalling and termination; early termination with a Poisson distribution around a fixed location; two dynamic windows, in which there can be different stalling, backtracking, and resumption rates; termination at the 3′ end of a gene. When polymerases collide, the situation resolves itself depending on the state of the polymerases involved. If a moving polymerase collides with another moving polymerase, the upstream polymerase becomes stalled. If a moving polymerase collides with a stalled polymerase, the upstream polymerase will become stalled and the downstream stalled polymerase will be terminated. If a moving polymerase collides with a backtracked polymerase, the upstream polymerase will become stalled.

The simulations were limited to the beginning of a synthetic gene, which covered 1000 nt. Polymerases had a fixed footprint of 40 nt. Upon reaching the end of the synthetic gene and elongating, polymerases were removed. 150,000 parameter sets were sampled uniformly between the maximum and minimum parameter values given in Table S1, via latin-hypercube sampling (McKay et al., 1979) and each of these was simulated for a population of 100,000 identical synthetic genes. Simulations were run for the equivalent of 40 minutes in increments of 0.005 minutes per time-step to allow the system to reach a steady state. The output distribution of transcriptionally-engaged polymerase for a given parameter set was then taken of the sum of the locations of polymerases at the final time-step of each of the 100,000 simulated genes. For the purposes of fitting, simulation and experimental data were binned with bins of size 10 nt. For the experimental NET-seq data, genes were defined via annotations derived from TIF-seq (Pelechano et al., 2013): the TSS and TTS for each gene was defined by choosing the most abundant start and end point detected in YPD. Only genes longer than 1000 nt in length were selected and, of those, only ones with total read counts in the first 1000 nt greater than the average for all initially selected genes. Experimental and simulated NET-seq data were normalized by dividing each bin by the total read counts or sampled polymerase locations in the first 1000 nt, respectively. Simulated data were compared to NET-seq data using the Kolmogorov-Smirnov statistic (maximum of the differences between individual points of the CDF of each dataset;

Massey, 1951) as the goodness-of-fit metric. For the plots in Figure 2B, the single best fitting simulation for each gene was used; for the parameter comparisons, the 100 best fitting simulations for each gene were used.

QUANTIFICATION AND STATISTICAL ANALYSIS

Number (n) of biological or technical repeats is given in the legend for each figure. A biological repeat is a separate experiment using a fresh aliquot of a yeast strain conducted at a different time. Technical repeats are conducted on the same material as part of the same experiment.

N is the number of genes subjected to statistical analysis after meeting criteria for inclusion or exclusion described in each legend or in the STAR Methods.

Statistical Analysis

Details are given in the figure legend for each experiment. In summary for growth assays error bars indicate standard deviation of 3 biological replicates performed at 4 technical repeats. * p value < 0.05, ***p value < 0.001 (Student's t test, unpaired, two-tailed or Student's t test, paired, two sided). For boxplots, p < 0.001, two-tailed, paired Student's t test. For proteomics the significance for enrichment or depletion of factors requires the following criteria: log2(fold change spt4Δ/WT) > 2 and p-adj < 0.05. Differential enrichment of factors on RNAPII used DEseq2 applied to the read counts from the gene body (TSS to TSS-250 nt) for two replicates of each dataset and requires a p-adjusted < 0.05. A one tailed (condition: upstream signal < downstream signal), paired Student's t test was used to determine significance of the change in factor occupancy on RNAPII around nucleosomes.

ADDITIONAL RESOURCES

There are no additional resources associated with this study.

Cell Reports, Volume *36*

Supplemental information

Spt4 facilitates the movement of RNA polymerase II through the +2 nucleosomal barrier

Ülkü Uzun, Thomas Brown, Harry Fischl, Andrew Angel, and Jane Mellor

Figure S1, related to Figure 1

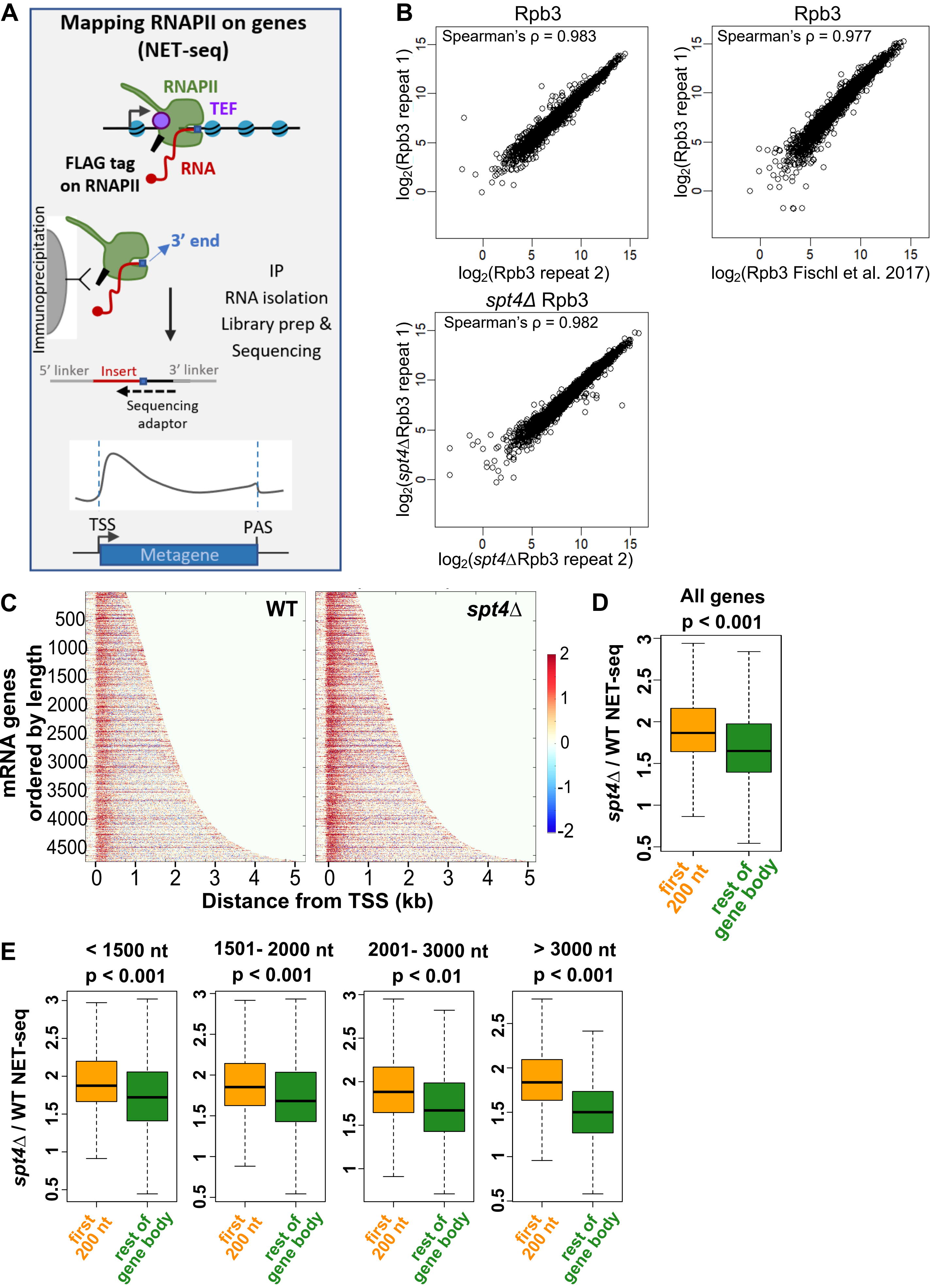

Figure S1, related to Figure 1

In the absence of Spt4, RNAPII accumulates at the 5'end of genes

A Native elongating transcript sequencing (NET-seq) pulls down elongation competent RNAPII and the 3'-end sequencing allows mapping of RNAPII at single nucleotide resolution.

B Correlations between NET-seq repeats from this study and with published NET-seq data from (Fischl et al., 2017). Reads are counted from the TSS to the PAS for each gene. Log_2 transformed gene counts are correlated and Spearman's ρ calculated for each pair.

C Heatmaps of the WT (left) and *spt4*Δ (right) NET-seq signal. Each row indicates a PCG (N=4610), ranked by gene length. The colour code from red to blue reflects the changes in the RNAPII signal for each nucleotide position from TSS-250 nt to TSS+4750 nt (x-axis) as shown by the colour bar.

D Boxplots of the *spt4*Δ / WT NET-seq ratios within the first 200 nt reads from the TSS (from TSS to TSS+200 nt; orange) and the rest of the gene body (from TSS+200 to PAS-250 nt; green) for protein-coding genes after filtering low read genes out (see Methods). N=4610, $p < 0.001$, two-tailed, paired Student's t-test.

E The same analysis in D repeated based on the gene length with the following subgroups < 1500 nt (N=1174), 1501 – 2000 nt (N=1192), 2001-3000 nt (N=1453), and > 3000 nt (N=791).

Figure S2, related to Figure 3

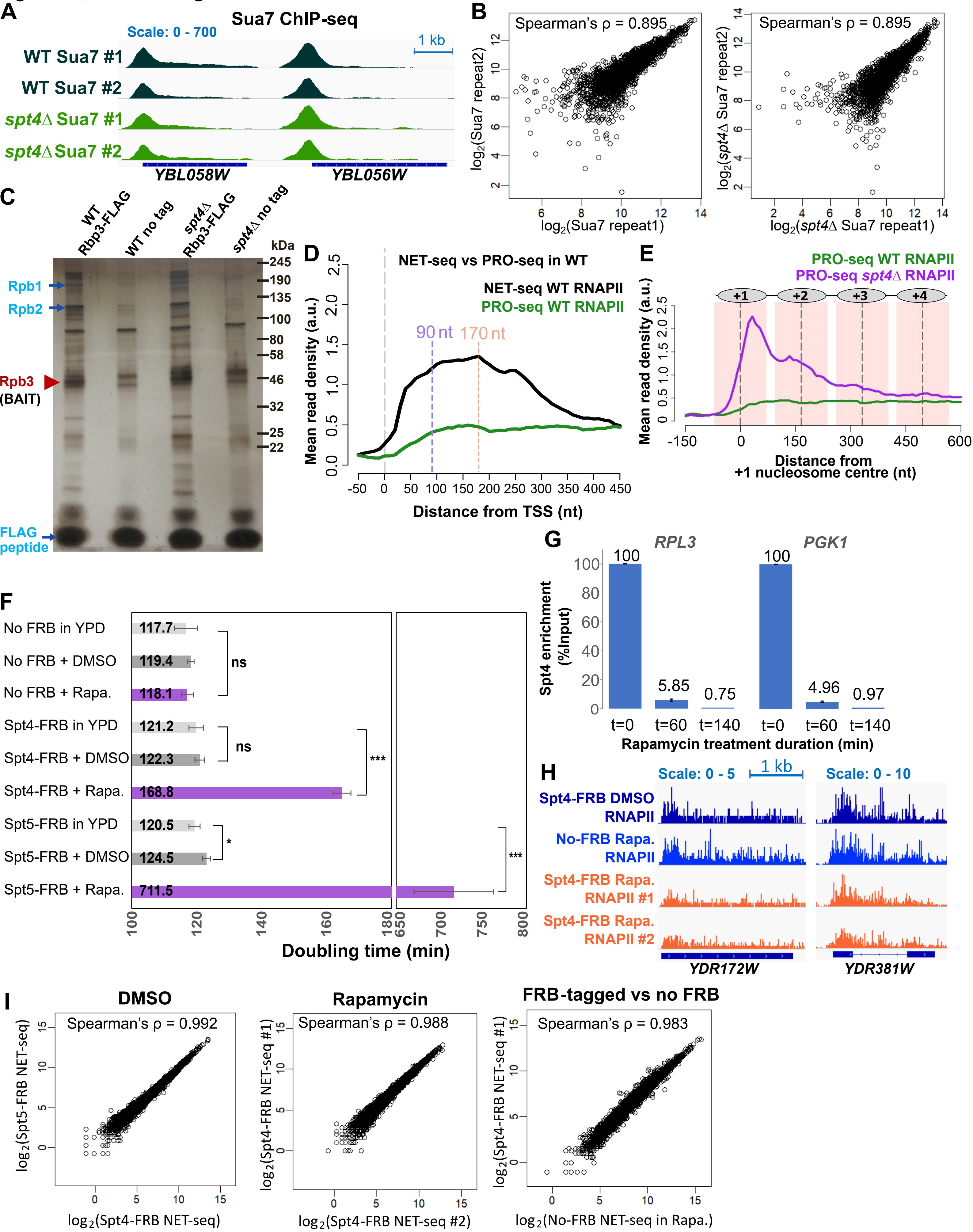

Figure S2, related to Figure 3

The primary defect in *spt4*∆ cells is early transcription elongation

A WT and *spt4*∆ Sua7 ChIP-seq signals of example genes transcribed from the positive strand: *YBL058W* and *YBL056W* in two biological replicates. The dark blue boxes indicate the transcribed region of the genes (from TSS to PAS).

B Correlation plot of the two repeats of each experiment. Reads are counted around the TSS (TSS-100 to TSS+100) for each gene. $\log_2$ transformed gene counts are correlated and Spearman's ρ calculated for each pair.

C Silver staining of purified transcription complexes for proteomics analysis. After purification of the complexes, a small portion of the purified samples was run on 4-20% gradient SDS-PAGE and the protein enrichment was checked using a silver stain.

D Metagene plots of WT NET-seq (black) and WT PRO-seq (green) reads (Booth et al., 2016) aligned at the TSS, the same data as in **Figure 3E**. Dashed lines indicate the highest *spt4*∆ PRO-seq (90 nt, purple) and *spt4*∆ NET-seq reads (170 nt, pink) for comparison with **Figure 3F**.

E Metagene of RNAPII distribution assessed using PRO-seq in *spt4*∆ cells reveals a peak on the upstream face of the +2 nucleosome in addition to a larger peak at +90 nt from the TSS compared to WT (a paused/stalled/backtracked RNAPII that has been released into elongation during the labelling window (see also **Figure 3F**). This peak is also a component of the NET-seq profile, except the RNAPII signal continues to accumulate indicative of a form of RNAPII that is not undergoing active elongation during the labelling window for PRO-seq.

F Doubling times of the anchor away strains. Cells were grown in YDP, DMSO and rapamycin (1 mg/ml in DMSO) for 22 h. OD_{600} was recorded every 20 min using the Bioscreen and doubling times were calculated for exponential growth phase (OD_{600} 0.2 to 0.8) as described in the methods. Error bars indicate standard deviation of 3 biological replicates performed at 4 technical repeats. * p-value <0.05, ***p-value <0.001 (Student's t-test, unpaired, two-tailed).

G ChIP-qPCR for Spt4 upon depletion of Spt4 protein by Anchor Away across different time points. Percentage of Spt4 levels relative to time point 0 levels at the two representative genes *RPL3* and *PGK1* tested by ChIP against GFP (targeting Spt4-FRB-GFP) followed by qPCR. Error bars indicates standard deviation of the two biological replicates.

H DMSO or rapamycin-treated Spt4-FRB or rapamycin-treated No FRB NET-seq signals of example genes transcribed from the positive strand: *YDR172W* and *YDR381W*. Two biological replicates are shown for rapamycin treated cells. The dark blue boxes indicate the transcribed region of the genes (from TSS to PAS), while the blue line indicates the intronic region in *YDR381W.*

I Correlations between anchor away NET-seq repeats and controls. Reads are counted from the TSS to the PAS for each gene. Log_2 transformed gene counts are correlated and Spearman's ρ calculated for each pair.

Figure S3, related to Figure 4

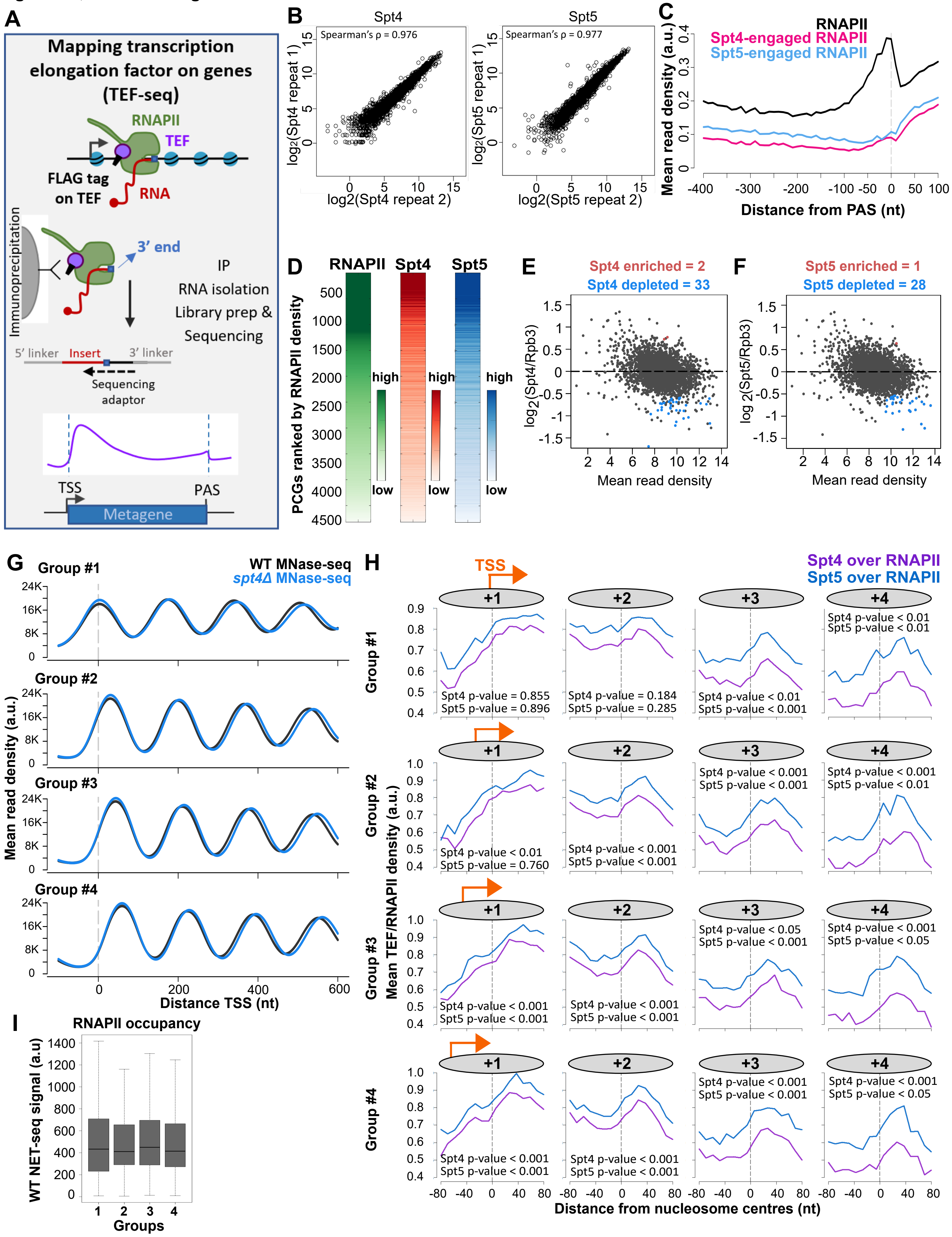

Figure S3, related to Figure 4

Spt4/5 travel with RNAPII and oscillate on and off RNAPII based on the nucleosome positions

A Similar to NET-seq (**Figure S1A**), transcription elongation factor (TEF) associated nascent elongating transcript sequencing (TEF-seq) pulls down elongation competent RNAPII from FLAG-tagged TEF. The 3'-end sequencing maps TEF-associated RNAPII at single nucleotide resolution.

B Correlations between TEF-seq repeats. Reads are counted from the TSS to the PAS for each gene. Log_2 transformed gene counts are correlated and Spearman's ρ calculated for each pair.

C Metagene plots of NET-seq (RNAPII; black), and TEF-seq (Spt4; pink, Spt5; light blue) reads around PAS. (Close up version of **Figure 4B**).

D Heatmaps of RNAPII NET-seq and Spt4/5 TEF-seq reads over the gene bodies (taken as TSS to PAS-250 nt) on log_2 scale. Protein-coding genes are ranked by RNAPII levels.

E, F Differential enrichment of Spt4 (**E**) and Spt5 (**F**) on RNAPII. DEseq2 applied to the read counts from the gene body (TSS to TSS-250 nt) for two replicates of each data. Significantly enriched and depleted genes indicated in red and blue, respectively (p-adjusted <0.05).

G Genes were ranked based on the location of their +1 nucleosome, relative to the transcription start site and split into 4 groups (N=581 per group).

H TEF-seq/NET-seq ratio relative to the centre of +1 to +4 nucleosomes in each of the four groups.

I Boxplots of WT NET-seq reads showing RNAPII occupancy over genes in groups 1 to 4 (see **G**).

Figure S4, related to Figure 5

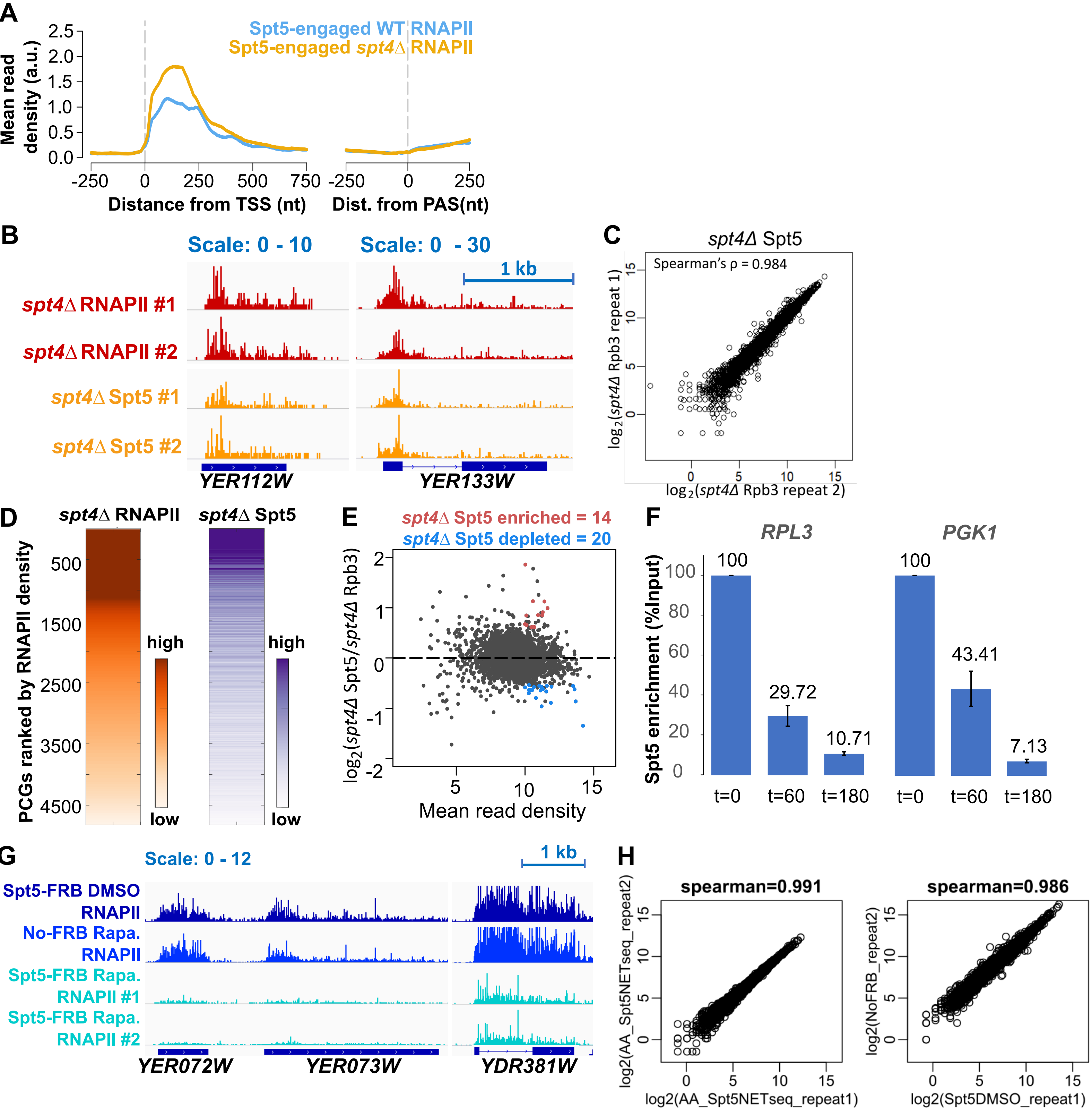

Figure S4, related to Figure 5

Spt5 and Spt4 have distinct impacts on transcription

A Metagene plots of WT Spt5 TEF-seq (light blue; Spt5-engaged WT RNAPII the same as in **Figure 4B**), and *spt4∆* Spt5 TEF-seq (yellow; Spt5-engaged *spt4∆* RNAPII) reads aligned at the TSS or PAS.

B *spt4∆* NET-seq (RNAPII) and *spt4∆* Spt5 TEF-seq reads of example genes transcribed from the positive strand: *YER112W* and *YER113W* in two biological replicates. The dark blue boxes indicate the transcribed region of the genes (from TSS to PAS), while the blue line indicates the intronic region in *YER113W.*

C Correlations between *spt4∆* Spt5 TEF-seq repeats. Reads are counted from the TSS to the PAS for each gene. Log_2 transformed gene counts are correlated and Spearman's ρ calculated for each pair.

D Heatmaps of *spt4∆* RNAPII NET-seq and *spt4∆* Spt5 TEF-seq reads over the gene bodies (taken as TSS to PAS-250 nt) on log_2 scale. Protein-coding genes are ranked by RNAPII levels.

E Differential enrichment of *spt4∆* Spt5 on *spt4∆* RNAPII. DEseq2 applied to the read counts from the gene body (TSS to TSS-250 nt) for two replicates of each data. Significantly enriched and depleted genes indicated in red and blue, respectively (p-adjusted <0.05).

F ChIP-qPCR for Spt5 upon depletion of Spt5 protein by Anchor Away across different time points. Percentage of Spt5 levels relative to time point 0 levels at the two representative genes *RPL3* and *PGK1* tested by ChIP against GFP (targeting Spt5-FRB-GFP) followed by qPCR. Error bars indicates standard deviation of the two biological replicates.

G DMSO or rapamycin-treated Spt5-FRB or rapamycin treated No FRB NET-seq signals of example genes transcribed from the positive strand: *YER072W, YER073W* and *YDR381W*. Two biological replicates are shown for rapamycin treated cells. The dark blue boxes indicate the transcribed region of the genes (from TSS to PAS), while the blue line indicates the intronic region in *YDR381W.*

H Correlations between anchor away NET-seq repeats and controls. Reads are counted from the TSS to the PAS for each gene. Log_2 transformed gene counts are correlated and Spearman's ρ calculated for each pair.

Figure S5, related to Figure 4 and 6

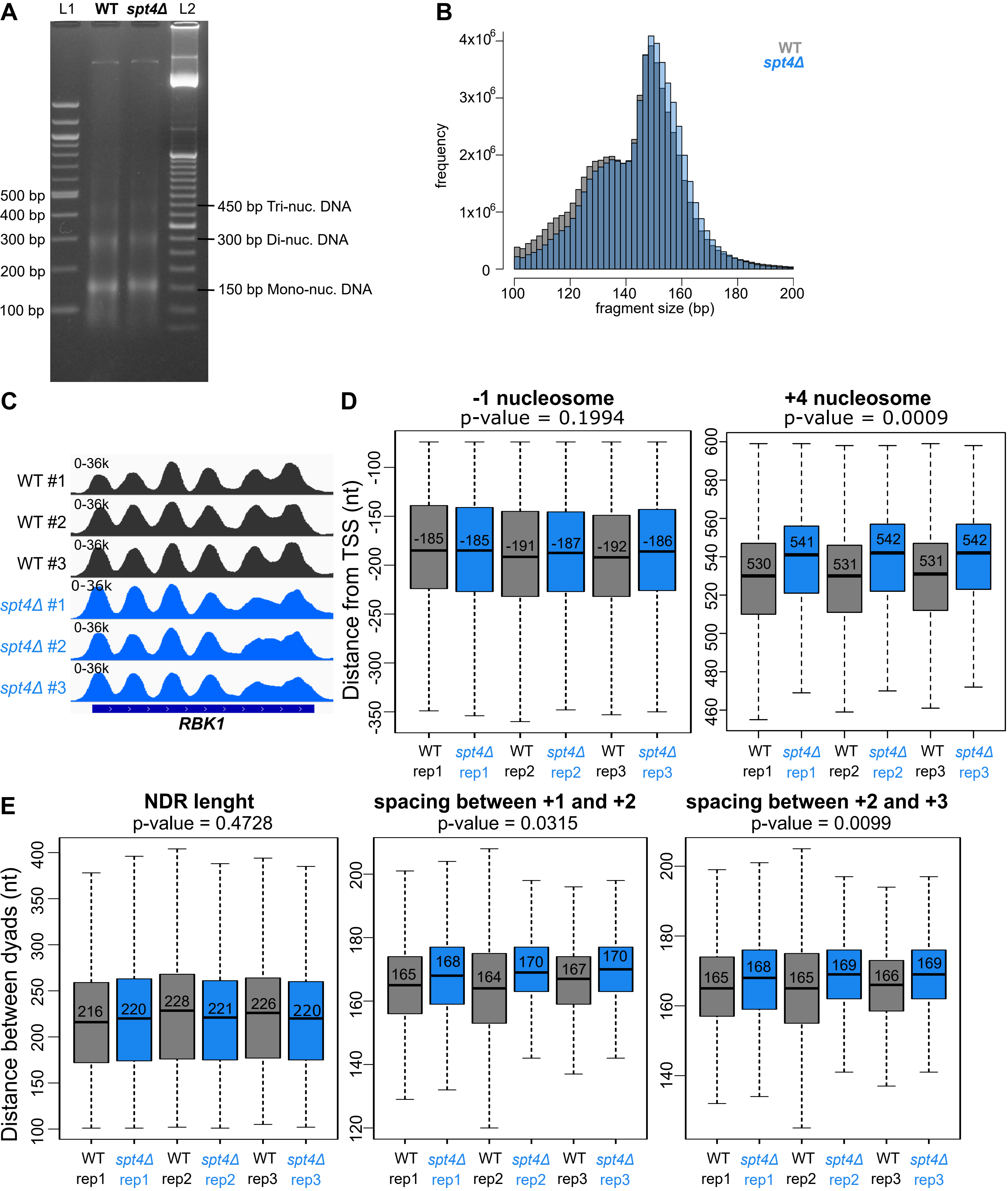

Figure S5, related to Figures 4 and 6
Spt4 influences nucleosome positioning
A WT and *spt4*Δ chromatin samples were digested with 80U MNase and separated by 1.5% agarose-TBE gel electrophoresis. 80U MNase digestion led to the optimal digestion pattern as shown in (Chereji et al., 2019). L1: 100 bp ladder, L2: 50 bp ladder. Bands corresponding to mono-nucleosomal DNA (~150 bp) were excised and subjected to paired-end sequencing.
B Histograms of MNase-seq fragment size in WT (grey) and *spt4*Δ (blue) data sets. Consistent with the digestion pattern in A, the majority of the sequenced fragments represent mono-nucleosomal fragments (~150 bp) in both data sets, giving the optimal digestion pattern.
C MNase-seq reads of example genes transcribed from the positive strand (*RBK1*) in WT and *spt4*Δ in 3 biological replicates. The dark blue boxes indicate the transcribed region of the genes (from TSS to PAS).
D Box-plots of the distance of the -1 and +4 nucleosomes from the TSS in three biological replicates of WT (black) and *spt4*Δ cells (blue). Numbers in the boxes indicate the median position of the given nucleosome. p-values were calculated by comparing the median position of the -1 or +4 nucleosomes in WT and *spt4*Δ conditions obtained from each replicate (Student's t-test, paired, two sided).
E Box-plots of the distance between the -1 and +1 (NDR length), +1 and +2, +2 and +3 nucleosomes in three biological replicates of WT (black) and *spt4*Δ cells (blue). Numbers in the boxes indicate the median distance between the indicated nucleosomes. p-values were calculated by comparing the median distances between the nucleosomes in WT and *spt4*Δ conditions obtained from each replicate (Student's t-test, paired, two sided).

Figure S6, related to Figure 7

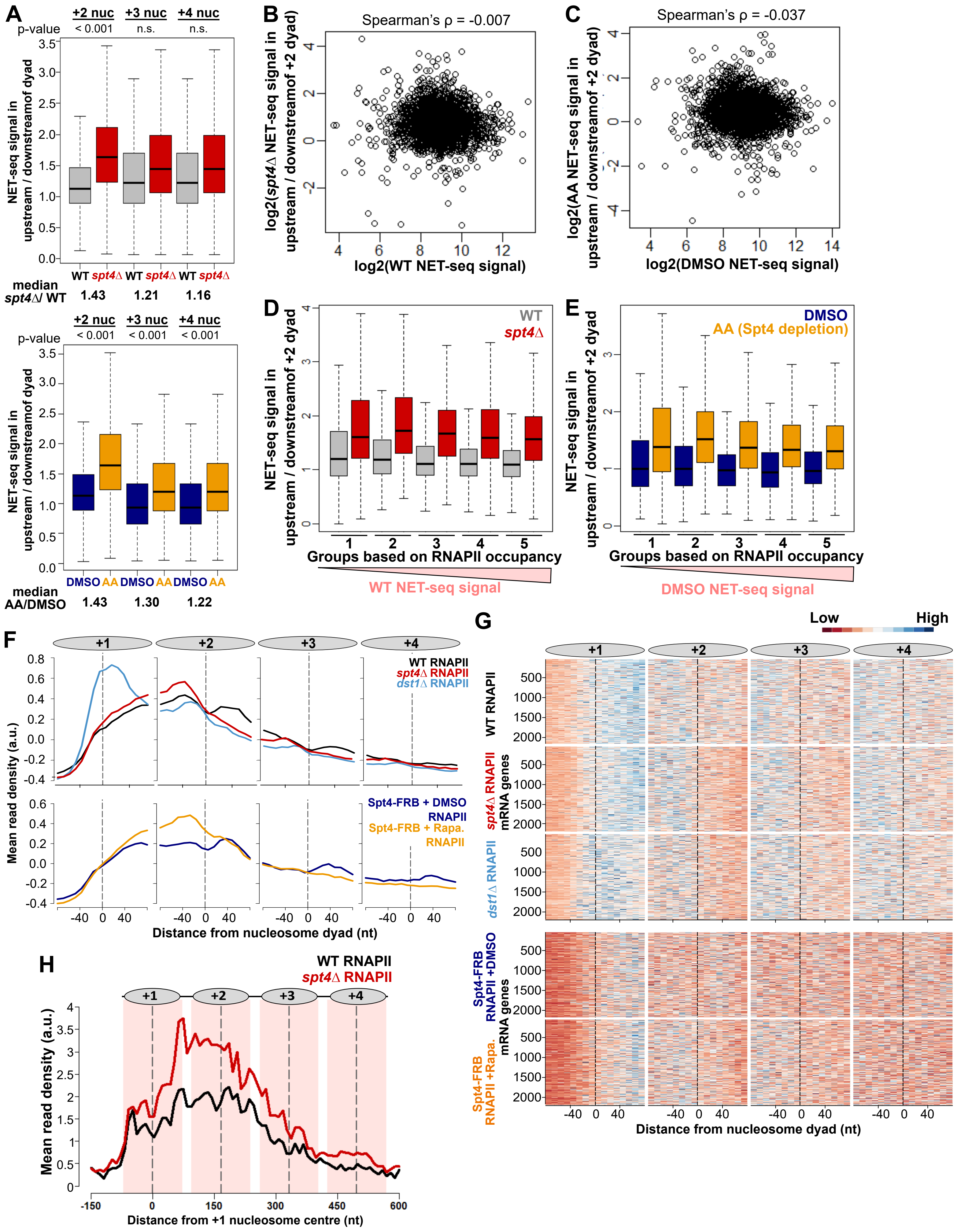

Figure S6, related to Figure 7

The accumulation of RNAPII in the absence of Spt4 is associated with the position of the +2 nucleosome

A The WT (grey), *spt4*Δ (red), DMSO (navy), and Spt4 Anchor Away (AA; orange) RNAPII signal ratio from upstream of the dyad (-60 to -10 nt from the dyad) to downstream of the dyad (+10 to +60 nt from the dyad) were shown as boxplots for the +2, +3, and +4 nucleosomes. The difference of the accumulation in WT and *spt4*Δ or DMSO and Spt4 Anchor Away cells were tested by two-tailed, paired Student's t-test. To compare different nucleosomes, *spt4*Δ/WT or AA/DMSO RNAPII accumulation ratios around the +2, +3, and +4 nucleosomes are given at the bottom of the plots.

B,C Correlation plots showing the relationship between reads on the upstream or downstream face of the +2 nucleosome for *spt4*Δ (**B**) or Spt4-AA (**C**) and levels of transcription over each gene. $\log_2$ transformed gene counts are correlated for each gene and Spearman's ρ calculated. **D,E** 2312 genes were divided into 5 groups using the WT RNAPII signal (NET-seq) over the gene bodies after genes with very low NET-seq signal and with poor nucleosome phasing were filtered out as described in the Methods. RNAPII signal ratio of the proximal to distal reads around the +2 dyad was assessed in the *spt4Δ* strain compared to WT (**D**) or after Spt4 depletion (AA) compared to the DMSO control (**E**).

F Metagene plots of WT (black), *spt4*Δ (red), and *dst1*Δ (blue) (top panel) and DMSO-treated (navy), and rapamycin-treated Spt4-FRB (orange) (bottom panel) NET-seq profiles around the +1, +2, +3 and, +4 nucleosome dyads. Dashed lines (black) through the peaks indicate the centres of the nucleosomes (-/+ 80 nt from the dyad; x-axis). The NET-seq reads were normalised to the mean and standard deviation of each gene to indicate the shape of the distribution of RNAPII regardless of the expression level differences between the genes (N=2212), also shown in **G**. Position of nucleosomes graphically shown above the metagene plot.

G Heatmaps of WT, *spt4*Δ, *dst1*Δ, DMSO-treated, and rapamycin-treated Spt4-FRB NET-seq profiles around the +1, +2, +3 and, +4 nucleosomes. Each row indicates a PCG (n=2212). RNAPII signal is shown in 10 nt bins around the indicated nucleosome dyads (-/+ 80 nt from the dyad; x-axis). The NET-seq reads were normalised to the mean and standard deviation of each gene to indicate the shape of the distribution of RNAPII regardless of the expression level differences between the genes.

H Metagene plots of WT (black) and *spt4*Δ (red) NET-seq profiles from 43 genes with reduced Sua7 (TFIIB) occupancy at the promoter in *spt4Δ* (See **Figure 3B**), around the +1, +2, +3 and, +4 nucleosome dyads. Dashed lines (black) through the peaks indicate the centres of the nucleosomes (-/+ 80 nt from the dyad; x-axis). The NET-seq reads were normalised to the mean and standard deviation of each gene to indicate the shape of the distribution of RNAPII regardless of the expression level differences between the genes.

Figure S7, related to Figure 7

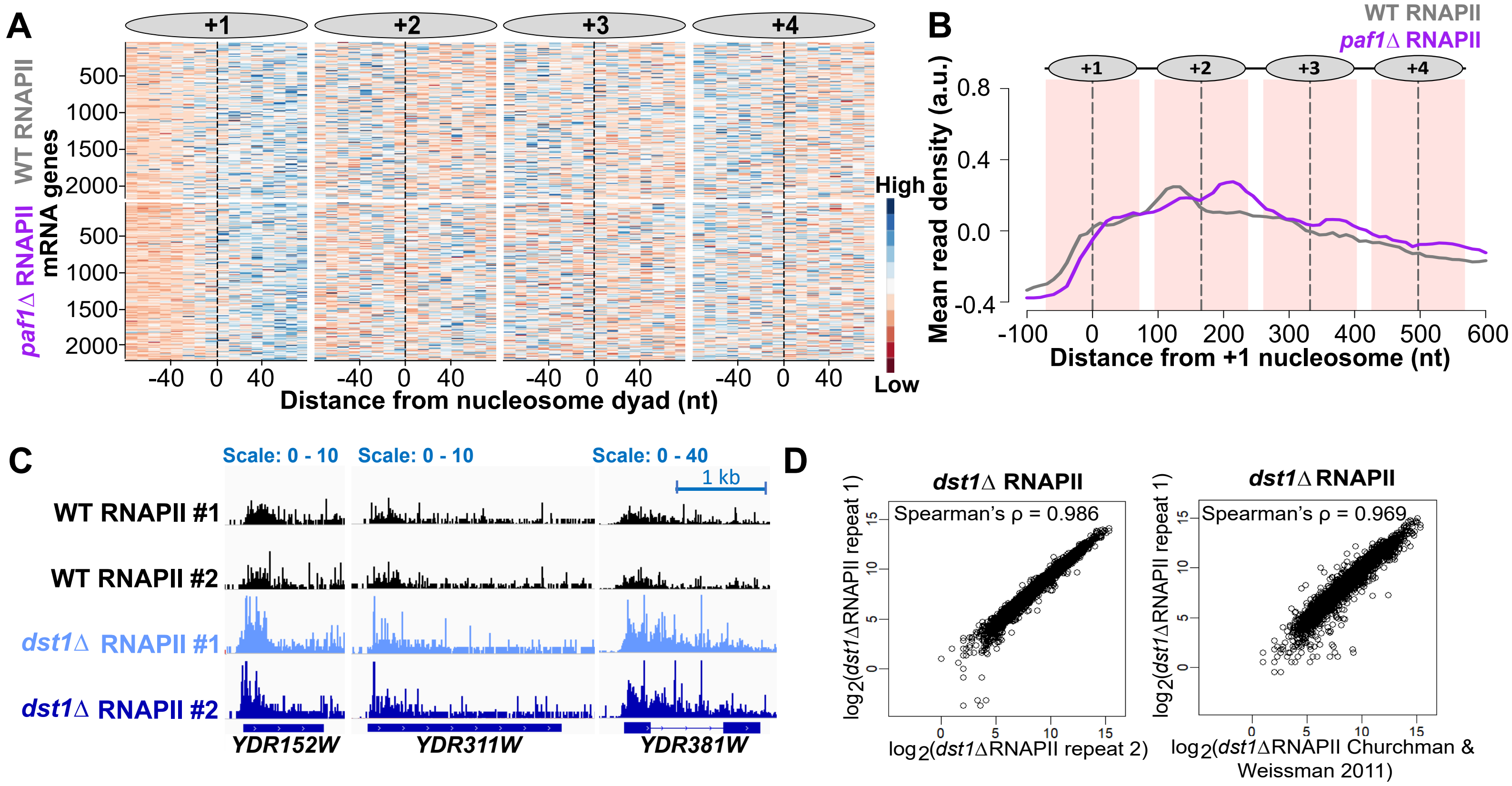

Figure S7 related to Figure 7

Comparison of the effect of loss of Paf1 and Dst1 on RNAPII accumulation

A Heatmaps of WT (top) and *paf1*Δ (bottom) NET-seq profiles around the +1, +2, +3 and, +4 nucleosomes. Each row indicates a PCG (N=2212). RNAPII signal is shown in 10 nt bins around the indicated nucleosome dyads (-/+ 80 nt from the dyad; x-axis). The NET-seq reads were normalised to the mean and standard deviation of each gene, so that the shape of the distribution of RNAPII could be seen more clearly regardless of the expression level differences between the genes. NET-seq datasets were taken from ArrayExpress: E-MTAB-4568 (Fischl et al., 2017).

B Metagene plots of WT (grey) and *paf1*Δ (purple) NET-seq profiles relative to the +1 nucleosome dyad of the same data as in **A**. The mean and standard deviation normalised reads were used for plotting metagene profiles, as the global comparison of the NET-seq levels were not available for these datasets. Dashed lines (black) through the peaks indicate the centres of the nucleosomes and the nucleosomal DNA (+/- 70 nt around the centre) is highlighted in light pink.

C NET-seq (RNAPII) reads of example genes transcribed from the positive strand: *YDR152W, YDR331W,* and *YDR381W* in two biological replicates in WT and *dst1*Δ cells. The dark blue boxes indicate the transcribed region of the genes (from TSS to PAS), while the blue line indicates the intronic region in *YDR381W.*

D Correlations between NET-seq repeats in *dst1*Δ cells from this study and with published NET-seq data from (Churchman and Weissman, 2011). Reads are counted from the TSS to the PAS for each gene. Log_2 transformed gene counts are correlated and Spearman's ρ calculated for each pair.

Table S1. Related to Figure 2
The maximum and minimum parameter values for the latin hypercube sampling

Parameter	Min Value	Max Value
Initiation Rate	0.01	60
Elongation Rate	100	5000
Stall 1 Rate	0.01	60
Stall Restart 1 Rate	0.01	60
Backtrack 1 Rate	0.01	60
Backtrack Restart 1 Rate	0.01	60
Stall 2 Rate	0.01	60
Stall Restart 2 Rate	0.01	60
Backtrack 2 Rate	0.01	60
Backtrack Restart 2 Rate	0.01	60
Location of Window Boundary	0	1000
Location of Early Termination	0	1000

Table S2. Related to Figure 3
Mass spec log fold enrichment analysis results

name	*spt4Δ* vs WT p.val	*spt4Δ* vs WT p.adj	*spt4Δ* vs WT ratio
Spt4	0.001	0.000	-3.690
Set2	0.021	0.000	-2.580
Spn1	0.047	0.003	-1.850
Spt16	0.032	0.001	-1.740
Pob3	0.063	0.007	-1.490
His3	0.449	0.414	-1.470
Gpn2	0.263	0.185	-1.260
Spt6	0.157	0.062	-1.250
Tma19	0.183	0.087	-1.170
Sub2	0.320	0.252	-1.150
Prp39	0.351	0.290	-1.110
Yra1	0.266	0.188	-1.100
Prp42	0.339	0.275	-1.070
Rpb3	0.242	0.158	-1.060
Bre1	0.496	0.466	-1.020
Rpc10	0.169	0.073	-0.989
Wtm1	0.241	0.157	-0.986
Asr1	0.474	0.442	-0.985
Hsh155	0.370	0.315	-0.953
Tex1	0.304	0.234	-0.835
Spt5	0.323	0.256	-0.767
Rse1	0.543	0.513	-0.754
Snu71	0.480	0.449	-0.718
Set1	0.718	0.645	-0.706
Prp40	0.514	0.485	-0.659
Tho2	0.571	0.539	-0.599
Smb1	0.571	0.539	-0.587
Rpb11	0.442	0.405	-0.566
Rba50	0.632	0.588	-0.563
Smd2	0.440	0.403	-0.547
Dst1	0.692	0.630	-0.522
Luc7	0.759	0.668	-0.492
Rpo21	0.548	0.518	-0.488
Hpr1	0.571	0.538	-0.466
Prp19	0.571	0.539	-0.461
Rpb2	0.575	0.542	-0.459
Snu114	0.722	0.648	-0.436
Hta2	0.576	0.543	-0.407
Rpb5	0.622	0.580	-0.346
Yhc1	0.707	0.638	-0.328
Smd3	0.688	0.627	-0.316
Rat1	0.714	0.643	-0.309
Rtt103	0.685	0.625	-0.291
Mft1	0.757	0.667	-0.278
Cbc2	0.731	0.653	-0.271
Rpb4	0.733	0.654	-0.245
Rpb8	0.735	0.655	-0.241
Rpo26	0.816	0.697	-0.162
Rpc19	0.864	0.717	-0.157
Thp2	0.891	0.728	-0.136
Tfg1	0.868	0.719	-0.127
Rpb9	0.880	0.724	-0.113
Sdc1	0.921	0.738	-0.100
Snu56	0.941	0.746	-0.087
Ceg1	0.968	0.754	-0.031
Mud1	0.986	0.760	-0.025

Sto1	0.991	0.761	-0.011
Ess1	0.994	0.762	-0.008
Rai1	0.985	0.759	0.030
Rpb7	0.963	0.752	0.032
Npa3	0.964	0.753	0.040
Tfg2	0.929	0.741	0.086
Smt3	0.794	0.686	0.178
Cet1	0.772	0.675	0.215
Rtr1	0.887	0.726	0.250
Abd1	0.774	0.676	0.320
Gpn3	0.680	0.622	0.443
Rad23	0.620	0.579	0.460
Taf14	0.538	0.508	0.494
Glc7	0.492	0.462	0.588
Ccl1	0.672	0.616	0.598
Bud27	0.416	0.374	0.729
Brr2	0.532	0.503	0.814
Yta7	0.281	0.207	1.270
Ald5	0.073	0.010	1.660
Ssl1	0.083	0.013	1.720
Sua7	0.094	0.017	1.930
Iwr1	0.102	0.020	1.960
Tfb2	0.130	0.039	2.120
Rad3	0.098	0.019	2.210
Tfb1	0.079	0.012	2.510
Tfb3	0.015	0.000	2.600
Ssl2	0.048	0.003	2.980
Yke2	0.026	0.000	4.120

Table S3. Related to STAR methods

Yeast strains used in this study

Strain	Source	Genotype
BY4741	Euroscarf	*MATa; his3Δ1; leu2Δ0; met15Δ0; ura3Δ0*
BY4741 *spt4::KanMX6 (SPT4 KO)*	Euroscarf	*spt4::KanMX6*
BY4741 Rpb3-FLAG (WT)	Fischl et al. 2017	*RPB3-3xFLAG-His3MX6*
BY4741 *SPT4 KO* Rpb3-FLAG (*spt4Δ*)	This study	*RPB3-3xFLAG-His3MX6; spt4::KanMX6*
BY4741 Spt4-FLAG	This study	*SPT4-3xFLAG-His3MX6*
BY4741 Spt5-FLAG	This study	*SPT5-3xFLAG-His3MX6*
BY4741 Sua7-FLAG	This study	*SUA7-3xFLAG-His3MX6*
BY4741 *SPT4 KO* Sua7-FLAG	This study	*SUA7-3xFLAG-His3MX6; spt4::KanMX6*
S.pombe Rpb9-FLAG	L. Vasileva	*u+; leu1-32; ura4Δ18; ade16-M216; his3Δ1; RPB9-3xFLAG-KanMX4*
AA Spt4-FRB-GFP Rpb3-FLAG	This study	*tor1-1; Δfpr1; RPL13-2xFKBP12-NATMX6; met15; LYS2; his3-1; leu2; ura3; MATa; SPT4-FRB-eGFP-HygMX*
AA Spt5-FRB-GFP Rpb3-FLAG	This study	*tor1-1; Δfpr1; RPL13-2xFKBP12-NATMX6; met15; LYS2; his3-1; leu2; ura3; MATa; SPT5-FRB-eGFP-HygMX*
AA Rpb3-FLAG (No FRB)	This study	*tor1-1; Δfpr1; RPL13-2xFKBP12-NATMX6; met15; LYS2; his3-1; leu2; ura3; MATa*
BY4741 *dst1::kanMX6*	Euroscarf	*dst1::kanMX6*
BY4741 *dst1::kanMX6* Rpb3-FLAG	This study	*RPB3-3xFLAG-His3MX6; dst1::kanMX6*

Table S4. Related to STAR methods
Oligonucleotides used in this study

Oligonucleotide	Sequence
Linker-1	5'AppCTGTAGGCACCATCAAT/3ddC 3'
RNA control oligo	5′ agu cac uua gcg aug uac acu gac ugu g3′
RT primer	5'/5Phos/ATCTCGTATGCCGTCTTCTGCTTG/iSp18/CACTCA/iSp18/TCCGACGATCATTGATGGTGCCTACAG 3'
Barcoding reverse	5'CAAGCAGAAGACGGCATACGA 3'
Barcode-1	5'AATGATACGGCGACCACCGAGATCTACACGATCGGAAGAGCACACGTCTGAACTCCAGTCACATGCCATCCGACGATCATTGATGG 3'
Barcode-2	5'AATGATACGGCGACCACCGAGATCTACACGATCGGAAGAGCACACGTCTGAACTCCAGTCACTGCATCTCCGACGATCATTGATGG 3'
Barcode-4	5'AATGATACGGCGACCACCGAGATCTACACGATCGGAAGAGCACACGTCTGAACTCCAGTCACTTAGGCTCCGACGATCATTGATGG 3'
Barcode-5	5'AATGATACGGCGACCACCGAGATCTACACGATCGGAAGAGCACACGTCTGAACTCCAGTCACTGACCATCCGACGATCATTGATGG 3'
Barcode-6	5'AATGATACGGCGACCACCGAGATCTACACGATCGGAAGAGCACACGTCTGAACTCCAGTCACGCCAATTCCGACGATCATTGATGG 3'
Barcode-7	5'AATGATACGGCGACCACCGAGATCTACACGATCGGAAGAGCACACGTCTGAACTCCAGTCACCAGATCTCCGACGATCATTGATGG 3'
Barcode-8	5'AATGATACGGCGACCACCGAGATCTACACGATCGGAAGAGCACACGTCTGAACTCCAGTCACACTTGATCCGACGATCATTGATGG 3'
Barcode-9	5'AATGATACGGCGACCACCGAGATCTACACGATCGGAAGAGCACACGTCTGAACTCCAGTCACGATCAGTCCGACGATCATTGATGG 3'
Barcode-10	5'AATGATACGGCGACCACCGAGATCTACACGATCGGAAGAGCACACGTCTGAACTCCAGTCACTAGCTTTCCGACGATCATTGATGG 3'
Sequencing primer	5'TCCGACGATCATTGATGGTGCCTACAG 3'
RPL3_forward	5' GGGTTACAAG GCTGGTATGA 3'
RPL3_reverse	5' ACAACGACAACTGGTGGAGT 3'
PGK1_forward	5' GCGTGTCTTC ATCAGAGTTG 3'
PGK1_reverse	5'AGTGAGAAGCCAAGACAACG 3'
RPB3_Cterm_FLAG_Forward	5'AATGGGTAATACTGGATCAGGAGGGTATGATAATGCTTGGACTAGTGGATCCCCCGGGGAT 3'
RPB3_Cterm_FLAG_Reverse	5'TTTCGGTTCGTTCACTTGTTTTTTTCCTCTATTACGCCCGAATTCGAGCTCGTTTAAAC 3'
SPT5-Cterm-FLAGtag-Forward	5'TAAGTCAAACTATGGTGGTAACAGTACATGGGGAGGTCATACTAGTGGATCCCCCGGGGAT 3'
SPT5_Cterm_FRBtag_Forward	5'TAAGTCAAACTATGGTG GTAACAGTACATGGGGA GGTCATCGGATCCCCGG GTTAATTAA 3'
SPT5-Cterm-tag-Reverse	5'GTCTTTTTTATTGATTTCTTCTTGGGTGATATTGGTTCTCGAATTCGAGCTCGTTTAAAC 3'
SUA7_Cterm_FLAG_Forward	5'TGTAGTGTCTTTGGATAACTTACCGGGCGTTGAAAAGAAAACTAGTGGATCCCCCGGGGAT 3'
SUA7_Cterm_FLAG_Reverse	5'TCTACCCTCTAACACGAGTACCCGTGCTTCTTGTTCCTATGAATTCGAGCTCGTTTAAAC 3'
SPT4-Cterm-FRBtag-Forward	5'GTTGCCTCACTACAAACCGAGGGATGGCAGTCAA GTTGAGCGGATCCCCGGGTTAATTAA 3'
SPT4-Cterm-tag-Reverse	5'ATTCATTACTATTATACATGTGATATCAGAACGGAAGGTTGAATTCGAGCTCGTTTAAAC 3'
